# Butterflies with low thermoregulatory capacity show greatest upwards range shifts along an elevational gradient

**DOI:** 10.1038/s42003-026-10534-z

**Published:** 2026-07-01

**Authors:** Esme Ashe-Jepson

**Affiliations:** Chair of Global Change Ecology, Biocenter, University of Würzburg, Würzburg, Germany

**Keywords:** Evolution, Entomology, Climate-change ecology

## Abstract

Climate change is a growing threat to global biodiversity, and so there is a pressing need to understand which traits impact species vulnerability, and the capacity of these traits to respond to environmental change. I quantify two thermal traits (thermoregulation, thermal tolerance) and functional traits (wing length, colouration, wing condition, sex) of butterflies across an elevational gradient in central Europe to investigate adaptation to local climatic conditions. There is no evidence of intraspecific variation in thermal traits, suggesting that these may be fixed within species. At low elevations, large species are better than small species at avoiding high body temperatures. Dark species have improved thermoregulatory capacities with decreasing elevation but ultimately have consistently higher body temperatures than pale species. This implies that small and dark species may be particularly vulnerable to extreme heat. I subsequently detect shifts from small dark species at high elevations to large pale species at low elevations. Finally, species with poor thermoregulatory capacity or larger wings have shown greater upwards elevational range shifts. This argues that butterflies are responding to climate change through redistribution of species rather than adaptation in place, and that thermoregulatory performance will be a key driver of ecological responses to climate change.

## Abstract (web summary)

Lack of variation in thermoregulatory capacity or thermal tolerance in butterflies across an elevational gradient suggests limited adaptive capacity. Poorer thermoregulators had stronger upwards range shifts in response to recent climate change.

## Introduction

Climate change is a growing threat to global biodiversity, with changes to mean temperatures and the increased frequency, intensity and longevity of extreme weather events causing species to experience novel thermal environments. Understanding how species respond to climate change is critical for informing effective conservation. Responses to climate change fall into three broad categories: extinction, dispersal, or adaptation in place—here used broadly to encompass both genetic change and phenotypic plasticity^1–3^. Dispersal results in the redistribution of species to track their climate niche, leading to novel community assemblages and interactions^4^, though it may be constrained by geographic barriers or lack of suitable habitat within range^5^. Alternatively, species can persist within their current distribution through in situ adaptation, either via genetic change or phenotypic plasticity. These responses are not mutually exclusive and are often difficult to disentangle empirically^6,7^. In the absence of either dispersal or adaptation in place, species face local extinction. Crucially, whether species rely on redistribution or adaptation in place has direct conservation implications. For example, if species are more likely to redistribute than adapt in place, conservation efforts focused on maintaining populations within current localities may be less valuable than strategies aimed at increasing habitat connectivity along redistribution corridors^8^.

Whether species adapt in place will depend in part on their relationship with temperature, which governs how climate change will translate to biological impacts. Temperature alters the rates and efficiencies of various internal processes, such as metabolism^9^, oxygen demand^10^, and hormone production^11^, therefore, changes to the thermal environment under climate change then scale up to detectable species responses, such as changes to the timing of life cycle events^12^, growth rates^13,14^, behaviour^15^, or distributions^16^. However, not all species are responding to climate change equally; some show increased vulnerability whereas others show some resilience or even benefit from these changes^17–19^. In particular, small ectothermic organisms such as insects are predicted to be particularly vulnerable^20,21^, as they are highly dependent on their environment to regulate their body temperature.

Thermal traits relate to the way in which a species interacts with temperature in its environment, such as thermoregulation or thermal tolerance. They are particularly important traits in ectotherm ecology, and have been used to explain large-scale patterns of species distributions^22^ and predict species responses to climate change^23^. These traits have direct consequences for species vulnerability to climate change. However, traits are not necessarily static over time. Evaluation of species vulnerability to climate change requires an understanding of exposure, sensitivity, and adaptive capacity^24^. Adaptive capacity refers to the ability of a species to cope with or adjust to climatic changes^25^, and is the most poorly understood of the three components^3^. Therefore, investigating thermal traits of species at one given space in time may not accurately reflect how those traits may respond to climate change. The adaptability of thermal traits will therefore be key in predicting species vulnerability.

Thermal traits can operate in several different ways, and represent different opportunities or challenges for species responses to climate change. One such trait is thermoregulation, which in ectotherms is largely the consequence of behaviour. Many ectotherms have adapted a suite of behaviours to alter patterns of heat loss or heat gain, such as microclimate selection within habitats^26^, basking^27^, or shivering^28^. Behaviour is often a highly plastic trait and so this thermal trait represents a potential rapid and initial response to environmental change^29^. However behavioural interactions with temperature rely on the thermal structure of their environment (e.g., microclimate availability), as such it may be constrained by habitat quality, which can be altered by human management. There is growing evidence that behavioural thermoregulation can inhibit selection for physiological thermal traits such as thermal tolerance (e.g., the Bogert effect)^30,31^. Therefore, we may expect to see stronger, more rapid, or more dynamic responses in behavioural thermal traits such as thermoregulation to the local climate than we would in physiological thermal traits, assuming equal habitat quality.

Thermal tolerance is another mechanism through which species could adapt to climate change, for example, by raising their upper thermal limit as air temperatures rise. Heat tolerance is the result of a complex combination of physiological and biochemical mechanisms^11^. In ectotherms, thermal tolerance can be variable within and among populations^32^, phylogenetically conserved^33–35^, and heritable, and therefore it should have adaptive capacity. Under thermally stressful conditions, organisms upregulate heat shock proteins which protect from heat damage by, for example, protecting or repairing heat damaged proteins^11,36^. Thermal tolerance would provide a more robust defence against non-tolerable temperatures, as behaviours have a limited capacity to avoid high body temperatures during extreme weather events, and in the case of ectotherm microclimate selection, depend on the availability and performance of those microclimates. However, as a trait that adapts across generations (though species do show some plasticity in this trait in the form of acclimation^9^), we may expect slower or more constrained responses in thermal tolerance to the local climate than we would in thermoregulation.

Functional traits such as body size and colour can influence thermal traits by altering rates of heat exchange with the environment. These relationships underpin ecogeographical theories such as Bergmann’s rule, the Temperature-Size rule, or the Thermal Melanism Hypothesis, though evidence in ectotherms remains mixed^37–39^. There may be limits to how much functional traits can vary in response to local climate due to their fitness consequences. For example, species colouration can be an adaptive response to predation pressure^40^, and so changes to colouration (such as paler colouration under warmer conditions) may increase predation and impose opposing selective pressure. Therefore, the functioning or flexibility of thermal traits and the functional traits that govern them will determine how species will respond to climate change.

Despite the importance of linking species traits to responses to climate change, direct evidence remains rare. A relatively commonly recorded response to recent climate change is changes in distribution along gradients, whereby many taxonomic groups have shown detectable elevational range shifts in response to recent climate change^16,41,42^. By combining measurements of thermal traits with long-term data on elevational range shifts from the same species in the same landscape^43^, we can directly assess how variation in thermal traits shape species realised responses to recent climate change. This will allow us to identify which traits influence species vulnerability to climate change, and gain relevant insights for conservation.

In the absence of long-term data to monitor change over time, space-for-time (SFT) substitutions are common as study systems to predict species responses to climate change^44,45^. Elevational gradients provide continuous changes in climatic conditions, while receiving similar weather, land-use pressures, and other covariates. Changes in thermal traits along an elevational gradient would indicate that populations respond to local climatic conditions, suggesting that such traits are flexible or could adapt to climate change. If thermal traits do not change across the gradient, this may indicate limited divergence in response to environmental variation, which could arise from constrains on adaptation, weak or inconsistent selection, phenotypic plasticity, or gene flow among populations. This lack of response could limit resilience to climate change, making populations more vulnerable, particularly for species with poor performance in their thermal traits.

Here, I focus on butterflies as a model group to investigate vulnerability to climate change. As insects they represent a large proportion of life on Earth^46^. Being small ectothermic organisms, they are sensitive to environmental temperature^47^, for example, by altering development rate^48^, reproduction^49^, behaviour^50^, and habitat associations^51^, and therefore butterflies are likely to show rapid and detectable responses to changes in environmental temperature. Butterfly thermal traits have been quantified in previous studies using well-established methods, however, to date only in singular locations, and comparisons have only been made across countries^52^ or even continents^53^, with no comparisons across continuous climatic gradients to date. As such, it is not yet known whether these traits respond to continuous changes in local environmental conditions, the role of functional traits in mediating this response, or whether thermal traits correspond to responses to recent climate change.

By investigating both thermoregulation and thermal tolerance in butterflies, I aim to capture complementary mechanisms of thermal responses to the local environment. Thermoregulation represents avoidance of temperature stress, whereas thermal tolerance represents enduring temperature stress. By investigating both and through what mechanisms they can vary, we can gain a better understanding of how species will cope with climate change, and reveal where flexibility or constraints may lie.

In this study, I aim to answer the following three research questions:

Do the thermal traits (thermoregulation and thermal tolerance) of butterfly species change across an elevational gradient, and is this impacted by species-level (species wing length, colouration) or individual-level traits (individual wing length, wing condition, sex)? I hypothesise that in cooler climates (higher elevations), butterflies should have thermoregulatory capacities that correspond to greater increases in body temperature across a narrower range of air temperatures (poorer thermoregulatory capacities) (to maximise activity within short periods of suitable weather) and have lower thermal tolerance (due to overall cooler conditions) compared to butterflies in hotter climates (lower elevations). I expect wing length and colouration to alter thermoregulation and thermal tolerance patterns as this has been shown in other butterfly systems, whereby small and dark butterflies heat up faster and reach higher body temperatures than large or pale butterflies, and also have higher thermal tolerance. I expect these patterns should also be reflected in individual-level traits, whereby within species, smaller individuals and individuals with better wing condition (and so brighter pigments, more complete patterning and lower scale loss) should also have poorer thermoregulator capacities and have higher thermal tolerance. As male and female butterflies can differ in size and colour^54,55^, I expect to see differences in thermoregulation and thermal tolerance between the sexes.

Do the functional traits (wing length, colouration) of butterflies change across an elevational gradient? I hypothesise that butterfly species in hotter climates (low elevation) should have an increased frequency of functional traits that correspond to improved thermoregulatory capacities (larger wing lengths both within and across species, paler colouration) compared to butterflies in colder climates (high elevation), due to the expected impact they would have on thermal traits, and in line with the converse-Bergmann’s rule and the Thermal Melanism Hypothesis.

Do thermal traits (thermoregulation) or functional traits (wing length, colouration) of butterflies alter their response to climate change (elevational range shifts)? I hypothesise that butterflies with a poorer ability to thermoregulate and with low flexibility in this trait will have shown stronger responses to recent climate change, and so greater upwards elevational range shifts, which would be consistent with climatic niche tracking. I hypothesise that the functional traits that should correspond to more dynamic exchanges of temperature with their environment and higher body temperatures (small and dark) should have stronger upwards elevational range shifts than species with more stable body temperatures, reflecting either increased sensitivity to warming or tracking of suitable climatic conditions.

## Results

Overall, 568 butterfly body temperature recordings were taken from 36 species, with an average of 63.1 butterflies sampled per site (Table S3). Air temperatures ranged from 11.5 to 29.8 °C, and body temperatures ranged from 16.4 to 37.1 °C (Supplementary Fig. 2). 277 butterflies had their thermal tolerance quantified from 15 species, with an average of 30.7 individuals sampled per site. CTmax values ranged from 44.0 to 47.1 °C (Supplementary Fig. 3). The average achieved ramp rate was 0.389 °C per minute, with ramp rates across all runs ranging from 0.338 to 0.449 °C per minute (Supplementary Fig. 4).

**Fig. 1:**
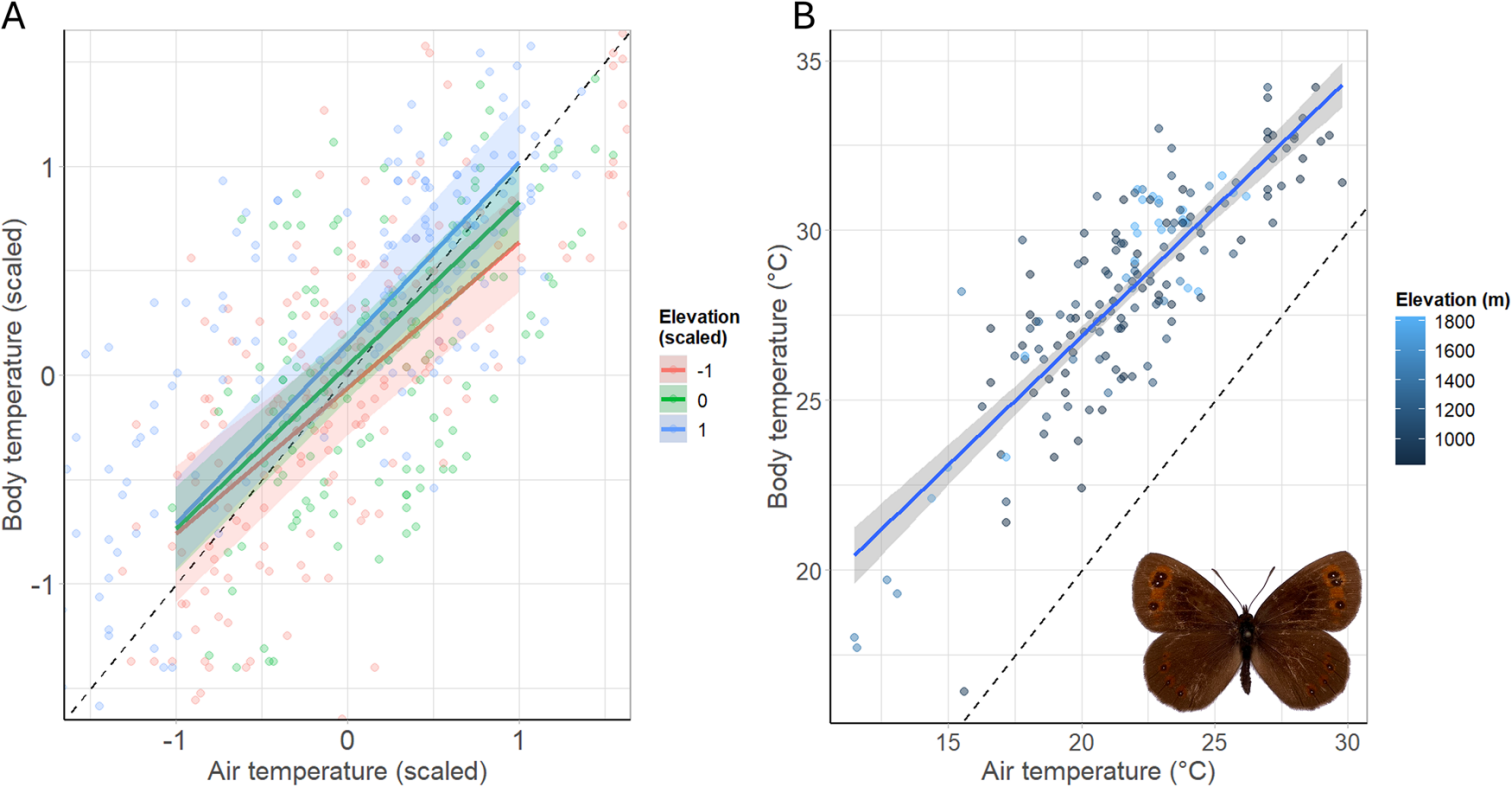
Body temperature responses to changes in air temperature in alpine butterflies across an elevational gradient. The relationship between body temperature and air temperature across the elevational gradient across all species (**A**). Note that the axes are scaled (so that unites represent standard deviations). An example is given of a single species (*Erebia aethiops*) (**B**) with unscaled axes. Points represent individual observations. Lines represent predicted responses while holding all other variables at their means. The coloured ribbons indicate 95% confidence intervals. The dashed lines represent a 1:1 relationship. Plots produced with the ‘ggeffects’ package^113^.

**Fig. 2:**
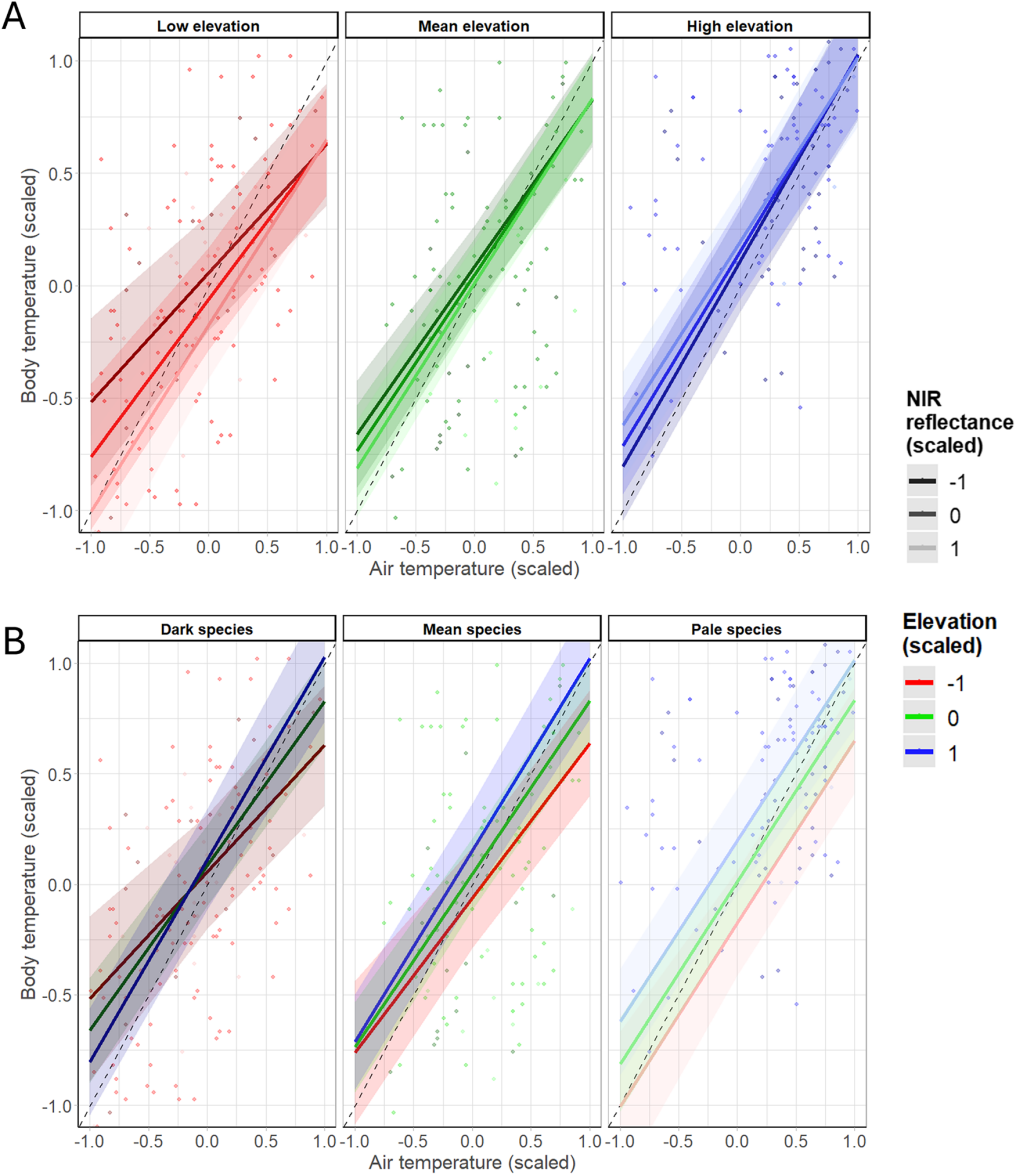
Body temperature responses to changes in air temperature in alpine butterfly species that differ in near-infrared reflectance across an elevational gradient. The effect of NIR reflectance (low values = dark species, high values = pale species) on the relationship between predicted body temperature and air temperature at different elevations. The result shown is the three-way interaction between air temperature, elevation, and colouration, shown in two complementary perspectives: (**A**) how elevation effects differ across colouration groups, and (**B**) how colouration effects differ across elevations. Note that all variables have been scaled (from −1 to 1) to ease comparisons (whereby units represent standard deviations with zero being the mean). Lines indicate predicted responses while holding all other variables at their means. Coloured ribbons indicate 95% confidence intervals. Points represent individual observations. In all plots colour indicates elevation (red = low elevation, green = medium elevation, blue = high elevation) and colour paleness indicates NIR reflectance (pale = high reflectance, medium = medium reflectance, dark = low reflectance). Plots produced with the ‘ggeffects’ package^113^.

Species differed in baseline body temperature across elevations (χ² = 6.093, d.f = 1, *p* = 0.014), whereby butterflies at higher elevations had higher body temperatures at any given air temperature (Table 1). However, the effect of this was relatively small, with butterfly body temperatures being 0.67 °C higher across the 1000 m elevational range. Thermoregulatory capacities were consistent across elevation (*p* = 0.805) (Fig. 1). Butterflies of poorer wing condition had significantly lower body temperatures (χ² = 11.921, d.f. = 4, *p* = 0.018), but worse thermoregulatory capacities (χ² = 14.503, d.f. = 4, p = 0.006) (Supplementary Fig. 5). Elevation altered the effect of colouration and species-mean wing length on the relationship between body temperature and air temperature (Colouration: χ² = 14.792, d.f. = 1, *p* < 0.001, Fig. 1A–B); species-mean wing length: χ² = 5.343, d.f. = 1, *p* = 0.021, Fig. 2A–B). The fixed effects explained 70.0% of the variation in body temperature, with the full model explaining 73.9%.

**Table 1:** Type II ANOVA tables for the linear mixed effects model of body temperature against air temperature, elevation, species-centred wing length, wing condition, sex, colouration, and species-mean wing length

Variable	χ²	D.f.	*P*-value	
**Air temperature**	**1014.371**	**1**	**<0.001**	***
**Elevation**	**6.093**	**1**	**0.014**	*
Species-centred wing length	0.005	1	0.946	
**Wing condition**	**11.921**	**4**	**0.018**	*
Sex	0.309	1	0.578	
Colouration	0.031	1	0.861	
Species-mean wing length	0.683	1	0.409	
Air temperature x Elevation	0.061	1	0.805	
Air temperature x Species-centred wing length	0.000	1	0.993	
**Air temperature x Wing condition**	**14.503**	**4**	**0.006**	**
Air temperature x Sex	0.679	1	0.410	
Air temperature x Colouration	1.598	1	0.206	
**Air temperature x Species-mean wing length**	**5.626**	**1**	**0.018**	*
Elevation x Species-centred wing length	0.058	1	0.809	
Elevation x Condition	2.541	4	0.637	
Elevation x Sex	0.632	1	0.427	
Elevation x Colouration	2.987	1	0.084	
Elevation x Species-mean wing length	1.831	1	0.176	
Air temperature x Elevation x Species-centred wing length	0.457	1	0.499	
Air temperature x Elevation x Wing condition	3.709	4	0.447	
Air temperature x Elevation x Sex	2.210	1	0.137	
**Air temperature x Elevation x Colouration**	**14.792**	**1**	**<0.001**	***
**Air temperature x Elevation x Species-mean wing length**	**5.343**	**1**	**0.021**	*

Interactions between variables is denoted with a ‘x’. Significant values are in bold and highlighted with asterisks (whereby *p < 0.05, **p < 0.01, ***p < 0.001).

At high elevation, dark and pale species performed similarly, with the differences in thermoregulation by colour becoming apparent only at low elevations (Fig. 2A). At low elevations, dark butterflies had improved thermoregulatory capacities but had consistently higher body temperatures than pale butterflies. Pale butterflies had consistent thermoregulatory capacities across the elevational gradient, with butterflies at high elevations having higher baseline body temperature (Fig. 2B). For dark butterflies, at high elevations butterflies had poorer thermoregulatory capacities, whereas at low elevations butterflies had improved thermoregulatory capacities, with higher body temperatures in low air temperature and lower body temperature in high air temperature.

**Fig. 3:**
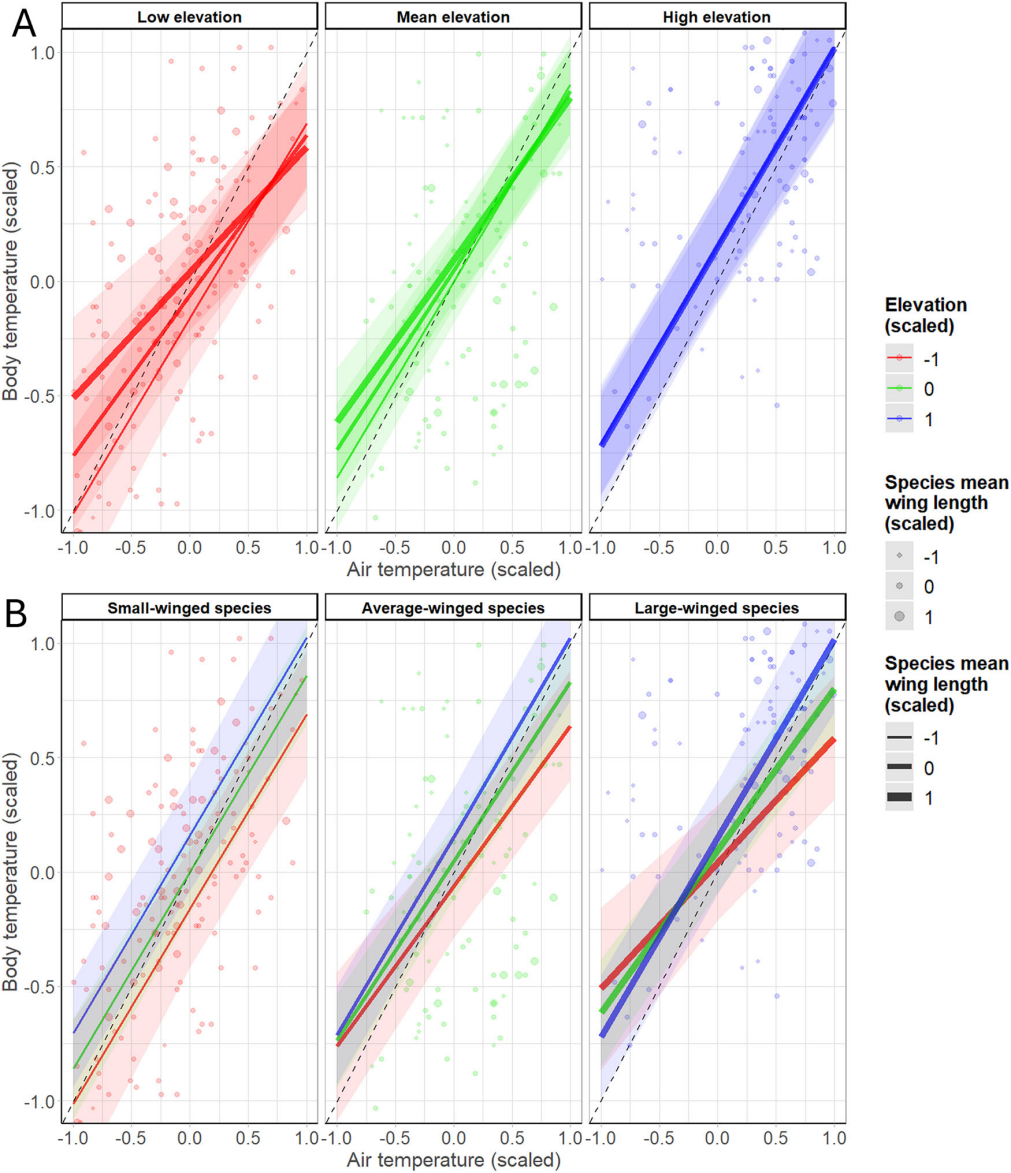
Body temperature responses to changes in air temperature in alpine butterfly species that differ in wing length across an elevational gradient. The effect of species mean wing length on the relationship between predicted body temperature and air temperature at different elevations. The result shown is the three-way interaction between air temperature, elevation, and species mean wing length, shown in two complementary perspectives: (**A**) how elevation effects differ across species mean wing length groups, and (**B**) how species mean wing length effects differ across elevations. Note that all variables have been scaled (from −1 to 1) to ease interpretation (whereby units represent standard deviations with zero being the mean). Lines indicate predicted responses while holding all other variables at their means. Coloured ribbons indicate 95% confidence intervals. Points represent individual observations. In all plots colour indicates elevation (red = low elevation, green = medium elevation, blue = high elevation) and line thickness and point size indicates species wing length (thin = small species, medium = medium species, thick = large species). Plots produced with the ‘ggeffects’ package^113^.

In the case of wing length, again at high elevations there were no differences between butterflies of different wing lengths, the differences only became apparent at low elevations (Fig. 3A). At low elevations, large butterflies had improved thermoregulatory capacities, and had higher body temperatures in low air temperature conditions, but lower body temperatures in high air temperature conditions, compared to small butterflies. Small butterflies had consistent thermoregulatory capacities across elevations, but small butterflies at high elevations had higher baseline body temperature than small butterflies at low elevations (Fig. 3B). For large butterflies, thermoregulatory capacities changed across elevations, with large butterflies at low elevations having stronger thermoregulatory capacities than large butterflies at high elevations, with marginally higher body temperatures in low air temperature and lower body temperature in high air temperature.

**Fig. 4:**
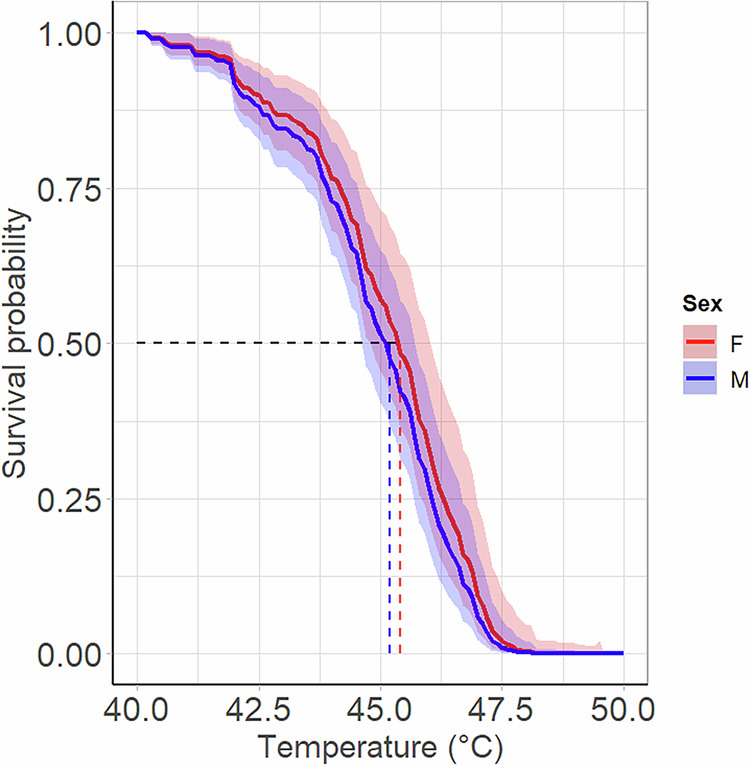
Differences in upper thermal limits between male and female alpine butterflies. Survival curves showing the effect of sex on survival probability. Dashed lines indicate LT50 (lethal temperature 50; the temperature at which 50% of individuals were knocked down), to ease comparison. Coloured ribbons indicate 95% confidence intervals. All plots produced with the ‘survminer’ package^100^.

**Fig. 5:**
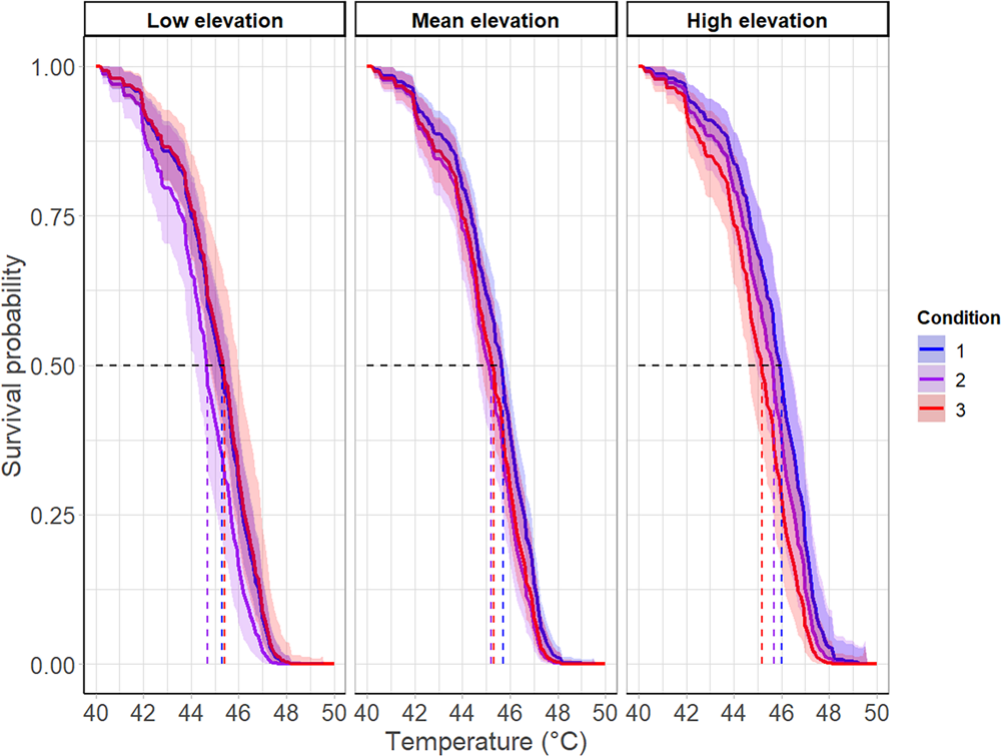
Differences in upper thermal limits of alpine butterflies of differing wing conditions across an elevational gradient. Survival curves showing the effect of wing condition on survival probability. Dashed lines indicate LT50 (lethal temperature 50; the temperature at which 50% of individuals h were knocked down), to ease comparison. Coloured ribbons indicate 95% confidence intervals. All plots produced with the ‘survminer’ package^100^.

There was no significant change in thermal tolerance across elevations (*p* = 0.749) (Table 2). Male butterflies had significantly lower critical thermal maxima than females (χ² = 5.678, d.f. = 1, *p* = 0.017) (Fig. 4). The impact of wing condition differed across elevations (χ² = 7.017, d.f. = 2, *p* = 0.030) (Fig. 5), whereby at lower elevations better condition individuals (fresher butterflies) had higher thermal tolerance, whereas at higher elevations, poorer condition individuals (older more damaged butterflies) had higher thermal tolerance. There was no significant effect of wing size (χ² = 1.480, d.f. = 1, *p* = 0.224) or elevation (χ² = 0.128, d.f. = 1, *p* = 0.720) on thermal tolerance.

**Table 2:** Type II ANOVA table for the mixed effects Cox model of knockdown temperature against elevation, sex, wing condition, colouration, species-centred wing length, and species-mean wing length

Variable	χ²	D.f.	P-value	
Elevation	0.102	1	0.749	
**Sex**	**4.443**	**1**	**0.035**	*
**Wing condition**	**6.826**	**2**	**0.033**	*
Colouration	0.059	1	0.808	
Species-centred wing length	0.098	1	0.754	
Species-mean wing length	0.957	1	0.328	
Elevation x Sex	2.861	1	0.091	
**Elevation x Wing condition**	**6.383**	**2**	**0.041**	*
Elevation x Colouration	0.614	1	0.433	
Elevation x Species-centred wing length	0.002	1	0.963	
Elevation x Species-mean wing length	0.000	1	0.987	

Interactions between variables is denoted with a ‘x’. Significant *p*-values are in bold and highlighted with asterisks (whereby *p < 0.05, **p < 0.01, ***p < 0.001).

**Fig. 6:**
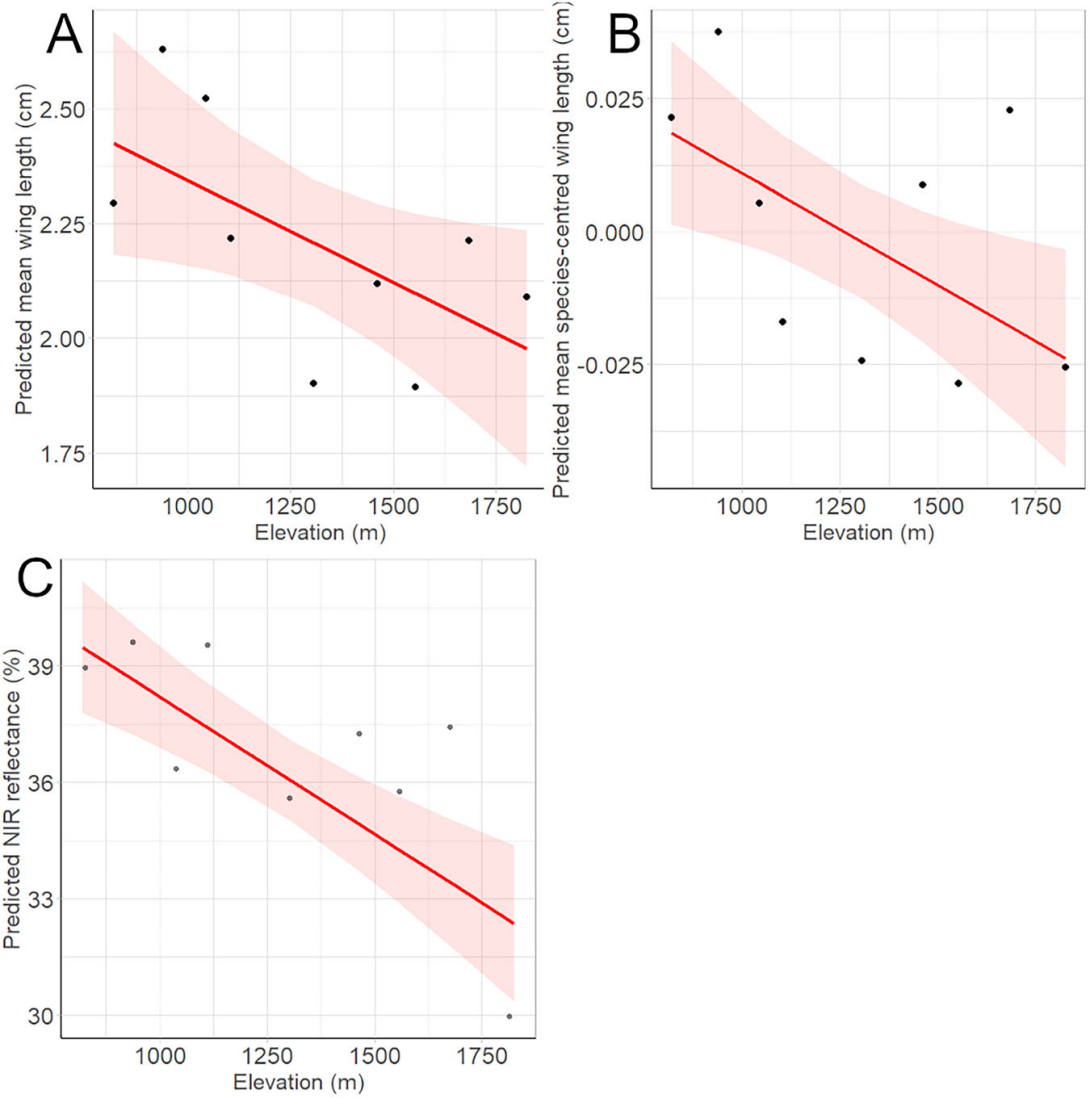
Responses of functional traits of alpine butterflies to an elevational gradient. The change in (**A**) mean wing length, (**B**) mean species-centred wing length (where values above zero indicate an individual above average size for its species, and below zero indicates an individual below average size for their species), and (**C**) mean near-infrared (NIR) reflectance across the elevational gradient. The points indicate mean values per site (to reflect the site random effect in the model), the red line shows the predicted response of the models. The coloured ribbons indicate 95% confidence intervals. For raw values see Supplementary Fig. 6.

There was a significant decrease in wing length with increasing elevation (χ² = 4.418, d.f. = 1, *p* = 0.036) (Fig. 6A, Supplementary Fig. 6A), equating to a 0.4 cm decrease in mean wing length across the 1000 m elevational gradient. There was a significant effect of elevation on species-centred wing length, where individuals within species were marginally smaller than average for their species at higher elevations, equating to 0.04 cm change in wing length relative to their species’ mean over the 1000 m elevational gradient (χ² = 0.117, d.f. = 1, *p* = 0.007) (Fig. 6B, Supplementary Fig. 6B). There was also a significant change in NIR reflectance across elevations (χ² = 6.773, d.f. = 1, *p* = 0.009), whereby butterflies were darker (lower NIR reflectance) at higher elevations (Fig. 6C, Supplementary Fig. 6C).

**Fig. 7:**
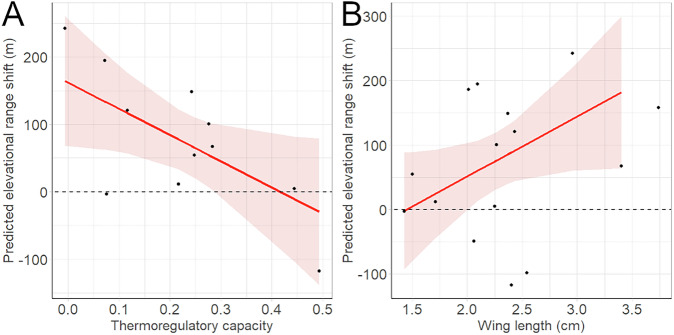
The impact of thermal and functional traits on changes in elevational ranges in alpine butterflies. The effect of (**A**) thermoregulation capacity and (**B**) mean wing length on changes in elevational ranges. Points represent species. The red line shows the predicted response. The coloured ribbon shows the 95% confidence intervals. Elevational shift values taken from Kerner et al. (2022).

There was a significant effect of thermoregulation capacity on elevational range shift, whereby species with a poorer ability to thermoregulate (lower thermoregulatory capacity) showed larger upwards elevational range shifts (χ² = 23713.3, d.f. = 1, *p* = 0.042) (Fig. 7A). There was also a marginal effect of wing length on elevational range shifts (χ² = 20463.6, d.f. = 1, *p* = 0.054) (Fig. 7B), whereby larger species had larger upwards elevational range shifts. There was no significant effect of colouration on elevational range shifts (*p* = 0.252).

## Discussion

Thermoregulation and thermal tolerance did not show intraspecific differentiation across the elevational gradient. Instead, thermoregulation was altered by species-level traits: colouration and wing length. At high elevations, butterflies of different sizes and colours performed similarly. However, with decreasing elevation, the importance of these traits increased, with dark and large species showing changes to their thermoregulation patterns. For thermal tolerance, there was no evidence of change across the elevational gradient, with the exception of wing condition. Butterflies differed in their thermal tolerance between the sexes, where males had consistently lower thermal tolerance than females across all elevations. Species-level functional traits of butterflies changed across the elevational gradient, whereby butterfly species were on average paler and larger at lower elevations. There was some evidence of intraspecific differences in wing length with elevation, with individuals within species being marginally larger with decreasing elevation. There was evidence of thermal and functional traits predicting responses to recent climate change, whereby species with poor thermoregulatory performance or large wing lengths showed greater upwards elevational range shifts.

Across all elevations, butterflies had relatively consistent thermoregulatory capacities, however, butterflies at higher elevations did exhibit marginally higher baseline body temperatures at any given air temperature. Butterflies thermoregulate primarily by the absorption and reflection of solar radiation^56^, and so the stronger solar radiation at higher elevations could explain this. The cooler air temperature at higher elevation may constrain the rate of heat gain. As the temperature differential between butterfly body temperature and air temperature increases, they lose heat faster through conduction, resulting in stable thermoregulatory capacities across climates. Climate change is predicted to alter cloud cover patterns^57,58^, which may have implications for butterfly thermoregulation and warrants further investigation. As I detected no evidence of change within species, this implies that thermoregulation patterns are relatively conserved across the elevational gradient, and may not respond to local climatic conditions. Therefore, species with poor thermoregulation abilities may be particularly vulnerable to changing environmental conditions. It is important to consider, however, that results may differ across larger elevational gradients, across a larger pool of species, or with larger sample sizes. Previous data from the European Alps shows temperature change with elevation ranging from 9.8 to 4°C over 1000 m (depending on air dryness)^59^. This temperature range may not be sufficient to elicit intraspecific responses in thermal traits. As such it would be valuable to investigate larger gradients that encompass a wider range of temperatures. Nevertheless, the gradient included in this study represents meaningful climatic variation relevant to the thermal ecology of the study species (particularly as small ectotherms), and the significant interspecific effects of elevation detected suggest that even this range was sufficient to capture biologically relevant temperature differences. Furthermore, intraspecific trends may be more detectable with more intense sampling or in other taxonomic groups, we caution that we cannot exclude the possibility of intraspecific adaptation in thermal traits in other species or systems, and instead highlight that in this case, there is insufficient evidence that butterflies alter their thermoregulatory capacity in response to their local climate. Selection for improved behavioural thermoregulation may have fitness consequences outside of non-tolerable temperatures (for example, spending more time avoiding heat instead of feeding or locating mates), and so natural selection may impose conflicting selective pressure against increased thermoregulatory behaviours. Alternatively, behavioural thermoregulation relies on landscape structure, for example, the presence of suitable microclimates. Should those microclimates be lost or competition for them increase, this may inhibit changes to behavioural thermoregulation.

I also found no evidence of the thermal tolerance of butterflies responding to local climatic conditions. Upper thermal limits are likely to be particularly important for coping with extreme temperature conditions rather than gradual changes to mean temperatures. In Europe, heatwaves are increasingly common, with temperatures in Germany reaching nearly 40°C, close to the upper thermal limits of the species tested (and in some parts of Europe reaching nearly 50°C)^60^. Considering that to date, studies that have reported butterfly body temperatures show limited evidence that they can lower their body temperatures below ambient in hot weather and instead are likely to thermoconform^52,61,62^, these increasing heatwaves present a challenge for species persistence in the absence of adaptation. Adaptation to cope with extreme temperatures may be inhibited by their transient nature or inherent scarcity, particularly for taxa such as butterflies with short generation times. Therefore, selection imposed by infrequent extreme temperature events can be reverted back to phenotypes that cope better with averages than extremes in subsequent generations^63^. It would be of value for future research to determine whether thermal tolerance patterns change over time in response to recently experienced extreme weather events. This result is consistent with the literature, where patterns in thermal tolerance (particularly upper thermal limits) across elevations and latitudes are weak, complex, and often inconsistent^64,65^. It is worth considering that adult butterflies are highly mobile and so it is possible that the movement of adults across elevations may obscure any detectable patterns in thermal tolerance, and gene flow may further erode local adaptation. As such, future research should consider more sessile life stages, such as larvae.

Across all elevations, male butterflies had consistently lower thermal tolerance than female butterflies. This is likely the result of protandry in butterflies^66,67^. This is where males eclose before females, and so can experience different climatic conditions, particularly in highly seasonal climates such as the study region. Female butterflies, emerging later in the season, are more likely to experience higher temperatures, and so it is possible that their higher thermal tolerance reflects adaptation to cope with this. The consequence of this is sex-specific vulnerability to extreme temperatures, where it is possible that males may be more vulnerable to extreme temperatures than females, though emerging earlier means that it is also possible that they are less likely to experience these extreme temperatures.

Condition also affected thermal tolerance, and the impact of this changed across the elevational gradient. In general, the differences in condition became more extreme at high elevations, whereby individuals of lower condition (older or more damaged individuals) had lower thermal tolerance than fresher more complete individuals. This may reflect accumulated damage reducing overall performance, or the loss of high temperature resistance required in the sessile pupal stage (as seen in other holometabolous insects^68^). Similar loss of heat resistance with senescence has been shown in other insects^69,70^, but to our knowledge this is the first time this has been reported in butterflies. Our results suggest that the rate of loss of heat resistance with senescence in butterflies is climate dependent.

As thermoregulatory capacity showed limited intraspecific variation across the gradient, the pattern of stronger thermoregulatory capacity at lower elevations appears to be driven by species turnover within functional groups, specifically dark and large species with poor thermoregulatory capacity appearing to be replaced at lower elevations by other dark and large species with greater thermoregulatory capacities, suggesting that within these functional groups, species sorting via thermoregulation capacity rather than within-species adaptation may be a dominant response to thermal conditions across the gradient. In this study, ‘colouration’ refers to the absorption (dark) or reflection (pale) of NIR wavelengths in the basal wings, an adaptation to directly increase the temperature of the flight muscles and should correspond only to a thermoregulatory function (as this is a non-visible part of the spectrum and so will not play a role in sexual selection, protection from UV damage, camouflage, or display). It would be valuable for future research to quantify individual NIR reflectance across elevational gradients to determine whether this trait shows adaptive potential, however, previous studies have shown that NIR reflectance shows little intraspecific variation^71^.

At higher elevations, dark and pale butterfly species had similar thermoregulation capacities and body temperatures. This may reflect the higher solar radiation conditions at high elevations allowing butterflies of all colours to achieve similar body temperatures. Or alternatively the higher solar radiation at high elevation may increase rate of heat gain, but the cooler air temperature would increase the rate of heat loss, resulting in stable performances across colourations. The impact of colouration only becomes apparent under hotter climatic conditions, where thermoregulatory capacity changes in dark butterflies. Pale species did not show this response, and had stable thermoregulatory performance across various climates, which may explain why species such as *Pieris rapae*, which has one of the highest NIR reflective values in our study species, has become a successful globally invasive agricultural pest^72^, as it is able to thermoregulate efficiently across various climatic conditions. In contrast, the thermoregulatory capacities of dark butterflies changed with climatic conditions, where dark butterflies had improved thermoregulatory capacities but ultimately always had higher body temperatures than pale butterflies at low elevations. This could be the result of decreased temperature differential between the butterflies’ body temperature and the warmer air at lower elevations, which would reduce the rate of heat loss, whereas the rate of heat gain would be relatively stable. This would make it particularly challenging for dark species to reduce their body temperature, particularly in open sunny habitats such as the grasslands surveyed in this study. Selection would then favour dark species in hotter climates with adaptations to avoid overheating. We can see evidence for this in the improved thermoregulatory capacities of dark species at lower elevations. However, the consistently higher body temperatures of dark species compared to pale species means that there appears to be limitations on the behavioural responses that dark species can use to reduce their body temperature in hot weather. This means that even dark species with improved thermoregulatory capacities are still more at risk of overheating compared to pale species. Therefore, we may expect initial species replacement and ultimately loss of dark species with increasing temperatures. I detected a shift from dark to pale species with decreasing elevation, and given the association between colouration, thermoregulatory capacity, and elevational range shifts identified in this study, this pattern is consistent with thermal sorting of communities by wing colouration. We should therefore expect dark species with poor thermoregulatory abilities to be at particular risk under future climate change. An example of this would be *Erebia manto*, which had one of the highest NIR absorption values but also one of the weakest thermoregulatory capacities. This species had also shown one of the strongest responses to recent climate change, in one of the largest upwards elevational shifts.

The trend in colouration across the elevational gradient supports the thermal melanism hypothesis, whereby species tend to be darker in colder climates^37^. Gradients in butterfly colouration have been detected over large geographic areas^71,73^, but rarely across an elevational gradient^74–76^. I did not explicitly quantify melanism, instead used a measure of NIR reflection in the basal portion of the wing. As lower NIR reflectance (and so higher absorption) in colder environments would increase solar absorption and so the rate of heat gain, this parallels the expectation that darker species (higher absorption in the visible spectrum) are favoured in colder climates. Our results suggest that this pattern may not only be the result of dark species having increasing thermoregulatory capacities in cold environments (as butterflies of all colours performed similarly at high elevations), but rather also the increased risk of dark species overheating in hot climates resulting in increasing prevalence of paleness with increasing climatic temperature.

Similarly to colouration, butterfly wing length also altered thermoregulatory performance across the elevational gradient. At high elevations, butterflies of all sizes had similar thermoregulatory capacities. Again, this could be due to the high solar radiation and low air temperature conditions found at high elevations resulting in stable patterns across sizes. The impact of size on thermoregulation only became apparent at lower elevations, and only for large species. Small species showed consistent thermoregulatory capacities across the gradient. Their small size would result in a more dynamic exchange in temperature with their environment due to their higher surface area to volume ratio^77^, which would limit their capacity to avoid high body temperatures. Their small size would also limit their dispersal ability^78^, which would reduce their ability to access microclimates to avoid high heat over large areas or distances, or that distances between suitable microclimates take longer to move between, which coupled with their more dynamic thermal exchange with their environment, may reduce their ability to avoid high body temperatures. The consistent thermoregulatory capacity of small butterflies across the elevational gradient, combined with their reduced representation at lower elevations, suggests that small species may have a limited ability to adjust their thermoregulatory capacity in response to local climatic conditions, with potential implications for their vulnerability to future warming.

In contrast to small species, large species showed a more dynamic response across the gradient. As thermoregulatory capacity showed no intraspecific variation across the gradient, the pattern of stronger thermoregulatory capacity in large species at lower elevations appears to be driven by species sorting within this functional group according to their ability to thermoregulate, whereby large species with a greater ability to maintain stable body temperatures under warmer conditions are disproportionately represented at lower elevations. This replacement may be the consequence of increased sensitivity of large species to extreme heat, however their greater dispersal ability and flight performance means they may be more capable of behaviourally responding and showing community-level responses across the gradient, unlike small species which are more thermodynamically constrained. For example, there is evidence of links between large body size and lower thermal tolerance in some insect groups^79^, including butterflies^80^, however, this was not detected in our study system, though this could be due to our smaller number of species tested than other studies. This trend could be due to unsustainable metabolism or oxygen demand in larger bodied insects relative to small-bodied insects under high temperature conditions^11^, and so large species may experience stronger selective pressure to behaviourally avoid excessive heat. Alternatively, large species should have greater dispersal ability^78^, and would therefore be able to access microclimates faster and over a wider area than small species at any given elevation. This may be particularly relevant in this case, as all study sites were open grasslands with little shade other than the surrounding forest. Large species would have less dynamic body temperatures than small species^77^, and so with behavioural responses combined with larger wings to improve their flight performance, they would be able to maintain cooler body temperatures by moving between nearby forest and the grasslands more rapidly than small species could, and spend more time in the grassland before their body temperature reaches non-tolerable levels and they must seek shade. The consequence of this difference is that small species may have a reduced capacity to avoid high body temperatures, particularly in open habitats such as grasslands, and therefore are more likely to be lost under future climate change and replaced by large species. We can see evidence for this in our study system, with the increased representation of large species with decreasing elevation.

Patterns in Lepidopteran wing length across elevational gradients to date have shown inconsistent results^81–84^. Our results support the converse Bergmann’s rule at both the intraspecific and interspecific levels, whereby species and individuals within species were smaller at higher elevations. This is often predicted to be due to the shorter season length in cold climates, and so growth, foraging, and development are limited and consequently so is body size. Our findings highlight that thermal traits may also be influencing these patterns of body size, whereby species are also limited by overheating risk in hot climates, resulting in larger body sizes that are better able to avoid excessive body temperatures. Despite aligning with the converse Bergmann’s rule, this finding goes against the Temperature-Size rule, whereby in Lepidoptera higher temperatures experienced during development result in smaller adult body sizes^85^. In this case, as the focal taxa are highly mobile adult butterflies, the location of the adults may not necessarily reflect the location of the larvae, and therefore the temperatures experienced during development. It would be valuable to rear caged larvae at different elevations and collect the eclosed adults, as these would provide stronger evidence for or against the Temperature-Size rule.

The change in the distribution of species-level functional traits across the elevational gradient implied a redistribution of species rather than adaptation in place, where species are replaced by more resilient species, or lost. At lower elevations where conditions are hotter, butterfly communities tended to contain more species with functional traits that corresponded to more stable body temperatures and in particular, avoidance of high body temperatures in hot weather. This implies adaptation to avoid overheating; rapid heat gain at high elevations may impose negligible risk as air temperatures tend to be cooler and non-tolerable body temperatures are unlikely to be reached. However, at lower elevations this risk increases, and so selection would favour functional traits that reduce the rate of heat gain in hot weather. However, there was evidence of intraspecific variation in wing length across the gradient, with individuals at lower elevations having larger wings for their species compared to their high elevation conspecifics. A similar pattern has been documented in some butterfly species^84,86^, and implies some adaptive potential in relation to size, though the shift in size detected was very small (equating to an increase in wing length of 0.4 mm from the species mean across the 1000 m elevational gradient). The small magnitude of this change may be the consequence of species-specific responses being pooled together, as responses in direction and magnitude have differed between species in previous studies^84^. It is possible that shifts in functional traits may have impacts on the dynamics of their ecosystem. For example, larger butterflies typically have longer larval development time^87^. This would increase the window of exposure of vulnerable larvae to sources of mortality: predators and disease (the slow-growth-high-mortality hypothesis)^88^, and under climate change, an increased likelihood of experiencing extreme weather events. Therefore, we may expect fewer butterflies to survive to adulthood should a community shift towards a higher proportion of large species. Similarly, size in butterflies is correlated with voltinism^89^, with larger species having fewer generations per year. This shift would reduce the rate of recovery of butterfly communities following disturbance, such as an extreme heat event. As evolution can only occur across generations, this reduction in generations per year would further reduce the overall adaptive potential of butterfly communities.

I found that thermoregulatory capacity and wing length influenced species responses to recent climate change. Species with poorer thermoregulatory performance had greater upwards elevational range shifts over a recent 10-year period (2009-2019). This suggests that though thermoregulatory performance does not vary across space, it is linked to species distributional responses to climate change, consistent with species tracking suitable climatic conditions through range shifts rather than through local differentiation. This highlights the importance of considering thermal traits when predicting species responses to climate change. An unresolved question is the direction of causality between the range shifts and local adaptation. One possibility is that species with poor thermoregulatory capacities and an inability to locally adapt may experience reduced performance under changing climatic conditions, and therefore redistribute to track suitable conditions. Alternatively, the range shifts themselves may reduce exposure to novel thermal conditions, therefore limiting the opportunity or necessity for local adaptation. These processes are not mutually exclusive and may result in similar macroecological patterns. It may be possible to use thermoregulatory capacity, as a fixed trait in butterflies with, to date, no evidence of change within species across various spatial scales^52,53^, as a measure of vulnerability and range shift potential in response to climate change. Within alpine communities, it would be valuable to identify species already occupying high elevations with poor thermoregulatory capacities (in this study, such as *Erebia manto* or *Erebia ligea*, with thermoregulation capacities of 0.07 and 0.12, respectively), as these species may be expected to have particularly strong upwards elevational range shifts (194.9 m and 121.1 m, respectively), and so be particularly vulnerable to climate change.

Our results imply that montane butterfly conservation should prioritise well connected areas of suitable habitat to enable the movement of species, with habitat corridors likely to become increasingly important for species persistence. Small species in these systems may disproportionately benefit from increased habitat connectivity, particularly in the study region where grasslands are often isolated with large stretches of dense forest between them that may be acting as non-permeable barriers to redistribution. Translocations, such as between isolated mountain ranges or highly isolated grasslands may be necessary in cases where habitat cannot be connected. Conservation planning should anticipate the redistribution of species, and potential disproportionate losses of small and dark species. Attention should be given towards novel or disrupted ecological interactions (such as myrmecophilous lycaenid species) and trophic mismatches (such as larval foodplant availability) which may threaten species persistence after redistribution. Incorporating climate and trait data, particularly thermal traits, can improve predictions of where species redistributions are likely to occur. Range-restricted species in particular, such as poor dispersers or montane specialists, will face habitat loss and higher extinction risks if they cannot redistribute.

In this study, an elevational gradient was treated as a space-for-time (SFT) substitution, however, this approach may not accurately reflect future dynamics should unconsidered covariates, non-linear responses, or acclimation occur^90^. There is some evidence that, with caution, SFT substitutions can provide valid insights into microevolutionary processes^44^. In this case, the spatial distribution of study sites was very narrow, with all sites within 20 km of each other within a continuous landscape, to date this is the narrowest range of compared sites for butterfly thermal traits, which so far has occurred between countries^52^ and continents^53^, though the results across all studies show consistent trends across spatial scales. It has been proposed that SFT substitutions can be reliable if there is a strong causal relationship between the variable being tested and the climate^90^. Here, I argue that thermal traits are particularly suited to this purpose, as they directly relate to and are the consequence of the way a species interacts with temperature in its environment. Further, the timescale over which species have occurred and adapted within these areas compared to the rate of climate change should somewhat overestimate species adaptive capacity rather than underestimate, however, in this case, I found limited evidence of intraspecific adaptation occurring at all.

This study provides a trait-based perspective on species redistributions under climate change by linking thermal ecology and macroecological responses. Our results indicate that the thermal traits of butterflies show limited intraspecific variation across an elevational gradient. As a result, species persistence in these systems is likely to rely on redistribution rather than adaptation in place. We should expect that butterfly assemblages will differ in functional traits as the climate changes, with disproportionate losses of small and dark species compared to large and pale species in the Alps. As I detected strong responses of poor thermoregulators to recent climate change in the form of greater upwards elevational range shifts, it is imperative that these species are identified and receive conservation efforts to protect them and their habitats before they are lost.

## Methods

### Study sites

**Fig. 8:**
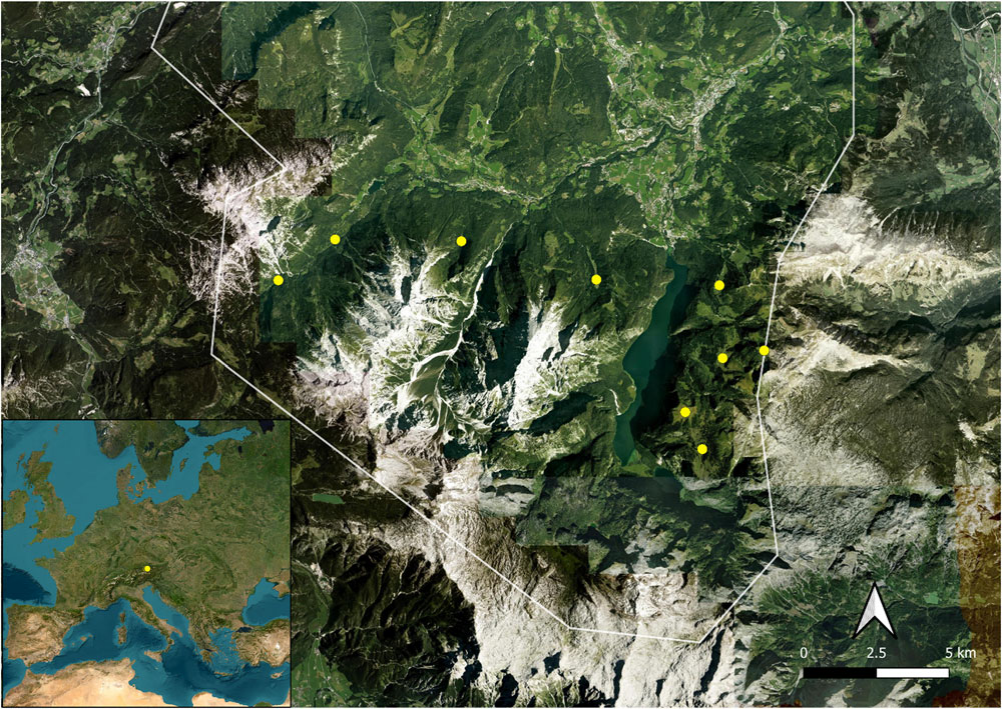
A map of the study system, Berchtesgaden National Park, a region of the Alps in southern Germany. A map of the sample sites within Berchtesgaden National Park in Bavaria, Germany, with an inset continental map of Europe. The white line indicates the border with Austria. Yellow points represent the sampling locations. Base layer ESRI Satellite imagery, accessed via QuickMapServices plugin in QGIS 3.42.2. This includes imagery from Esri, DigitalGlobe, GeoEye, i-cubed, USDA FSA, USGS, AEX, Getmapping, Aerogrid, IGN, IGP, swisstopo, and the GIS User Community.

Nine sites were selected for sampling in Berchtesgaden National Park in southern Bavaria, Germany (Fig. 8). Berchtesgaden National Park is composed of four mountain chains ranging from 600 m to 2700 m above sea level, and covers an area of 210 km^2^ ^91^. The nine sites were selected based on structural similarity (open grasslands of comparable sizes containing high floral diversity and grazed by cattle in the summer months, and so of comparable habitat quality) and spanned an elevational gradient of 820 m to 1825 m (Table S1). All sites were within a 20 km area. Sites were sampled from 5^th^ to 27^th^ August 2025. During each visit, butterflies were sampled using a systematic area search method, in which the observer walked continuously through each sampling area ensuring all parts of the site were approached within 10 m. Sites were visited in a random order, and sampling took place for a standardised total duration of six hours, divided into three two hour sessions (three total samples per site) across the day to ensure sampling captured variation in butterfly activity across different weather and temperature conditions (Table S2). Sites were only visited in suitable weather, that being not raining or high wind speed conditions (above five on the Beaufort scale).

### Quantifying thermoregulation

To quantify thermoregulation, once a butterfly was caught in the net it was immediately immobilised (by pulling the net taut around it, without touching it with hands which may artificially alter their body temperature) and moved into the shade (shadow of the recorder). A precise thermocouple (Tecpel Thermometer 305 P with 0.5 mm diameter mineral insulated thermocouple, Tecpel Co. Ltd.) was then pushed through the net and pressed against the external lateral surface of the thorax to record their body temperature. Body temperature recordings were taken within 10 seconds of capture, otherwise the data was discarded. Once the body temperature was recorded, air temperature was then recorded at a standardised height (waist height of the recorder in their own shadow) using the same thermocouple. While in the net, wing length was recorded from the base of the forewing to the furthest apex using callipers. Wing length was chosen as a proxy for body size and dispersal potential, as it is correlated with both body mass^92^ and dispersal ability^78^ in butterflies, and can be recorded quickly and without damage to the insect. Wing condition was recorded on a scale from 1 to 5 (whereby 1 is perfect condition with no scale loss, 2 is minor scale loss, three is heavy scale loss and/or minor damage to the periphery of the wings, 4 is more substantial damage to the wings no longer restricted to the very outer edges, and 5 is heavy damage to all wings). Sex of each individual was determined by sexual characteristics (checking the terminalia in cases without sexual dimorphism), and all individuals were identified to the species level (or species complexes in cases of complex or unclear taxonomy). The butterfly was then marked (to prevent recapture) and released, except for a subset of highly abundant species that were retained to quantify thermal tolerance.

### Quantifying thermal tolerance

Upper thermal limit (CTmax) was quantified using a heat knockdown assay and ramping temperature protocol. Butterflies were stored in glassine envelopes from the field until the experiment for no more than four hours, and kept at ambient temperature in shade. Only butterflies of conditions 1-3 were retained, in order to reduce the risk of senescence or poor condition impacting the results. Butterflies were randomly placed individually into one of five jars in a water bath, with 2 ml of water on filter paper at the base to maintain high humidity throughout the experiment. During the experiment, the observer was blinded to the site of origin or each individual. The starting temperature was 25 °C, which reflected the average air temperature recorded during previous sampling in the same region (June–July 2025). After being placed in the jars, butterflies were allowed to settle for 5 min before the experiment began. After 5 min, the water bath was set to ramp up in temperature by 0.5 °C per minute for 90 min (Supplementary Fig. 1). This ramping rate was selected to replicate ecologically relevant changes in temperature that may be experienced under natural conditions, but fast enough to prevent prolonged stress or other intrinsic factors affecting the results of the experiment^93,94^. To monitor the ramping rate of the water bath, a thermocouple was placed into a control jar to record internal air temperature, which was recorded every five minutes. This both ensured that the ramping rate was similar across experiments, and also allowed for the confirmation of the actual achieved ramp rate that the butterflies would experience inside the jars (as the air temperature in the jars would lag behind the temperature of the water itself). Butterflies were monitored inside the jars until they lost motor control. This was defined as when they lose the ability to do coordinated movement. Butterflies would be poked with a piece of wire through the lid of the jar, and if they did not respond using coordinated movement (such as walking, flying, or standing back up if knocked down, i.e., they had no righting response), they were considered ‘knocked down’ and they were removed from the experiment. Immediately after being knocked down the butterflies were removed the water bath and allowed to recover in a recovery cage, after which they were marked and released back to their original locations.

### Statistics and reproducibility

All analyses took place in R version 4.5.1 (R Team, 2024). In all cases, model assumptions were checked using the ‘performance’ package^95^ or the ‘lmerTest’ package^96^ for mixed effects models, and the ‘survival’ package^97^ for survival curve analysis prior to fitting, and term significance was assessed with the ‘car’ package^98^. Plots were produced with the ‘ggplot2’ package^99^ or in the case of survival curves, the ‘survminer’ package was used^100^. Before analysing the thermal survival curves, differences in ramp rate were checked using a linear regression with the temperature as the response variable, and the time and run identity as the explanatory variables, with an interaction between the two. The achieved ramp rate was calculated as the slope between temperature and time. The effect of ramp rate on CTmax values was tested with a linear mixed effects model with knockdown temperature as the response variable and ramp rate as the explanatory variable. Species was included as a random effect to account for non-independence of the data points. Results of these checks can be found in Supplementary Materials Results 1.

In order to test whether thermoregulation changed across the elevational gradient, a linear mixed effects model was fit with the ‘lme4’ package^101^ (*n* = 568). The response variable was body temperature, and the predictor variables were air temperature, elevation, wing length (centred within species), species-mean wing length, species-level colouration, wing condition, and sex. Colouration was taken from^102^ whereby reflectance values were taken from the basal region of the wings in the near-infrared (NIR) range (hereafter referred to as colouration for simplicity), from either the dorsal surface if the species was a dorsal basker, or the ventral surface if the species was a lateral basker (basking type taken from^103^). This was selected as temperatures in this region directly affect the temperature of the flight muscles^73,104^ with regions beyond the basal part of the wing having less of an impact on thermoregulation^105^. Therefore, this is a region that is likely to experience particularly strong selection and provide clear and detectable signals of change. Near-infrared was selected as approximately 50% of solar radiation is composed of wavelengths from 700 to 1400 nm, but as it is not in the visible spectrum it does not affect camouflage or display, meaning NIR absorption is particularly relevant to thermoregulation in butterflies, and has been previously shown to have adaptive correlations with climatic factors at large geographic scales^71,73^. Interactions were included between all traits and air temperature and elevation up to three-way interactions. Species and site were included as random effects to account for repeated sampling and non-independence. Due to the complexity of the model and the various scales and units used, all variables were scaled prior to fitting. Random effect performance was checked with the ‘lmerTest’ package^96^. To determine whether phylogenetic relatedness among species influenced their body temperature, I fit two Bayesian mixed models using the ‘MCMCglmm’ package^106^; one incorporating a phylogenetic covariance structure based on a phylogenetic tree from^107^, and an otherwise identical model without this structure. Model fit was compared using the Deviance Information Criterion (DIC). The two models performed nearly identically (ΔDIC = -0.08), indicating no meaningful improvement (and marginally poorer performance) when including phylogeny. As such, subsequent analyses and reported statistics are from the non-phylogenetic model. As an additional check for phylogenetic signal, I tested whether species-level random effects extracted from the linear mixed effects model were structured by evolutionary relatedness using Pagel’s lambda from the ‘phytools’ package^108^. A lambda value of zero (*p* = 1) indicated no phylogenetic signal, meaning that closely related species were no more similar in their body temperatures than would be expected by chance. To check whether the results were robust to the inclusion of species with few observations (rare species), I fit the model on two datasets; the full dataset and a reduced dataset excluding species with fewer than 10 observations. Model coefficients were compared between the two fits to assess consistency in their direction and strength. To further validate this comparison, I used a 10-fold cross-validation to assess whether rare species were influencing model predictions. In each fold, both models were trained on 90% of the data, and tested on the remaining 10%. To disentangle the effect of rare species from the effect of sample size, I additionally trained an additional model on a random subsample restricted to only common species but equal in sample size to the full dataset. Predictive performance was compared using root mean squared error across folds. The full model (mean RSME = 0.503) and the matched model (mean RSME = 0.508) performed similarly, and both outperformed the subset model (mean RSME = 0.574), indicating that the difference in performance was attributable to sample size rather than the inclusion of rare species. Consistency of fixed effects across the full and reduced models further supported the robustness of results to the inclusion of rarely observed species. Thermoregulation could then be compared between groups by considering changes in intercept (baseline body temperature) and slope (the relationship between body temperature and air temperature, or ’thermoregulatory capacity’). Term significance was tested using Type II ANOVA tests.

To test whether thermal tolerance changed across the elevational gradient, a mixed effects Cox model was fit using the ‘coxme’ package^109^ on species with a minimum of five data points (n = 295). This used the knockdown temperature as the response variable, with elevation, species-centred wing length, species-mean wing length, species-level colouration, sex, and wing condition as predictor variables, as well as interactions between all variables and elevation. Species and site were included as random effects. Again, to test whether there was a phylogenetic signal in the survival analysis, I fit a phylogenetic Cox mixed effects model incorporating species-level phylogenetic covariance derived from^107^. This was compared against an equivalent non-phylogenetic model with a standard species random effect. AIC comparisons showed that the phylogenetic model did not outperform the non-phylogenetic model (ΔAIC = 1.91), again indicating no meaningful phylogenetic signal. As such, subsequent analyses and reported statistics are from the non-phylogenetic model. As an additional check for phylogenetic signal, I tested whether species-level random effects extracted from the Cox mixed effects model were structured by evolutionary relatedness using Pagel’s lambda from the ‘phytools’ package^108^. A lambda value of zero (*p* = 1) indicated that no phylogenetic signal, meaning that closely related species were no more similar in their upper thermal limits than would be expected by chance. To check whether the results were robust to the inclusion of species with few observations, I fit the Cox model on two datasets; the full dataset and a reduced dataset excluding species with fewer than 10 observations. I then compared model coefficients for consistency in direction and strength. To further validate this comparison, I used 10-fold cross-validation to assess whether the rare species were influencing the model predictions. In each fold, models were trained on 90% of the data and tested on the remaining 10%. Predictive performance was assessed using the concordance index (C-statistic). Both models performed similarly (difference in C-statistic = 0.002), and fixed effects were consistent in strength and direction, supporting the robustness of the results to the inclusion of rarely observed species. Model assumptions were checked with the ‘survival’ package^109^. Term significance was again tested using Type II tests.

To determine whether the functional traits of butterfly species (wing length and colouration) changed across the elevational gradient, three linear mixed effects models were fit, with species-mean wing length, species-centred wing length, and colouration as response variables, and elevation as the predictor variable. For the model of wing length, site was included as a random effect. This random effect was excluded from the colouration and species-centred wing length models as it did not significantly improve the models (assessed with ‘lmerTest’ package and AIC scores). Again, model assumptions and random effects were checked, and term significance was tested using Type II tests.

In order to test whether thermal traits influence the response of butterflies to recent climate change, species elevational shifts over a 10-year time period in the same mountain range was taken from^43,110^, where species-specific elevational range shifts were extracted^110^. I then fit a linear regression model with elevational range shift as the response variable, and the inverse of the slope between body temperature and air temperature per species as a measure of thermoregulation capacity (inverted so that high values indicate a stronger ability to thermoregulate compared to low values, as in similar studies^61,80,111^. If a significant change in thermoregulation was found across the elevational gradient, the standard deviation in slope values was included as a measure of trait flexibility. Wing length and colouration were also included as explanatory variables to determine whether functional traits influenced elevational range shifts. Due to the low overlap in species with the Kerner dataset of elevational range shifts and the thermal tolerance dataset (*n* = 8), knockdown temperature was not included. Again, model assumptions were checked prior to fitting and term significance was tested using Type II tests.

### Reporting summary

Further information on research design is available in the Nature Portfolio Reporting Summary linked to this article.

## Supplementary information

Transparent Peer Review file

Supplementary Materials

Reporting summary

## Data Availability

All data (including source data behind the figures in the paper) are available at 10.5281/zenodo.20492826^112^. Any remaining information can be obtained from the corresponding author upon reasonable request.

## Code availability

All analysis code can be found at 10.5281/zenodo.20492826^112^.
