# Supplementary material for: Butterflies with low thermoregulatory capacity show greatest upwards range shifts along an elevational gradient
Source: Commun Biol. 2026 Jul 1;9:1002. doi: 10.1038/s42003-026-10534-z (PMC13388664; doi:10.1038/s42003-026-10534-z)
Supplement: Supplementary file 2 — Supplementary Materials [file 42003_2026_10534_MOESM2_ESM.pdf]

Butterflies with low thermoregulatory capacity show greatest upwards range shifts along an elevational gradient

Supplementary Materials

Table S1: The survey sites ordered by elevation, with coordinates (latitude and longitude) given.

Site	Latitude	Longitude	Elevation (m)
1	47°35'24.94"N	12°50'7.33"E	820
2	47°34'39.57"N	12°48'34.07"E	940
3	47°35'22.95"N	12°53'34.87"E	1044
4	47°34'40.31"N	12°57'16.74"E	1105
5	47°34'33.98"N	13° 0'38.95"E	1306
6	47°33'13.16"N	13° 0'44.39"E	1461
7	47°31'32.60"N	13° 0'10.91"E	1553
8	47°32'13.72"N	12°59'42.88"E	1683
9	47°33'21.62"N	13° 1'52.47"E	1825

Table S2: The sites surveyed, ordered by date. Each visit was only conducted in good weather (not in rain, wind below 5 on the Beaufort scale). In total, each site was visited for six hours. If the weather changed and became unsuitable during a survey, the time was paused and the site was returned to on another day in suitable weather.

Site	Date surveyed
4	05/08/2025
1	06/08/2025
2	06/08/2025
3	07/08/2025
6	08/08/2025
9	08/08/2025
8	09/08/2025
7	09/08/2025
5	11/08/2025
4	12/08/2025
9	13/08/2025
1	14/08/2025

2	15/08/2025
3	18/08/2025
7	19/08/2025
5	20/08/2025
6	24/08/2025
8	25/08/2025
2	26/08/2025
1	26/08/2025
4	26/08/2025
9	27/08/2025

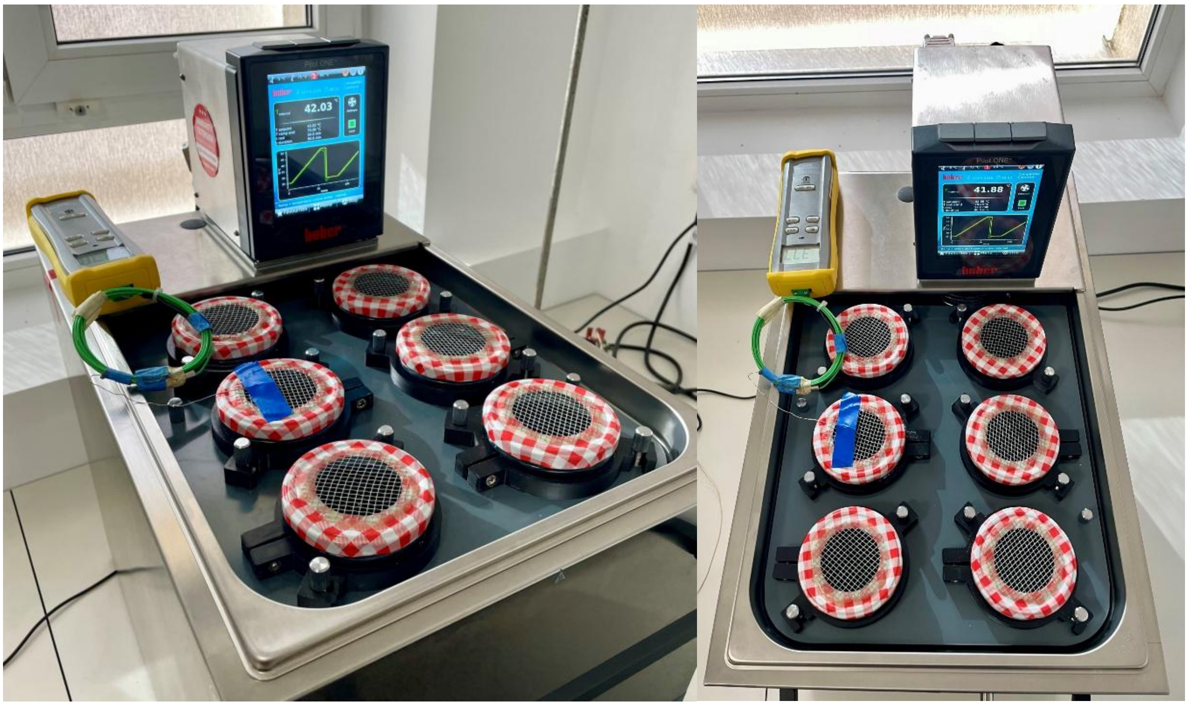

Figure S1: Photographs of the water bath set up. Six jars were suspended in the water, with one jar being allocated as the control jar which had a thermocouple placed 8 cm deep in to the jar to record internal air temperature (this was used for all measures of fall temperatures, not the temperatures from the water baths own thermometer, as this more accurately tracked the air temperatures that the insects were experiencing). Images taken by Esme Ashe-Jepson.

Table S3: The list of species (in alphabetical order), along with their sample size across all sites, the species-mean wing length, species-level colouration value (NIR reflectance in the basal portion of the wing, either dorsal for dorsal baskers and ventral for lateral baskers), the thermoregulation capacity (slope of body temperature and air temperature, inverted so that a high value indicates a better thermoregulation capacity (more stable slope)), and CTmax (LT50 (lethal temperature 50), the temperature at which 50% of individuals had fallen).

Species	Sample size	Mean wing length (cm)	Colouration (NIR reflectance)	Thermoregulation (inverted slope)	CTmax (LT50)
Aglais io	23	2.83	22.64	0.94	45.57
Araschnia levana	7	2.00	47.53	0.29	NA
Argynnis adippe	5	2.88	46.11	0.46	NA
Argynnis aglaja	8	2.96	40.77	-0.01	NA
Argynnis paphia	27	3.40	43.31	0.28	45.29
Aricia agestis	1	1.39	46.78	NA	NA
Boloria dia	1	2.32	48.65	NA	NA
Boloria titania	1	2.54	37.18	NA	NA
Colias alfacariensis	1	2.50	93.78	NA	NA
Colias crocea	4	2.52	89.20	0.27	NA
Colias hyale	2	2.48	88.42	NA	NA
Colias phicomone	2	2.71	76.61	NA	NA
Erebia aethiops	169	2.37	37.72	0.24	44.33
Erebia ligea	20	2.43	32.33	0.12	44.69
Erebia manto	26	2.10	18.13	0.07	45.83
Erebia melampus	11	1.96	20.13	0.16	44.60
Erebia meolans	1	2.46	20.09	NA	NA
Erebia pronoe	54	2.27	42.09	0.28	46.48
Gonepteryx rhamni	4	2.91	99.74	-0.62	NA
Hesperia comma	61	1.44	34.14	0.22	46.46
Leptidea sinapis	1	2.06	92.13	NA	NA
Lycaena virgaureae	7	1.67	34.15	0.53	46.78
Maniola jurtina	18	2.25	37.08	0.44	45.00
Melitaea athalia	1	2.01	30.50	NA	NA
Papilio machaon	1	3.74	20.62	NA	NA
Parnassius apollo	1	3.96	37.05	NA	NA
Pieris mannii	1	2.65	59.47	NA	NA
Pieris napi	30	2.48	39.35	0.24	43.54
Pieris rapae	14	2.40	64.23	0.49	NA
Polyommatus bellargus	2	1.60	19.69	NA	NA
Polyommatus coridon	36	1.71	16.84	0.22	46.62
Polyommatus icarus	11	1.50	24.58	0.25	44.07
Polyommatus thersites	2	1.33	25.07	0.29	NA
Pyrgus malvae	2	1.48	27.59	NA	NA

Thymelicus sylvestris	11	1.43	33.43	0.08	NA
Vanessa cardui	2	3.10	29.83	NA	NA

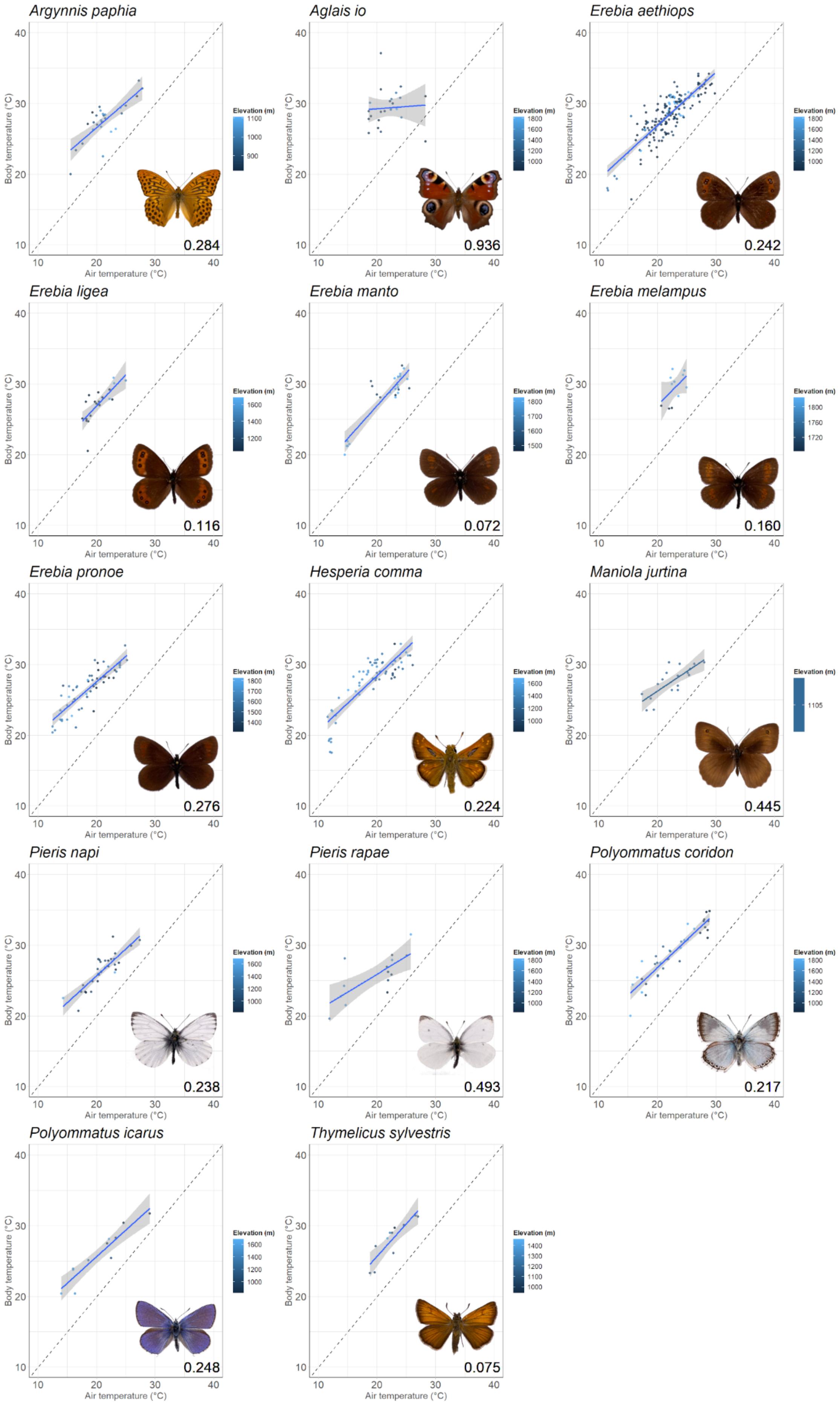

Argynnis paphia
0.284
Aglais io
0.936
Erebia aethiops
0.242
Erebia ligea
0.116
Erebia manto
0.072
Erebia melampus
0.160
Erebia pronoe
0.276
Hesperia comma
0.224
Maniola jurtina
0.445
Pieris napi
0.238
Pieris rapae
0.493
Polyommatus coridon
0.217
Polyommatus icarus
0.248
Thymelicus sylvestris
0.075
Body temperature (°C)
Air temperature (°C)
Elevation (m)

Figure S2: The relationship between body temperature and air temperature for all species with over 10 records, plotted separately (ordered alphabetically). Points represent individual butterflies, coloured by the elevation at which they were caught. Lines represent predicted responses, with shaded ribbons indicating 95% confidence intervals. Note that all axes have been standardised to ease comparisons. The slope values are given per plot to ease interpretation (inverted so that a high value indicates a strong thermoregulatory performance (shallow slope) and a low value indicates a poor thermoregulatory performance). Photographs are given of pinned butterflies to demonstrate the species, note that all are males for consistency.

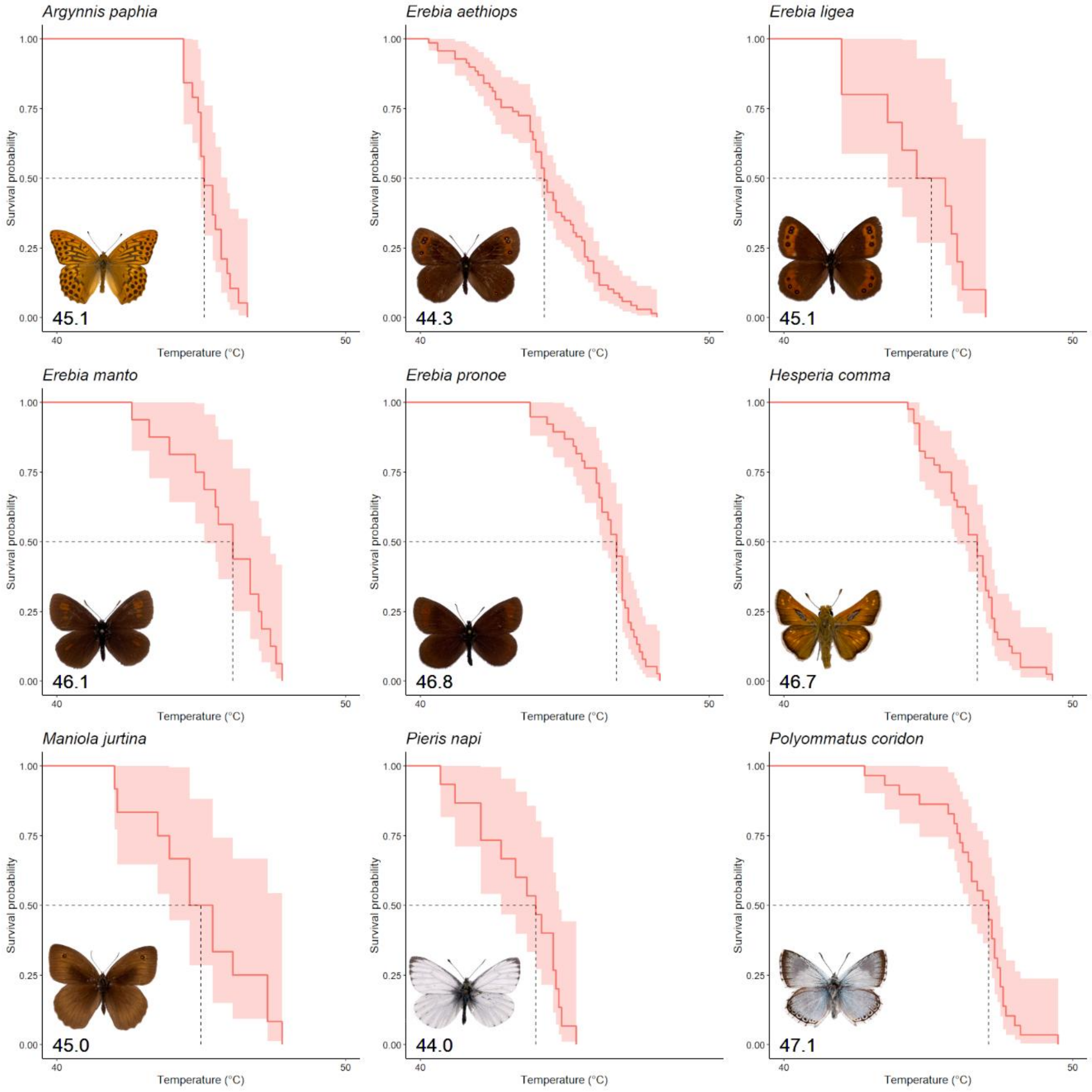

Figure S3: Survival curves for all species tested with at least 10 records, plotted separately by species (ordered alphabetically). The curves show the predicted survival probability, with coloured ribbons indicating 95% confidence intervals. Note that all axes have been standardised to ease comparisons. The dashed lines indicate the LT50 (lethal temperature 50) value for each species, the temperature at which 50% of individuals had fallen in the heat

knockdown assay. Values for the LT50 are also given per plot. Photographs are given of pinned butterflies to demonstrate the species, note that all are males for consistency.

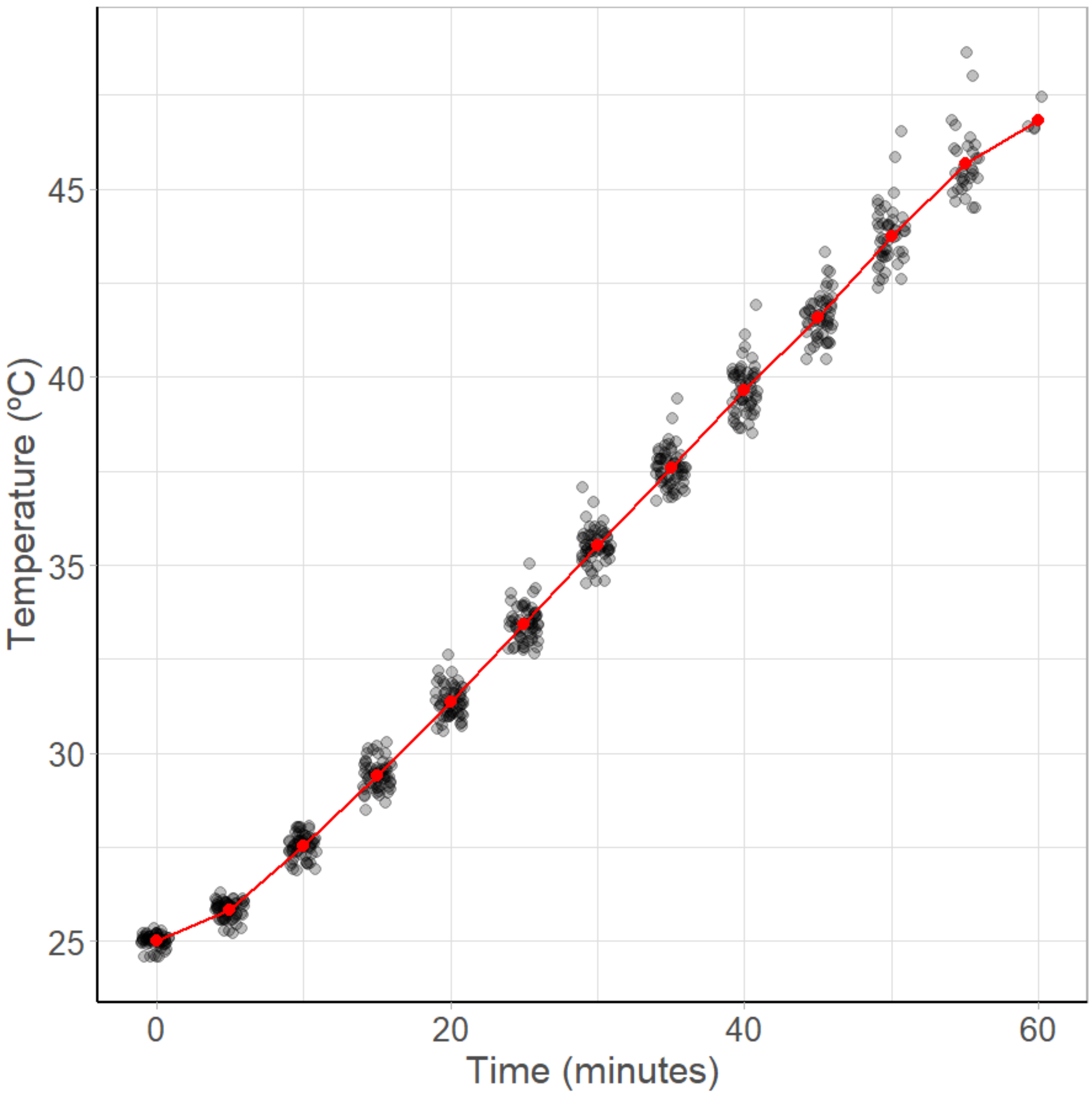

Figure S4: The achieved ramping rate of the water bath across all runs (n = 62). Black points represent individual run temperatures at 5 minute intervals (from 0 to 60 minutes),. Red points indicate mean temperatures at each five minute.

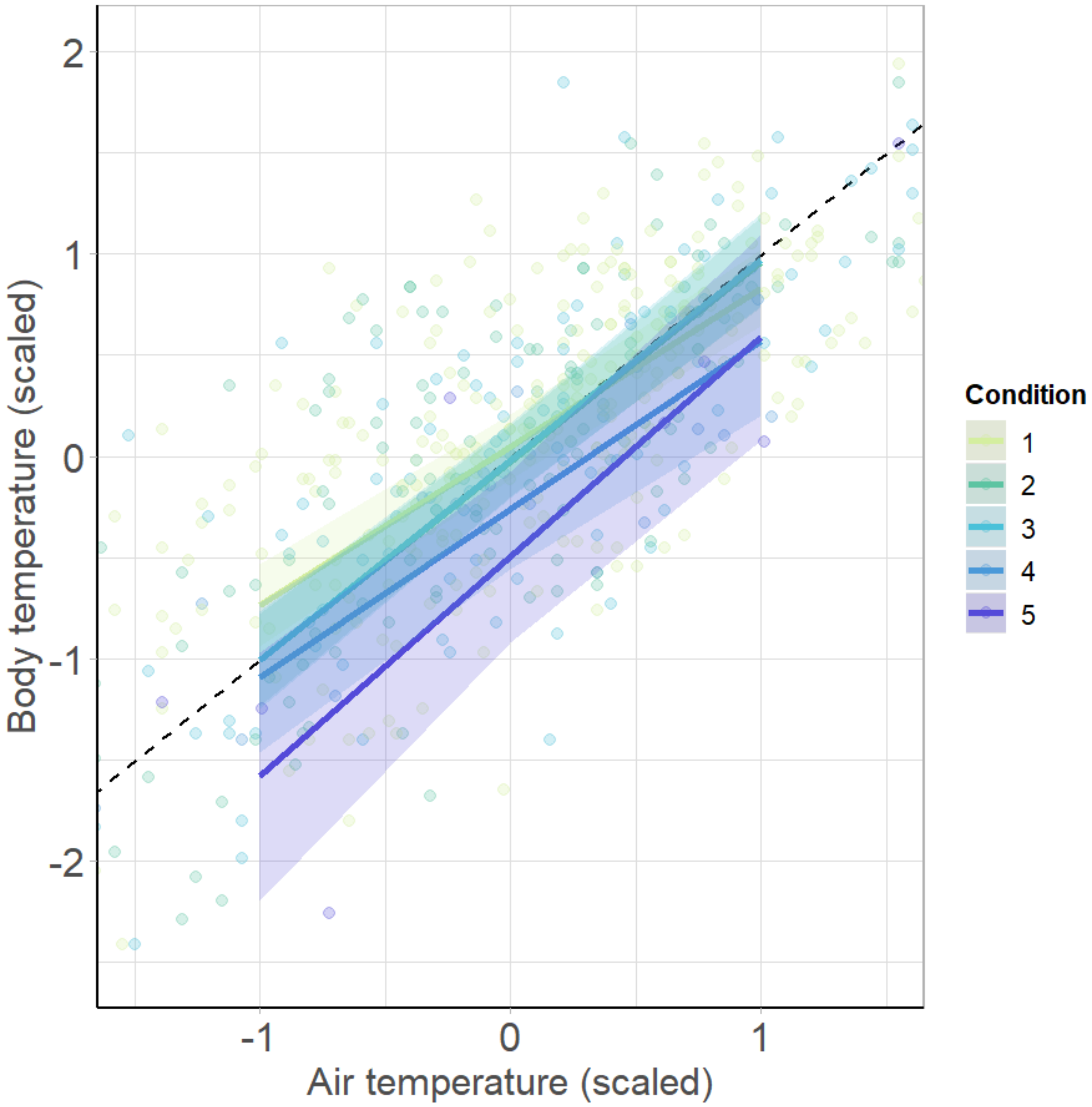

Figure S5: The relationship between body temperature and air temperature across the elevational gradient, split by wing condition (whereby 1 indicates perfect condition with no scale loss, and 5 indicates substantial damage to the wings). Note that both axes have been scaled to ease interpretation, whereby units represent standard deviations with zero being the mean. The lines indicate predicted responses, the coloured ribbons indicate 95% confidence intervals. Points represent individual observations. The dashed 1:1 line is to aid interpretation.

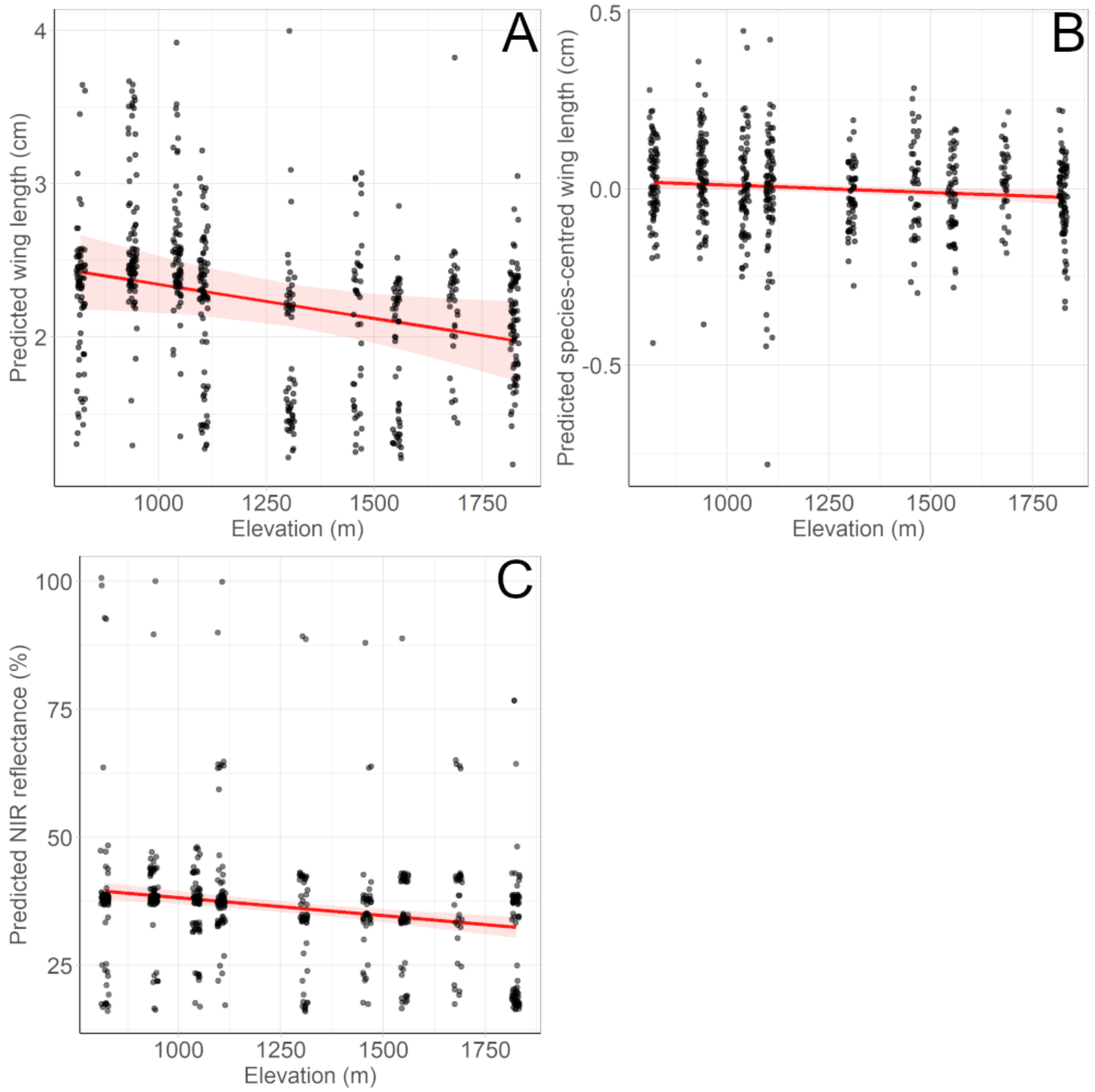

Figure S6: The change in (A) wing length, (B) species-centred wing length (where values above zero indicate an individual above average size for their species, and below zero indicates an individual below average size for their species), and (C) near-infrared (NIR) reflectance across the elevational gradient. The points represent individual butterflies and have been jittered to more clearly show overlapping points, the red line shows the predicted response. The coloured ribbons indicate 95% confidence intervals.
