# Supplementary material for: Butterflies with low thermoregulatory capacity show greatest upwards range shifts along an elevational gradient
Source: Commun Biol. 2026 Jul 1;9:1002. doi: 10.1038/s42003-026-10534-z (PMC13388664; doi:10.1038/s42003-026-10534-z)
Supplement: Supplementary file 1 — Transparent Peer Review file [file 42003_2026_10534_MOESM1_ESM.pdf]

nature portfolio

Peer Review File

Butterflies with low thermoregulatory capacity show greatest upwards range shifts along an elevational gradient

Corresponding Author: Dr Esme Ashe-Jepson

This file contains all reviewer reports in order by version, followed by all author rebuttals in order by version.

Version 0:

Reviewer comments:

Reviewer #1

(Remarks to the Author)
Summary

The manuscript “Species redistribution, not adaptation, drives butterfly responses to climate change” investigates whether butterfly thermal traits (thermoregulation and thermal tolerance) and functional traits (wing length and near-infrared reflectance) vary along an elevational gradient, and whether these traits predict recent elevational range shifts. The author report limited evidence for intraspecific adaptation in thermal traits, strong trait-dependent thermoregulation under warmer conditions, clear elevational shifts in functional composition (smaller, darker species at high elevation; larger, paler species at low elevation), and greater upward shifts in species with poorer thermoregulatory capacity.
Overall, this is a well-written manuscript that I much enjoyed reading. Experimentally the study is well-designed and very well-performed, while the analyses are relevant. The author is aware of up-to-date literature and have a deep ecological understanding and scientific knowledge with regards to the paper scope.
This study is an outstanding contribution to existing literature because it provides data on thermal traits of butterflies that are largely lacking but are key aspects of species resilience to climate change.

I provide below some general thoughts and minor suggestions, hoping to be of use for the improvement of the paper.

General comments
Discussion
The core findings are strong, but the Discussion is overly long and at times repetitive. Several sections restate results or explore multiple speculative mechanisms in detail. Reducing the Discussion by ~30% would improve clarity and impact.
Body size patterns and temperature–size expectations
The decrease in wing length with increasing elevation is interpreted in the manuscript as consistent with converse Bergmann’s rule (smaller body size in colder environments). While this interpretation is well explained, it would be helpful to more explicitly acknowledge that there is also substantial literature showing that increased temperatures can drive butterflies and other ectotherms toward smaller body sizes, consistent with the Temperature-Size Rule (TSR).
Repetition of research questions
What stands out is how the study questions get repeated across sections. Instead of showing up repeatedly, they could be trimmed down so each one only appears once.

Minor comments
-Where multiple papers by the same first author from the same year are cited (e.g., Ashe-Jepson et al., 2023), these should be distinguished consistently as 2023a and 2023b in both the in-text citations and the reference list.
-Please ensure consistency in notation (°C formatting; df vs d.f.).
-While I was reading the MS, I was thinking it would be great to have a visualization of the thermal tolerance experiment. Your description is very detailed but having, in addition to the text, that visual record, could make results feel more open and repeatable. Please consider including some photographs in the SI file.
- Given that this is single author work, the author may wish to consider using singular pronouns (“I”) instead of “we” for consistency.

Reviewer #2

(Remarks to the Author)
Ashe-Jepson presents a fascinating comparative study using butterflies to suggest that species-level traits rather than changes with species are determining how organisms respond to climate change. This work combines an impressive number of field measurements across an elevational gradient in dozens of species to show that species with less physiological tolerance to heat stress are shifting to higher elevations and species traits such as coloration are influencing who can survive at different elevations. This work will be of interest to a broad range of readers, including ecologists, evolutionary biologists and environmental scientists. However, in its current form, there are several limitations.

First, throughout the paper, the text uses the word “adaptation” in a somewhat confusing manner. Many readers (myself included) would assume “adaptation” to refer to an evolutionary process, and, indeed, several references throughout the text suggest this is how it is intended. However, the method of measuring adaptation (intraspecific variation matched to local conditions) can not rule out phenotypic plasticity. If anything, I think it’s more interesting that there is no evidence of variation within species along the elevational gradient due to plasticity (or adaptation) given how flexible some of these traits are developmentally. I think the phrasing needs to be adjusted throughout the manuscript to be more transparent about what was measured, for instance “intraspecific matching” which would encompass both evolutionary and developmental processes to explain the pattern of interest.

Second, given the environmental dependence of these measures, more information is needed on the sampling conditions. The methods state “Sites were sampled in August 2025 in three two-hour periods, so that each site was visited three times and sampled for a total of six hours, to ensure even sampling effort. Sites were only visited in suitable weather, that being not raining or high wind speed conditions (above five on the Beaufort scale).” It would be useful to know the order in which sites were sampled and how time of day and month varied with elevation since temperature likely varies with those sampling factors in consistent ways. In addition, the results lists an impressive number of individual butterflies that were sampled during 6 hours x each site (>500) – were multiple people running the captures and data collection, in which case was there any different between observers in some of the subjective measurements such as wing wear?

Third, several of the most interesting comparisons in the study rely on comparisons across the species. However, it does not appear that any attempt was made to correct for phylogenetic relationships across species. (or perhaps I missed that) This is concerning because phylogeny does seem to be closely tied to coloration for the subset of species sampled, for instance most of the dark butterflies are from two clades of grass-feeding butterflies (Satyrinae and Hesperiinae) and most of the light butterflies are from a different group (Pierinae and two Lycaenids). I wonder how much the statistical confidence of these comparative analyses would change if relatedness was accounted for.

Finally, several of the primary findings in the results are presented as plots that do not include any of the raw data (Figure 2 and 3, with 4 panels each). While I understand the figure looks much cleaner to just present the model output, it is much more useful (and transparent) to include data points that reflect the measurements that generated that model output. Such points are reflected in some of the supplemental figures, which are quite useful. I would suggest including them in the plots in the main figures as well so it is easier for the reader to understand patterns of underlying variation across species and sites.

A more minor note, there are a few wording things related to temperature changes and temperature knock-down that are confusing. The methods suggest that the temperature known-down experiment in the water bath had a rising temperature until the individual butterfly was unresponsive. But then the butterfly was released. Later, the analyses, and wording in the figure legends (“lethal temperature”) related to a survival analysis suggests the individuals died. Or perhaps this is a survival analysis applied to temporary knockdown? If the latter, clarification in the figure legend would be helpful. If the former, clarifying which butterflies were released in the methods would be helpful.

On a related note, several parts of the results discuss differences across butterflies in “heating rates” and “heating up.” This is confusing because as far as I can tell, these results apply to the differences across individuals in body temperature and “rate” applies to the differences across individuals captured in different temperatures, and the only part of the experiment that directly measured heating rates was the CTmax experiment where butterfly temperatures were not measured, instead their response to rising temperatures. I suggest choosing more clear and consistent language and/or adding a figure to clarify the two approaches to measuring responses to temperature.

Reviewer #3

(Remarks to the Author)
This manuscript reports a space-for-time-substitution study of butterfly thermal and other functional traits along an elevational gradient in the Austrian Alps to determine how butterflies are responding to climate change. The two general types of butterfly responses considered are trait adaptation and trait-dependent changes in geographic distribution. That intraspecific variation in thermal traits was not associated with the elevational gradient was interpreted as evidence against trait adaptation. By contrast, combinations of thermal and other functional traits showed complex patterns of variation over the elevational gradient. Most importantly, one thermal trait (thermoregulatory capacity) and one other functional trait (wing length) were associated with previously reported elevational changes in butterfly species from the same region. The authors conclude that such traits are driving geographic changes in individual species as the main responses to climate change.

This study complements other literature, especially Bladon et al. (2020), which shows that in UK butterflies "the effectiveness of behavioural thermoregulation compared to microclimate selection predicted species' long-term population trends, with those relying more on microclimate selection experiencing more negative population trends over the last 40 years." In the present manuscript, the authors show that the same thermoregulation trait predicts "elevational range shift, whereby species with poorer ability to thermoregulate (low thermoregulatory capacity) showed larger upwards elevational range shifts." Taken together, the evidence suggests that European butterfly species possessing high thermoregulatory capacity are more resistant to climate warming than species with low thermoregulatory capacity. This and other recent studies are thus identifying specific traits that determine species responses to climate change. To me, this is the strongest takeaway from this study. I see this as an important contribution to our understanding of how species in natural ecological communities are responding to climate change.

However, I also have critiques about the methodology and interpretation of the results, leaving me unconvinced by some of the other conclusions drawn. I think the authors should address the following.

1. The space-for-time-substitution (SFTS) approach is questionable for the forecasting interpretations. As the authors point out in the Discussion section, a recent commentary piece (Evans et al. 2025, Nature Climate Change) critiques the SFTS approach in research that forecasts ecological consequences of climate change and suggests alternative approaches. The two main problems with SFTS are: 1) misattributed causality of climatic variables that are spatially correlated with ecological response variables, and 2) failure to distinguish transient from equilibrial ecological responses to climate change. The trait of wing length is questionable because there is no strong evidence for a causal link between climate and the trait. Perhaps there is such a link, but it also might be spatially correlated with ecological response variables to climate change. If I am perhaps overlooking the causal evidence, then I suggest the authors put greater emphasis on this point. Other traits (thermoregulation, thermal tolerance, wing coloration) that are more clearly relevant may also be correlated with other traits (e.g., behavior, temporal/spatial activity) that causally respond to climate change. A more nuanced issue with these traits is how they are causally related to weather versus climate. This distinction is not explicitly considered in this manuscript. For example, what is meant exactly by "extreme heat" in the conclusion: "This implies that small and dark species will be particularly vulnerable to extreme heat" (Abstract). If the thermal traits are most relevant to weather, they would function to help individual butterflies deal with the thermal variation they experience (i.e. acclimation). A critical question is how phenotypically plastic these traits are. If the traits are relevant to climate, variation in such traits would be associated with variation in climate over decades. The analysis addressing question 3 in the manuscript is the most convincing one because it shows such an association between thermoregulatory capacity and decadal elevation shifts.

2. Thermoregulatory capacity is misleadingly called "heating rate" in some places (Abstract, Data analysis part of Methods, Results section). This trait is quantified in linear mixed effects models as the intraspecific variation in butterfly body temperature as a function of air temperature where each butterfly was captured. It is not a rate. The linear slope of this regression has previously been called "temperature buffering" (e.g., Bladon et al. 2020), with shallow slopes indicating relatively high temperature buffering capacity and steeper slopes indicating the opposite. I suggest the authors go back to "temperature buffering" or consistently use "thermoregulatory capacity," which is also sometimes used in the present manuscript. But many statements are misleading, such as "Dark species had slower heating rates" (Abstract), and these should be removed.

3. The thermal tolerance trait is of questionable ecological relevance without further validation. If a butterfly approaches its thermal tolerance in the wild, perhaps it will probably find a shady place to rest and cool down. Many desert butterflies, for example, restrict their flight activity to early morning hours during the summer and spend the heat of the day resting in shady places. The desert butterflies are already adapted to extreme climate and extreme weather. Without further study of trait plasticity and how it relates to other functional traits (e.g., behavior), one cannot know how the thermal tolerance measures of butterfly species in the present study would predict their responses to a heat wave (weather) or climate change. It is reasonable to hypothesize that higher thermal tolerance by this measure would benefit butterflies subjected to either hot weather or a warming climate. But heat avoidance behaviors of the butterflies might predominate, rendering thermal tolerance less relevant. In sum, I don't think there is a strong enough basis for the prediction "that butterflies will not be able to compensate for increased body temperature by raising their thermal tolerance."

4. The limited sampling of many butterfly species along the elevational gradient (1000 meters) offers only a weak test of the possibility that the butterflies are responding to climate change via trait adaptation. As such, lack of evidence does not equate to evidence of absence. In Table S2, the sample size of each species per site is not shown. However, with one exception (Erebia aethiops, N = 169 total), the sample size per species per site must be quite small. Aside from this outlier, the larger sample sizes range from 30-61 across 9 sites. It is not reasonable to expect to detect a signature of local adaptation based on 3-7 individuals per species per site. While the analyses are based on traits, which are shared among multiple species and thus may allow the authors to circumvent the sample size issue to some degree, there was no attempt to account for non-independence of traits shared by closely related species (i.e. no phylogenetic comparative approach). Again, it would be valuable to know how phenotypically plastic these thermal traits are. Otherwise, it is difficult to understand their function in relation to environmental variation as well as in relation to other co-functional traits. I see no strong basis for the conclusion "some sort of constraint or limit has been reached" in the evolution of thermoregulatory capacity. Likewise, I disagree with the assertion that this study "demonstrates that butterflies are responding to climate change through redistribution of species rather than adaptation in place." While this study and many before it show evidence for redistribution of species, the evidence here on adaptation in place is inconclusive.

Version 1:

Reviewer comments:

Reviewer #1

(Remarks to the Author)
I appreciate the extent of revisions made by the author. I am content with the revisions made and am happy to recommend the manuscript be published.

Reviewer #2

(Remarks to the Author)
The author has done an incredibly job addressing the initial round of reviews. Not only have extensive changes been made to the introduction to clarify terms and phrasing, but there are also new analyses to control for phylogeny and updated figures to show individual data points. New data on weather differences across sites reassures the reader that variation in weather on sampling days is not confounding the findings. I am very impressed by the breadth and depth of the revisions. This addresses all of my concerns in terms of alternate explanations and interpretations. This research will make a big impact on the field.

Reviewer #3

(Remarks to the Author)
To further the conversation, I have pasted my previous comments along with the author's rebuttal comments before my new round of responses (see each "New reviewer response"). I also added line-specific comments to make my critical comments more detailed and constructive.

This manuscript reports a space-for-time-substitution study of butterfly thermal and other functional traits along an elevational gradient in the Austrian Alps to determine how butterflies are responding to climate change. The two general types of butterfly responses considered are trait adaptation and trait-dependent changes in geographic distribution. That intraspecific variation in thermal traits was not associated with the elevational gradient was interpreted as evidence against trait adaptation. By contrast, combinations of thermal and other functional traits showed complex patterns of variation over the elevational gradient. Most importantly, one thermal trait (thermoregulatory capacity) and one other functional trait (wing length) were associated with previously reported elevational changes in butterfly species from the same region. The authors conclude that such traits are driving geographic changes in individual species as the main responses to climate change. This study complements other literature, especially Bladon et al. (2020), which shows that in UK butterflies "the effectiveness of behavioural thermoregulation compared to microclimate selection predicted species' long-term population trends, with those relying more on microclimate selection experiencing more negative population trends over the last 40 years." In the present manuscript, the authors show that the same thermoregulation trait predicts "elevational range shift, whereby species with poorer ability to thermoregulate (low thermoregulatory capacity) showed larger upwards elevational range shifts." Taken together, the evidence suggests that European butterfly species possessing high thermoregulatory capacity are more resistant to climate warming than species with low thermoregulatory capacity. This and other recent studies are thus identifying specific traits that determine species responses to climate change. To me, this is the strongest takeaway from this study. I see this as an important contribution to our understanding of how species in natural ecological communities are responding to climate change.
• Thank you for your kind comments on the manuscript. I hope I have addressed your concerns below.

However, I also have critiques about the methodology and interpretation of the results, leaving me unconvinced by some of the other conclusions drawn. I think the authors should address the following. 1. The space-for-time-substitution (SFTS) approach is questionable for the forecasting interpretations. As the authors point out in the Discussion section, a recent commentary piece (Evans et al. 2025, Nature Climate Change) critiques the SFTS approach in research that forecasts ecological consequences of climate change and suggests alternative approaches. The two main problems with SFTS are: 1) misattributed causality of climatic variables that are spatially correlated with ecological response variables, and 2) failure to distinguish transient from equilibrial ecological responses to climate change. The trait of wing length is questionable because there is no strong evidence for a causal link between climate and the trait. Perhaps there is such a link, but it also might be spatially correlated with ecological response variables to climate change. If I am perhaps overlooking the causal evidence, then I suggest the authors put greater emphasis on this point. Other traits (thermoregulation, thermal tolerance, wing coloration) that are more clearly relevant may also be correlated with other traits (e.g., behavior, temporal/spatial activity) that causally respond to climate change. A more nuanced issue with these traits is how they are causally related to weather versus climate. This distinction is not explicitly considered in this manuscript. For example, what is meant exactly by "extreme heat" in the conclusion: "This implies that small and dark species will be particularly vulnerable to extreme heat" (Abstract). If the thermal traits are most relevant to weather, they would function to help individual butterflies deal with the thermal variation they experience (i.e. acclimation). A critical question is how phenotypically plastic these traits are. If the traits are relevant to climate, variation in such traits would be associated with variation in climate over decades. The analysis addressing question 3 in the manuscript is the most convincing one because it shows such an association between thermoregulatory capacity and decadal elevation shifts.

• Thank you for raising this important point. Firstly, in butterflies there are several papers that look at wing length and thermoregulatory capacity and thermal tolerance, see: https://doi.org/10.1111/1365-2656.13319, https://doi.org/10.1111/1365-2656.13970, https://doi.org/10.1111/1365-2656.14039), showing that this trait is influential with

the ways that butterflies interact with environmental temperature. The expected causal impact of wing length is how it correlates strongly with body size in butterflies, and therefore will impact the thermodynamics of the organism. In butterflies, wing length also acts as a determinant of flight capacity/dispersal ability. In that case, it will impact how fast and efficiently a butterfly can search an area for a suitable microclimate. It may also impact which sorts of microclimates a butterfly is inclined to use, though that so far as never been tested. Both size and colouration were chosen because of their expected impacts on the thermodynamics of the insects, and the previous evidence collected in butterflies that demonstrates that these particular traits are important.

New reviewer comment:
I am satisfied with the author’s response on this point.

• To address your comment of what is meant by ‘extreme heat’, in this case ‘extreme heat’ is extreme weather events, such as heatwaves which are increasingly common in the region (in fact, there was a heatwave in the alpine study region sadly before I started sampling (but was setting up my study sites, this was in June-July), where I recorded air temperatures up to 30C in my highest site (approx. 1800 m). Minor changes in mean air temperature are unlikely to elicit strong responses from my study species, particularly as they are likely to benefit butterfly activity rather than hinder it (considering their relatively high thermal requirement in their flight muscles for active flight). Instead, there is growing evidence that temperature extremes (such as the horrible heatwave that hit Europe that summer, where some parts of Europe nearly reached 50C) will be bigger drivers of responses in butterflies than changes in mean temperatures (e.g., https://doi.org/10.1007/s10144-013-0409-y). Therefore, when discussing butterfly vulnerability to climate change, the importance of thermoregulation or thermal tolerance, I tend to focus more on extreme conditions than gradual changes to means, particularly in these relatively cool regions.

New reviewer comment:
The author’s response makes sense to me. As long as this is clear in the revised manuscript (and I believe it is clear now), I am satisfied.

• In relation to your comment about weather, firstly it is of note that my interest in this manuscript is understanding whether species essentially customise their thermal traits to suit local conditions (air temperature) across climates (elevation) (e.g., it would be expected that in a hot climate, having better heat avoidance strategies should be selected for, or higher upper thermal limits, or functional traits that correspond to those thermal traits). Something of note, throughout the surveying period I continuously recorded percentage cloud cover and wind speed, and I did a robustness check to compare my thermoregulatory model with and without weather variables (e.g. does local weather impact these results, does controlling for the effect of weather impact the results) and found that they did not improve the model nor did they impact thermoregulatory capacity. I personally feel that this is probably the result of air temperature already accounting for all the variation in weather, as this was recorded for every individual in the location they were found, rather than taking daily means for example. If you would like, I have left this robustness check in my analysis code which I will make available with the manuscript should it be accepted, but I could include this in the Methods if you feel this would be valuable. I am conscious of the length of the Methods now that I have added in all the other robustness checks. Perhaps I could move all of these to the supplementary where I could go into more detail there without adding excessive length to the manuscript. I am open to your suggestions.

New reviewer comment:
Thanks for the clarification. I’m less concerned about the effects of weather conditions during the study than I am about interpretation of the thermal traits and their relationship to air temperature and elevation. In general, I find the thermoregulation trait most convincing because the relationship between body temperature and air temperature is clearly meaningful. However, without knowledge of the plasticity of this relationship (i.e. plasticity of thermoregulation), it is difficult to interpret the functional significance of this thermoregulation trait. For example, how much can this relationship vary within a species? Would temperatures or other cues earlier in development change it? I don’t think this is known for any butterfly species, but if it is known, this information would be important to discuss. Another challenge is the causal relationship between the observed elevational range shift and this thermoregulation trait across butterfly species. The author hypothesizes “that butterflies with a poorer ability to thermoregulate and with low flexibility in this trait will have shown stronger responses to recent climate change, so greater upwards elevational range shifts, as they should be less able to cope with changes to their local climatic conditions.” While I agree that this is a good hypothesis, it is also plausible that butterfly species with greater upwards elevational range shifts have been able to maintain their climate envelopes, thus shielding them from recent selection on thermoregulation traits. This alternative hypothesis proposes that the observed variation in thermoregulation capacity is a consequence of the strength of selection on this trait, which could be genetically fixed or close to fixed within each species. If this is true, it would result in the patterns observed in this study.

2. Thermoregulatory capacity is misleadingly called “heating rate” in some places (Abstract, Data analysis part of Methods, Results section). This trait is quantified in linear mixed effects models as the intraspecific variation in butterfly body temperature as a function of air temperature where each butterfly was captured. It is not a rate. The linear slope of this regression has previously been called “temperature buffering” (e.g., Bladon et al. 2020), with shallow slopes indicating relatively high temperature buffering capacity and steeper slopes indicating the opposite. I suggest the authors go back to “temperature buffering” or consistently use “thermoregulatory capacity,” which is also sometimes used in the present manuscript. But many statements are misleading, such as “Dark species had slower heating rates” (Abstract), and these should be removed.

• Thermoregulatory capacity in this sense is the slope between body temperature and air temperature, so this is a numerical

value that corresponds to how much one unit of body temperature changes with a unit increase in air temperature, which I would define as a rate. However, I agree that terminology in this case can be very confusing! I have personally published work on this metric using terms such as ‘buffering ability’ and the like, and find that it is not intuitive and requires quite a lot of explanation, particularly now that I work in a country where English is not the native language. Therefore I do not agree with the use of ‘buffering ability’ anymore and have been moving towards a more intuitive term and have more commonly been using ‘thermoregulatory capacity’ (for example see here where I use both: https://doi.org/10.1002/ece3.10623), as though this is also not perfectly intuitive, it is at least clear that this is related to how good something is at thermoregulating. In my experience of sharing my research with other people both inside and out of my field, this is easier to explain. However, in this case I was curious whether it was clearer to use heating rate rather than thermoregulatory capacity when referring only to the slope of the relationship (as previously it is unclear whether this also includes baseline body temperature). As this is obviously not clear, I have changed the text to ‘thermoregulatory capacity’ throughout when discussing the slope and removed mention of heating rates in all cases except where I am explicitly talking about rates of heat gain and thermodynamics.

New reviewer comment:
This metric of thermoregulatory capacity cannot tell you the rate of heating up in individual butterflies. It is a species-level trait that might indicate that individual butterflies heat up more or less rapidly as the environmental temperature increases. But it might alternatively reflect behavioral thermoregulation traits that enable individual butterflies to maintain a particular body temperature by heating or cooling despite cooler or warmer environmental temperature. Such behavioral traits include basking in sun, resting in shade, spending time in specific microclimates (vegetation, on the ground, etc.). Therefore, I continue to insist that “heating up rate” is not justified as a shorthand description of this metric, and I recommend removing this language throughout the manuscript.

3. The thermal tolerance trait is of questionable ecological relevance without further validation. If a butterfly approaches its thermal tolerance in the wild, perhaps it will probably find a shady place to rest and cool down. Many desert butterflies, for example, restrict their flight activity to early morning hours during the summer and spend the heat of the day resting in shady places. The desert butterflies are already adapted to extreme climate and extreme weather. Without further study of trait plasticity and how it relates to other functional traits (e.g., behavior), one cannot know how the thermal tolerance measures of butterfly species in the present study would predict their responses to a heat wave (weather) or climate change. It is reasonable to hypothesize that higher thermal tolerance by this measure would benefit butterflies subjected to either hot weather or a warming climate. But heat avoidance behaviors of the butterflies might predominate, rendering thermal tolerance less relevant. In sum, I don’t think there is a strong enough basis for the prediction “that butterflies will not be able to compensate for increased body temperature by raising their thermal tolerance.”

• Thank you for raising this. I acknowledge that of course behavioural thermoregulation alters the experienced body temperatures of butterflies (I include thermoregulation capacity as a focus point of the paper) and therefore alters the ecological relevance of upper thermal limits. Similarly, thermoregulatory capacity lacks context without upper thermal limits, which is why I felt both metrics interesting and complementary to include. However, physiological thermal limits are still widely considered an important component of species responses to warming (particularly to extreme heat rather than to gradual warming) as they define the upper boundary condition beyond which behavioural thermoregulation is no longer sufficient (for a recent Nature publication that used upper thermal limits to investigate insect vulnerability to climate change see https://doi.org/10.1038/s41586-026-10155-w). During extreme heat events, behavioural thermoregulation in small insects may not be able to buffer individuals from experiencing extreme body temperatures that approach their physiological limits (e.g., https://doi.org/10.1073/pnas.1316145111, also note this study that recorded butterfly body temperatures during an extreme heatwave in the UK and note that very few datapoints are lower than ambient, and those that are are very close to 1:1 showing thermoconformance rather than an ability to lower body temperature below ambient: https://doi.org/10.1007/s10841-024-00556-5). Previous work of mine has demonstrated a trade-off between thermoregulatory capacity and upper thermal limits in butterflies (see https://doi.org/10.1111/1365-2656.13970), highlighting that in some cases these traits can compensate for each other, which is where the choice of language comes from. I have altered the text however to remove this statement, as it feels too strong given the evidence.

New reviewer comment:
I agree that thermal tolerance is relevant during heatwaves, and the rebuttal comments are mostly reasonable. However, I still wonder about the way in which thermal tolerance was measured. As with the thermoregulation trait, the plasticity of thermal tolerance is not addressed and thus remains an open question. Does individual experience change thermal tolerance? Perhaps more pointedly, does the experience of being trapped and warmed at the rate used in this study reflect the thermal tolerance of each species? Perhaps so, but perhaps not. Species with high thermoregulatory capacity might be able to limit the rate at which a butterfly actually warms up during a real heat wave, perhaps giving the insect enough time to acclimate (increase its physiological tolerance to extreme high temperature). Such a scenario is consistent with “a trade-off between thermoregulatory capacity and upper thermal limits.” With respect to evolutionary adaptation, can thermal tolerance change under selection? And more pointedly, does the method used to measure it here reveal any heritable phenotypic variation on which selection can act within each species? Lastly, I appreciate that this thermal tolerance metric has been used previously in other published work, and I don’t doubt that it might truly reflect the thermal tolerance of each butterfly species. Even so, I don’t see a strong validation of the method, which in turn warrants a more cautious conclusion.

• To be more specific on this given system, this study took place in open grasslands with little shade or shelter, therefore limiting heat avoidance. Activity would innately expose these insects to heat, so in this case, microhabitats may not be able to buffer species from extreme heat during heatwaves. In addition, landscapes around the world are being homogenised, and therefore also thermally homogenised, risking disruption to processes such as behavioural thermoregulation in small

insects, therefore this lack of response in upper thermal limits to elevation may be of wider interest. Though behavioural thermoregulation can buffer heat exposure, physiological tolerance remains a relevant trait to investigate and compare small ectotherm vulnerability under extreme conditions. Again, to date there is no evidence that butterflies can lower their body temperature below ambient conditions, so should ambient conditions reach physiological limits, behavioural thermoregulation may not be able to buffer this (for example the heatwave that hit Europe in 2025 had air temperatures nearing 50C, above the upper thermal limits of every species tested, unfortunately these heatwave temperatures are increasingly common and extreme). I have added text and references to the Discussion to better highlight this and highlight the role and limitations of upper thermal limits when considering species vulnerability to climate change.

New reviewer comment:
Substrate surface temperatures can differ dramatically even with full sun exposure. Consider the design of green roofs in built environments. Some studies show that a green roof (surface area mostly covered with plants) can have a surface temperature as much as 10 degrees C below that of a non-green roof. The same principle applies to a grassland. A butterfly can position itself in a much cooler microenvironment simply by landing on vegetation, especially if resting on the underside of a leaf or another part of vegetation shaded by leaves. So, while I agree that thermal limits might still be reached during a heatwave, butterflies and other animals have multiple behavioral thermoregulatory options to reduce heat exposure even in non-forested environments. I also grant that it is possible that the method used here to assess thermal limits might reflect such thermal limits of free-living butterflies. However, without further validation of the method, I am not convinced that it does. This is why I don't agree that this method can currently give highly conclusive results with respect to butterfly responses to temperature.

4. The limited sampling of many butterfly species along the elevational gradient (1000 meters) offers only a weak test of the possibility that the butterflies are responding to climate change via trait adaptation. As such, lack of evidence does not equate to evidence of absence. In Table S2, the sample size of each species per site is not shown. However, with one exception (Erebia aethiops, N = 169 total), the sample size per species per site must be quite small. Aside from this outlier, the larger sample sizes range from 30-61 across 9 sites. It is not reasonable to expect to detect a signature of local adaptation based on 3-7 individuals per species per site. While the analyses are based on traits, which are shared among multiple species and thus may allow the authors to circumvent the sample size issue to some degree, there was no attempt to account for non-independence of traits shared by closely related species (i.e. no phylogenetic comparative approach). Again, it would be valuable to know how phenotypically plastic these thermal traits are. Otherwise, it is difficult to understand their function in relation to environmental variation as well as in relation to other co-functional traits. I see no strong basis for the conclusion "some sort of constraint or limit has been reached" in the evolution of thermoregulatory capacity. Likewise, I disagree with the assertion that this study "demonstrates that butterflies are responding to climate change through redistribution of species rather than adaptation in place." While this study and many before it show evidence for redistribution of species, the evidence here on adaptation in place is inconclusive.

• Thank you for raising this valid concern. Firstly, I want to address your point about the sample size. This study took place within a national park in Germany, where all butterflies are protected by law (and especially in such areas). This national park is also the only part of the Alps in Germany, so other research sites were not accessible for this project. To conduct this research project, permits had to be acquired that imposed limits on numbers of butterflies that could be caught within a strict time limit that was set by the local governing authority. As such, unfortunately it is not possible to increase the sample size further, though I acknowledge that smaller sample sizes reduce statistical power to detect responses. Unfortunately, sample size limitations are very common in field studies of wild populations, particularly on protected species in protected areas.

New reviewer comment:
I appreciate that it was not possible to obtain large sample sizes of each species in this study. I want to be clear that I am not asking the author to increase the sample sizes. My point is that most of the sample sizes per species per site are small, thus there is limited power to detect a signature of trait adaptation. I am asking the author to interpret the results accordingly.

• I want to explain how the analysis should have helped to control for this. Species was always included as a random effect, rare species should therefore contribute to the overall relationships, but not get much weight in estimating their species-specific intercept. However, I feel this is important to empirically test. I have added a series of checks in the analysis to test for the impact of species-specific sample size on the results (i.e. were rare species obscuring patterns visible in the more abundant species). First, I remove all rare species (sample size < 10) and refit the models. This showed no difference in coefficient estimates strength or direction. For a stronger check, I then did a k-fold cross-validation, comparing how the model predictions were impacted by sample size (again comparing the full dataset to only species with >10 observations), here again coefficients were consistent in strength and direction, and the full model predicted slightly better. I checked whether the differences between the models were because of just sample size, or whether the rare species themselves were contributing something meaningful, so I ran another k-fold CV comparing a model trained on only the most abundant species against a random subsample of the abundant species but at the same sample size as the full dataset. These two performed very similarly, and both outperformed the subset model, indicating that sample size marginally improved the model predictive power, and the inclusion of rare species did not alter the performance. I did something similar for the Cox models, where I used the concordance index instead, and again found very little difference between the models (difference in C-statistic of 0.002). I have added this to the Methods section for both analyses as a robustness check, and it can be found in the associated code. These tests can only tell me whether or not the sample size per species is impacting the models, I cannot explicitly test for whether more data (that I don't have) might change the results. However, one thing I can do is as you mention, E. aethiops has a high sample size across the gradient. I have tested this species in isolation for both analyses, and the results are consistent with the full models (no change in thermoregulatory capacity across the gradient; no change in upper thermal limits across the gradient). If it would be valuable, I could include this in the manuscript, at present it

is only present in the code (which will be published alongside the manuscript). I am open to your suggestion on this.

New reviewer comment:
Thank you for adding the new analyses. They convinced me that phylogenetic non-independence is not influencing the results or conclusions here. Adding the analysis of E. aethiops would be the most powerful way to demonstrate no evidence of local adaptation in thermoregulatory capacity or thermal tolerance across the elevational gradient. However, I still believe additional methodological (experimental) approaches would be needed to provide a strong test of the local adaptation hypothesis.

• _I have also included phylogenetic sensitivity checks for each analysis (comparing non-phylogenetic models with phylogenetic models, and testing for phylogenetic signals in the response variables), which have been included in the Methods. Something of note is that the results found in this project align with the existing literature, where no difference in thermoregulatory capacity has been found across much wider geographic comparisons (e.g. comparing same species in UK and Spain: https://doi.org/10.1111/1365-2656.14039 and UK and Panama (https://doi.org/10.1111/gcb.16797). However I feel it is important to be upfront with the reader about the sample sizes, so I have added a caveat to the Discussion to highlight that larger sample sizes may allow for greater possibility of detection. Please note that the vast majority of species were not found across the entire gradient (with a few notable exceptions such as E. aethiops and interestingly L. coridon, and a few others).

New reviewer comment:
The phylogenetic sensitivity checks are helpful. Perhaps it would be worth arguing that the similarity in thermoregulatory capacity found across much wider geographic comparisons suggests that this trait is not likely to show local adaptation at a smaller geographic scale. Even so, this doesn't mean that other relevant traits are not adapting to climate change. Furthermore, if there is adaptive plasticity of thermoregulatory capacity, the observed comparisons might be obscuring it.

• _In this case I found no evidence for consistent shifts in thermal traits across the gradient within this study system, which goes against my hypothesis of species optimising these traits in suit their local climate. Instead it appears that these are intrinsic properties of species with limited capacity to change, at least within the given system. However, we did detect strong interspecific differences according to size and colour, which aligns with the later results of species redistributions (tracking the climate rather than adapting in place), as well as historic trends from paleoecology of insects generally redistributing rather than adapting in place. However, I want to make it clear to the reader that in this case, we cannot exclude the possibility that there is something occurring here, but that we just have insufficient evidence to suggest that species do not adjust their thermoregulatory capacity (similar to all other studies on butterflies), but that a more detailed analysis with a higher sampling intensity may detect different patterns. I have added text to the Discussion to highlight this important caveat, and thank you for raising this concern.

New reviewer comment:
I am convinced by the evidence in this study and many other studies that a major butterfly response to climate change is species redistribution. Indeed, it is this pattern and the high dispersal ability of most butterfly species that leads me to wonder to what extent these species are able to evade selection from climate change on other phenotypic traits (such as thermoregulatory capacity and thermal tolerance).

Line-specific comments:
Line 23. Add "of butterflies" before "across an elevational gradient…"
Line 25. Change "are fixed" to "might be fixed."
Lines 25-26. Better wording would be: "At low elevations, large species were better than small species at avoiding high body temperatures."
Lines 28-29. The meaning of this sentence is not clear.
Line 32. Remove "rather than adaptation in place" because there is not enough evidence to draw this conclusion.
Line 33. Add "ecological" before "responses to climate change."
Lines 48-49. Because local extinction is not a mechanism species rely on, the juxtaposition of these two sentences is problematic. Perhaps, "Crucially, whether species rely on dispersal or adaptation in place has direct conservation…"
Line 59. Change "upscale" to "scale up."
Line 62. The comma after "equally" should be a semicolon.
Line 66. Consider the following for the citation here: Warren et al. 2018, Science 360: 791-795.
Lines 93-95. I agree with this final sentence of the paragraph, but I didn't see much follow-up to this expectation in the Discussion.
Lines 137-138. The constraint interpretation would be true if the thermal traits don't change and the organisms suffer for it. There's no evidence here that the butterflies are constrained in adaptation because there is no measure of phenotypic selection.
Line 194. Flexibility in thermoregulatory capacity was not measured or analyzed, as far as I can see.
Lines 196-197. This is where it would be worth mentioning the alternative hypothesis that species with greater upslope shifts may have avoided selection on thermal traits. This hypothesis can also explain the negative correlation between thermoregulatory capacity and observed elevational range shift.
Line 197. I believe "rapid heating" refers to thermoregulatory capacity. Please correct this language.
Lines 214-215. Describe the systematic process used to sample butterflies.
Lines 261-263. If there is justification, provide it to validate the claim that a heating rate of 0.5 degrees C per minute over 90 minutes simulates a heat wave.
Lines 348, 395, 485. Replace "fall" with "knockdown" for clarity.

Line 356. This AIC difference looks quite large. Is the meaningful difference here not 2? Why not?
Line 414. Remove “heated up faster” and replace with accurate language.
Lines 422, 426, 431. Remove “heated up more slowly” and replace with accurate language.
Line 425. Remove “heated up more quickly” and replace with accurate language.
Line 539. Leading the Discussion section with this statement is giving it more credence than is warranted. I strongly suggest restructuring the Discussion section so it 1) leads with the strongest evidence (associations between elevational range shifts and traits measured in this study), and 2) explicitly addresses the questions and hypotheses given in the Introduction section. There is a lot of speculative content in the Discussion that goes too far beyond the evidence.
Line 542. This is the first of several cases where the author is equating elevation and temperature without acknowledging the many other factors that are probably (or known to be) associated with change in elevation. Many such factors might be abiotic, such as precipitation and soil type, which in turn affect many biotic factors, most obviously the plant community that butterflies depend on.
Lines 545-547. The effect of wing condition here is likely to be due to insect age. This point is discussed much later and at greater length than needed. It would be most efficient and informative to mention here.
Lines 552-555. As mentioned previously, the association interpreted here doesn’t tell the direction or nature of causality. Perhaps the species that didn’t move upslope as much have evolved higher thermoregulatory capacity. Is there evidence in this study or elsewhere that rules out this alternative interpretation? If so, this is the place to state it.
Lines 557, 562. Remove “heated up at similar rates” and “rate of heat gain” and replace with accurate language.
Lines 564-565. I suggest not invoking a forecast of average global cloud cover. It’s not very convincing. Climate change forecasts of precipitation and cloud cover patterns for any given part of the Earth at any given time of year are questionable. Weather forecasts can do this with reasonable confidence, but not climate forecasts.
Line 570. Replace “heating rate” with accurate language.
Lines 571-573. I disagree with this conclusion for reasons discussed above.
Lines 573-574. This conclusion is also unwarranted without ruling out the alternative discussed above.
Lines 586-588. I agree that the evidence is insufficient. I suggest adding that this means the evidence is inconclusive on this point, so readers do not jump to conclusions.
Line 608. Change “copes” to “cope” for grammatical agreement.
Lines 651-652. Replace “performed similarly” with a more specific statement.
Lines 671-672. It would be clearer to say that dark species showed higher thermoregulatory capacity at low elevations.
Lines 674-676. I don’t follow the reasoning here. This speculation seems to go too far beyond the evidence.
Lines 679-680. This interpretation is too strongly worded. There was no measure of performance other than the thermal tolerance assay, which is not well validated as discussed above.
Lines 682-683. This conclusion is weak because the association between wing darkness and elevation is confounded by other factors.
Lines 691-692. Can a cline be interspecific? This language seems to dismiss the many other traits and properties that differ among species.
Line 709. Replace “hotter climatic conditions (lower elevations)” with “lower elevations.” It’s important not to risk misattribution. This is one of the key points in Evans et al. 2025, Nature Climate Change.
Line 714. I’m skeptical that small butterflies would not be able to disperse elevationally, especially on the order of 1000 meters.
Lines 718-720. This statement goes too far beyond the evidence.
Lines 723-725. This statement goes too far beyond the evidence.
Lines 725-726. I’m confused. In previous passages, the argument was that small species are most vulnerable to extreme heat. This seems to be saying the opposite.
Lines 704-791. I suggest cutting out a lot of the speculative content here. The Discussion section should be shortened considerably by editing out the most speculative content here and elsewhere.
Line 797. Be more specific and precise instead of “thermal and functional traits…”
Lines 799-800. The capacity of species to adapt was not tested.
Lines 815-821. Conservation planning generally accounts for species redistribution. Focus on how this study specifically informs conservation planning in the area of this study and leave out the speculative parts. Alternatively, if speculative points are retained, it is important to state the level of confidence based on the science. Which conclusions can be extrapolated beyond the study area and why?
Line 841. I do not agree that this is a mechanistic study. While traits are identified, the processes by which these traits affect patterns are unclear.
Lines 845-846. This conclusion (now repeated multiple times) is not founded by the evidence here.
Lines 847-849. This expectation has a weak basis. It is not clear if the result underlying this conclusion is context-dependent. My skepticism stems from the fact that there are many small, dark-colored butterfly species in desert environments, which have had extreme climate for long periods of time.

Version 2:

Reviewer comments:

Reviewer #3

(Remarks to the Author)
I appreciate the author’s rebuttal comments and revisions, which have greatly improved the manuscript. During this read I became more convinced than ever that this study makes an important and impactful contribution to climate change ecology. However, I remain unconvinced that this study provides conclusive evidence for or against “adaptation in place.” As the

author states, we simply respectfully disagree on this point, which is fine with me. I recognize that my opinion on this point is in the minority among the reviewers. Nevertheless, some of my comments below relate to this point of disagreement. However, most of my comments are aimed at helping the author improve the writing.

Specific comments

1. Title. If it were up to me, I would choose a title that best captures the most conclusive and impactful result of this study, probably something like: “Butterfly species with low thermoregulation capacity show strongest redistribution patterns in the Bavarian Alps.” As I have argued previously, I do not agree that there is strong evidence for the “not adaptation” part of the title because I think the methods of evaluating this point are not strong enough to yield conclusive results.
2. Line 32. Along the same lines as my previous comment, I suggest replacing “demonstrates” with “argues” because I do not agree that the evidence demonstrates the “rather than adaptation” part of the sentence.
3. I think the Introduction is quite strong now. Nice work!
4. Line 70. Replace “their” with “its” for grammatical agreement.
5. Line 332. Remove “that” for grammatical accuracy.
6. Figures 3, 4, and 6. I suggest replacing the heading above each graph with a more descriptive title. For example, the three graphs in Fig. 3A would be clearer if the headings were: Low elevation, Intermediate elevation, High elevation. The color coding and the legend already provide the information in terms of scaled values, making the current headings redundant and less informative than they could be. In Fig. 6, I suggest adding the scaling information in the caption, as in Figs. 3 and 4.
7. Line 534. Replace “their” with “its” for grammatical agreement.
8. Figure 8 caption. Because the color version of the figure does not have a grey ribbon, I suggest saying “shaded ribbon” instead.
9. Line 567. I suggest replacing “getting” with “being” for accuracy.
10. Line 577. I suggest replacing “they” with “butterflies” for clarity.
11. Line 581. Remove the erroneous space and period.
12. Line 597. In keeping with my stance on how well this study discounts adaptation, I suggest replacing “support the hypothesis that” with “determine if.”
13. Discussion section. Some parts are written in first person singular (“I also found” in line 606), while others are written in first person plural (“we have insufficient evidence in line 596). Whichever voice is accurate should be used consistently throughout.
14. Line 618. I suggest replacing “eroded” with a more literal verb for the sake of clarity.
15. Line 767. Replace “level” with “levels” for grammatical agreement.
16. Line 779. Delete “compared” at the end of the sentence. I think this must be a typo.
17. Lines 789-791. This interpretation emphasizes the increased wing length at low elevation. Alternatively, one could emphasize the decreased wing length at high elevation. It’s not obvious from Fig. 7B which of these perspectives might be supported by the largest deviation from the mean. I would guess that higher elevation individuals tend to have reduced size because of the short growing season for larval development (which selects for smaller final size) and lower quality larval host plants (especially leaf water content), but this is merely speculation. I leave it to the author to decide if this point is actionable.
18. Line 800. The author’s addition of “extreme weather” to the slow-growth-high-mortality hypothesis is novel, as far as I know. If other authors have included this potential source of mortality, those sources should be cited here. The Benrey and Denno 1997 paper does not include consideration of any mortality factors other than natural enemies of the herbivore, which is also the intent of the original hypothesis in Clancy and Price 1987.
19. Line 802. Remove “for” because it is grammatically awkward and unnecessary.
20. Line 858. I suggest ending the sentence after “…(Evans et al. 2025)” and starting a new sentence with “Here I argue…” Alternatively, a semicolon would work if this is kept as one (very long) sentence, but a comma doesn’t work grammatically.
21. Line 860. Replace “their” with “its” for grammatical agreement.
22. Line 861. Remove “spatial” because it is redundant.
23. Line 868. Consistent with my stance expressed throughout, I suggest replacing “adaptive potential” with “variation.” It's not clear if this is adaptive potential because there may be underlying genetic or plastic capacity that was not observed here. For example, if the association between range shift and thermoregulation capacity is due to butterflies shifting their geographic ranges and thus avoiding selection on thermal traits, the adaptive potential of many species has not been tested (by the researchers or by recent natural selection from climate change).
24. Lines 870-871. Replace “butterfly communities will contain assemblages that differ” with “butterfly assemblages will differ.”
25. Line 872. The “disproportionate losses of small and dark species compared to large and pale species” is an expectation from the region studied here but not necessarily elsewhere. I suggest adding “in the Alps” at the end of this sentence.

Open Access This Peer Review File is licensed under a Creative Commons Attribution 4.0 International License, which permits use, sharing, adaptation, distribution and reproduction in any medium or format, as long as you give appropriate credit to the original author(s) and the source, provide a link to the Creative Commons license, and indicate if changes were made.
In cases where reviewers are anonymous, credit should be given to 'Anonymous Referee' and the source.
The images or other third party material in this Peer Review File are included in the article's Creative Commons license, unless indicated otherwise in a credit line to the material. If material is not included in the article's Creative Commons license and your intended use is not permitted by statutory regulation or exceeds the permitted use, you will need to obtain permission directly from the copyright holder.
To view a copy of this license, visit https://creativecommons.org/licenses/by/4.0/

- I would like to thank all the reviewers and the editor for their valuable and constructive comments. I am very grateful for the opportunity to improve my manuscript, and hope that you find the changes to be an improvement.
- Below you will find each of your comments, with my responses in bullet points beneath. I am open to any further suggestions you may have, and look forward to hearing your responses. [redacted]

Reviewer #1 (Remarks to the Author):

Summary

The manuscript "Species redistribution, not adaptation, drives butterfly responses to climate change" investigates whether butterfly thermal traits (thermoregulation and thermal tolerance) and functional traits (wing length and near-infrared reflectance) vary along an elevational gradient, and whether these traits predict recent elevational range shifts. The author report limited evidence for intraspecific adaptation in thermal traits, strong trait-dependent thermoregulation under warmer conditions, clear elevational shifts in functional composition (smaller, darker species at high elevation; larger, paler species at low elevation), and greater upward shifts in species with poorer thermoregulatory capacity.
Overall, this is a well-written manuscript that I much enjoyed reading. Experimentally the study is well-designed and very well-performed, while the analyses are relevant. The author is aware of up-to-date literature and have a deep ecological understanding and scientific knowledge with regards to the paper scope.
This study is an outstanding contribution to existing literature because it provides data on thermal traits of butterflies that are largely lacking but are key aspects of species resilience to climate change.

- I would like to thank the reviewer for the kind and encouraging words! I hope you find the changes made have improved the manuscript.

I provide below some general thoughts and minor suggestions, hoping to be of use for the improvement of the paper.

General comments
Discussion
The core findings are strong, but the Discussion is overly long and at times repetitive. Several sections restate results or explore multiple speculative mechanisms in detail. Reducing the Discussion by ~30% would improve clarity and impact.

- Thank you for raising this. The Discussion is very long and could be reduced to reduce repetition. I have reordered and rewritten the Discussion to hopefully flow better and

reduce repetition. However, I've also added a bit more text in response to the reviewer comments. I hope you find this an improvement.

Body size patterns and temperature–size expectations
The decrease in wing length with increasing elevation is interpreted in the manuscript as consistent with converse Bergmann's rule (smaller body size in colder environments). While this interpretation is well explained, it would be helpful to more explicitly acknowledge that there is also substantial literature showing that increased temperatures can drive butterflies and other ectotherms toward smaller body sizes, consistent with the Temperature-Size Rule (TSR).

- Thank you for raising this, this is a valid point. Though our data does not support the temperature-size rule (as we found butterfly communities contained more large species at lower elevations), this is still an important point to highlight. I have added text to the Introduction and Discussion (more at length) to include this and discuss this theory in the context of the results.

Repetition of research questions
What stands out is how the study questions get repeated across sections. Instead of showing up repeatedly, they could be trimmed down so each one only appears once.

- I apologise if I'm misunderstanding, but what I think you are referring to here are the subheadings? I have removed the subheadings throughout to reduce this repetition.

Minor comments
-Where multiple papers by the same first author from the same year are cited (e.g., Ashe-Jepson et al., 2023), these should be distinguished consistently as 2023a and 2023b in both the in-text citations and the reference list.

- Thank you for raising this oversight, this has been corrected.

-Please ensure consistency in notation (°C formatting; df vs d.f.).

- Thank you for raising this, this has been corrected.

-While I was reading the MS, I was thinking it would be great to have a visualization of the thermal tolerance experiment. Your description is very detailed but having, in addition to the text, that visual record, could make results feel more open and repeatable. Please consider including some photographs in the SI file.

- This is a really nice idea, I've added in a new figure (Fig S1) to the SoM that shows two photographs of the set up.

- Given that this is single author work, the author may wish to consider using singular pronouns ("I") instead of "we" for consistency.

- Yes this was something I was considering, I will change the 'we' to 'I' as it is more relevant in this case.

Reviewer #2 (Remarks to the Author):

Ashe-Jepson presents a fascinating comparative study using butterflies to suggest that species-level traits rather than changes with species are determining how organisms respond to climate change. This work combines an impressive number of field measurements across an elevational gradient in dozens of species to show that species with less physiological tolerance to heat stress are shifting to higher elevations and species traits such as coloration are influencing who can survive at different elevations. This work will be of interest to a broad range of readers, including ecologists, evolutionary biologists and environmental scientists. However, in its current form, there are several limitations.

- Thank you for your kind words!

First, throughout the paper, the text uses the word "adaptation" in a somewhat confusing manner. Many readers (myself included) would assume "adaptation" to refer to an evolutionary process, and, indeed, several references throughout the text suggest this is how it is intended. However, the method of measuring adaptation (intraspecific variation matched to local conditions) can not rule out phenotypic plasticity. If anything, I think it's more interesting that there is no evidence of variation within species along the elevational gradient due to plasticity (or adaptation) given how flexible some of these traits are developmentally. I think the phrasing needs to be adjusted throughout the manuscript to be more transparent about what was measured, for instance "intraspecific matching" which would encompass both evolutionary and developmental processes to explain the pattern of interest.

- Thank you for raising this point, it was absolutely a strong thought in my mind as well. In this case, my goal was to align the narrative with the commonly used understanding of species responses to climate change being 'disperse, adapt, or die' (e.g., https://doi.org/10.1111/j.1365-2486.2009.02014.x, in many cases genetic and plastic response both fall under the 'adaptation' umbrella term, for example see https://doi.org/10.1111/eva.12129, and I also would like to align the concepts with 'adaptive capacity', which also encompasses any response in place and is commonly used to investigate vulnerability to climate change, see https://doi.org/10.1002/fee.2253). These three categories are obviously an oversimplification, but the goal of this project was to try to determine which response butterflies were using in this case (with the focus being disperse (no trait response and instead elevational range shift), or adapt in place (show some kind of response in traits, but no elevational range shifts), as these would differentially impact conservation). I would like to remain on this focus but be clearer about exactly what is being measured (as proof of genetic changes or plasticity is not possible with the data that I have). I have rewritten the Introduction to better discuss and highlight these topics, more clearly define what is meant by 'adaptation' in this case, link to the relevant literature on this topic, and throughout the manuscript 'response' is used in place of adaptation in most cases to improve clarity. I hope you find this a useful addition.

Second, given the environmental dependence of these measures, more information is needed on the sampling conditions. The methods state "Sites were sampled in August 2025 in three two-hour periods, so that each site was visited three times and sampled for a total of six hours, to ensure even sampling effort. Sites were only visited in suitable weather, that being not raining or high wind speed conditions (above five on the Beaufort scale)." It would be useful to know the

order in which sites were sampled and how time of day and month varied with elevation since temperature likely varies with those sampling factors in consistent ways. In addition, the results lists an impressive number of individual butterflies that were sampled during 6 hours x each site (>500) – were multiple people running the captures and data collection, in which case was there any different between observers in some of the subjective measurements such as wing wear?

- It was just me out collecting butterflies, so there are no expected impacts from different observers. Luckily it was a good year with excellent weather on all but a few days and butterflies in high abundance, which meant that surveying was fruitful throughout the day, and the CTmax experiments then took place across the afternoon/evening each day. It was very long days, but I was under strict time restraints due to what the local governing authority allowed me to collect and the time I could spend in the national park. The sites were visited randomly (within reason, for example it was not possible to sample on the top of one mountain for two hours then get across the entire national park to a different mountain within a reasonable time frame, therefore sites tended to be (but not always) visited that shared mountains or at least hiking/mountain routes between them to enable fast travel (often over lunch, for example). However, the order of sites was always randomised, and extreme care was taken to ensure that all sites were visited in similar weather conditions. Much careful consideration was taken to visit all sites in comparable weather conditions (e.g. making sure each site was visited on both a hot day and a cold day at least (cold being just less hot, it was peak summer after all)). I have added a table to the supplementary materials that lists the sites and dates they were visited on to improve clarity. I hope you find this an improvement.
- If it would be of interest, I did a robustness check on the results to make sure that local weather conditions did not alter the results (which they did not, I recorded percentage cloud cover and wind speed continuously while surveying). I have not included this in the manuscript, but I am open to including this check if you feel it would improve the manuscript.

Third, several of the most interesting comparisons in the study rely on comparisons across the species. However, it does not appear that any attempt was made to correct for phylogenetic relationships across species. (or perhaps I missed that) This is concerning because phylogeny does seem to be closely tied to coloration for the subset of species sampled, for instance most of the dark butterflies are from two clades of grass-feeding butterflies (Satyrinae and Hesperiinae) and most of the light butterflies are from a different group (Pierinae and two Lycaenids). I wonder how much the statistical confidence of these comparative analyses would change if relatedness was accounted for.

- Thank you for raising this, this is important to control for. As such, I have added in tests for phylogenetic signals in the thermal traits themselves, and also re-run the models with phylogeny included and tested whether they improved the models or changed interpretation of the results. This has been added to the Methods. Please also note that colouration in this case in near-infrared reflectance, which is not in the visual spectrum. Though some dark butterflies do indeed absorb a lot of NIR, many patterns are less obvious (e.g. the most absorbent species (lowest reflectance) in this study was *Lysandra coridon*, which is pale blue in the visual spectrum, similarly *Argynnis paphia* has a comparatively high reflection score across the species tested, but seems quite dark to our vision).

Finally, several of the primary findings in the results are presented as plots that do not include any of the raw data (Figure 2 and 3, with 4 panels each). While I understand the figure looks much cleaner to just present the model output, it is much more useful (and transparent) to include data points that reflect the measurements that generated that model output. Such points are reflected in some of the supplemental figures, which are quite useful. I would suggest including them in the plots in the main figures as well so it is easier for the reader to understand patterns of underlying variation across species and sites.

- Thank you for raising this point. It is important to me that the data is presented as clearly as possible. I initially removed the data points as in the larger faceted plots they became quite overcrowded with three lines and three colours of points per facet! But I have added the points back in to more clearly present the data, and lowered the opacity so that hopefully the lines are still clear. I hope this is an improvement.

A more minor note, there are a few wording things related to temperature changes and temperature knock-down that are confusing. The methods suggest that the temperature known-down experiment in the water bath had a rising temperature until the individual butterfly was unresponsive. But then the butterfly was released. Later, the analyses, and wording in the figure legends ("lethal temperature") related to a survival analysis suggests the individuals died. Or perhaps this is a survival analysis applied to temporary knockdown? If the latter, clarification in the figure legend would be helpful. If the former, clarifying which butterflies were released in the methods would be helpful.

- Thank you for this point. For the knockdown experiment, immediately after the butterfly was knocked down it was removed and placed into a recovery cage. It was allowed to recover for a few hours, marked, and then returned and released in the site they were caught in. To make this clearer, this has been added to the Methods. The 'lethal' word you are referring to is the LT50, a commonly used phrase derived from LD50 (lethal dose 50; the dose at which 50% of participants have died, this is commonly used in the medical field but also in ecology, see https://doi.org/10.1017/S1742758400011097) which is used in time-to-event survival curve analysis. In this case, we use lethal temperature 50, representing the temperature at which 50% of individuals had fallen (e.g., https://doi.org/10.1111/j.1365-2435.2011.01908.x). This value is purely to aid in interpretation between the slopes, as otherwise it becomes quite wordy and descriptive! To be clearer, I could change this to a new name, something like 'fall temperature 50', but this would require defining. In the meantime, I have added the definition to the figure legends, I hope this improves clarity.
- Something to note in this case, the upper thermal limits I am reporting are not lethal limits. They refer to the temperatures at which the insect can no longer function (ecologically 'dead' – can't escape further heat, avoid predators, find mates/egg laying sites, essentially no longer functioning within the ecosystem). Without the use of a respirometer (very expensive) or static assays (which require large sample sizes and take a lot of time), it is not possible to accurately quantify the point at which insects die under heat stress. Ramping assays therefore focus on temperatures at which the insects can no longer function (as insect muscles can only function within certain temperature limits). This is a recoverable state, after their body temperatures reduce, they return to normal behaviour and resume coordinated movement.

- I am open to changing the terminology here if it is confusing, it is important to me that I am very clear in my wording, but I am using terms commonly used in this field, and terms I have used in previous publications (see https://doi.org/10.1111/1365-2656.13970 where I used LD50 but now feel that is not representative of what we are doing here). I am happy to follow your guidance on this.

On a related note, several parts of the results discuss differences across butterflies in "heating rates" and "heating up." This is confusing because as far as I can tell, these results apply to the differences across individuals in body temperature and "rate" applies to the differences across individuals captured in different temperatures, and the only part of the experiment that directly measured heating rates was the CTmax experiment where butterfly temperatures were not measured, instead their response to rising temperatures. I suggest choosing more clear and consistent language and/or adding a figure to clarify the two approaches to measuring responses to temperature.

- Yes I understand how confusing all these similar terms can be! In the past I have used terms such as 'buffering ability', but over the last few years I find that this term is confusing to most people who are not already familiar with this field of research, it is not intuitive in what it means, and especially with non-native speakers it requires some explanation. As such, I have started to move towards more intuitive language and try to use terms like 'heating rate' when specifically referring to the rate at which species gain heat (i.e. the relationship between body temperature and air temperature, the slope is a numerical value of how much one unit of body temperature changes per unit increase in air temperature, usually discussed as a species-level trait as you cannot infer this from a single individual), or something like 'thermoregulatory capacity', which still requires some explanation but at least it is a bit more intuitive. I find language such as heating up, heating rate, are intuitive and relatively easy to understand, and these are only ever used in relation to body temperature, never for the CTmax. The CTmax experiment did not measure heating rate, it measured response to high temperature (as I myself controlled the heating rate by setting the ramping rate). I hope this is more clear. I am of course open to changing my terminology, and have replaced 'heating rate' with 'thermoregulatory capacity' throughout the manuscript which I hope is clearer. I like the idea of including a conceptual figure but I'm not sure exactly what that would entail. I have a simplified conceptual figure (below) I could include that demonstrates what I would expect the 'suitable' response of either trait to look like across the elevational gradient. Perhaps you could indicate whether you think something like this could be a suitable addition to the manuscript (it is quite a simplification, but hopefully it gets the point across).

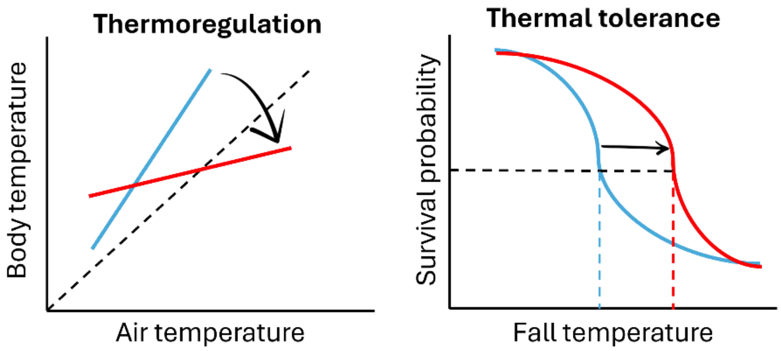

Reviewer #3 (Remarks to the Author):

This manuscript reports a space-for-time-substitution study of butterfly thermal and other functional traits along an elevational gradient in the Austrian Alps to determine how butterflies are responding to climate change. The two general types of butterfly responses considered are trait adaptation and trait-dependent changes in geographic distribution. That intraspecific variation in thermal traits was not associated with the elevational gradient was interpreted as evidence against trait adaptation. By contrast, combinations of thermal and other functional traits showed complex patterns of variation over the elevational gradient. Most importantly, one thermal trait (thermoregulatory capacity) and one other functional trait (wing length) were associated with previously reported elevational changes in butterfly species from the same region. The authors conclude that such traits are driving geographic changes in individual species as the main responses to climate change.

This study complements other literature, especially Bladon et al. (2020), which shows that in UK butterflies “the effectiveness of behavioural thermoregulation compared to microclimate selection predicted species’ long-term population trends, with those relying more on microclimate selection experiencing more negative population trends over the last 40 years.” In the present manuscript, the authors show that the same thermoregulation trait predicts “elevational range shift, whereby species with poorer ability to thermoregulate (low thermoregulatory capacity) showed larger upwards elevational range shifts.” Taken together, the evidence suggests that European butterfly species possessing high thermoregulatory capacity are more resistant to climate warming than species with low thermoregulatory capacity. This and other recent studies are thus identifying specific traits that determine species responses to climate change. To me, this is the strongest takeaway from this study. I see this as an important contribution to our understanding of how species in natural ecological communities are responding to climate change.

- Thank you for your kind comments on the manuscript. I hope I have addressed your concerns below.

However, I also have critiques about the methodology and interpretation of the results, leaving me unconvinced by some of the other conclusions drawn. I think the authors should address the following.

1. The space-for-time-substitution (SFTS) approach is questionable for the forecasting interpretations. As the authors point out in the Discussion section, a recent commentary piece

(Evans et al. 2025, Nature Climate Change) critiques the SFTS approach in research that forecasts ecological consequences of climate change and suggests alternative approaches. The two main problems with SFTS are: 1) misattributed causality of climatic variables that are spatially correlated with ecological response variables, and 2) failure to distinguish transient from equilibrial ecological responses to climate change. The trait of wing length is questionable because there is no strong evidence for a causal link between climate and the trait. Perhaps there is such a link, but it also might be spatially correlated with ecological response variables to climate change. If I am perhaps overlooking the causal evidence, then I suggest the authors put greater emphasis on this point. Other traits (thermoregulation, thermal tolerance, wing coloration) that are more clearly relevant may also be correlated with other traits (e.g., behavior, temporal/spatial activity) that causally respond to climate change. A more nuanced issue with these traits is how they are causally related to weather versus climate. This distinction is not explicitly considered in this manuscript. For example, what is meant exactly by "extreme heat" in the conclusion: "This implies that small and dark species will be particularly vulnerable to extreme heat" (Abstract). If the thermal traits are most relevant to weather, they would function to help individual butterflies deal with the thermal variation they experience (i.e. acclimation). A critical question is how phenotypically plastic these traits are. If the traits are relevant to climate, variation in such traits would be associated with variation in climate over decades. The analysis addressing question 3 in the manuscript is the most convincing one because it shows such an association between thermoregulatory capacity and decadal elevation shifts.

- Thank you for raising this important point. Firstly, in butterflies there are several papers that look at wing length and thermoregulatory capacity and thermal tolerance, see: https://doi.org/10.1111/1365-2656.13319, https://doi.org/10.1111/1365-2656.13970, https://doi.org/10.1111/1365-2656.14039), showing that this trait is influential with the ways that butterflies interact with environmental temperature. The expected causal impact of wing length is how it correlates strongly with body size in butterflies, and therefore will impact the thermodynamics of the organism. In butterflies, wing length also acts as a determinant of flight capacity/dispersal ability. In that case, it will impact how fast and efficiently a butterfly can search an area for a suitable microclimate. It may also impact which sorts of microclimates a butterfly is inclined to use, though that so far as never been tested. Both size and colouration were chosen because of their expected impacts on the thermodynamics of the insects, and the previous evidence collected in butterflies that demonstrates that these particular traits are important.
- To address your comment of what is meant by 'extreme heat', in this case 'extreme heat' is extreme weather events, such as heatwaves which are increasingly common in the region (in fact, there was a heatwave in the alpine study region sadly before I started sampling (but was setting up my study sites, this was in June-July), where I recorded air temperatures up to 30C in my highest site (approx. 1800 m). Minor changes in mean air temperature are unlikely to elicit strong responses from my study species, particularly as they are likely to benefit butterfly activity rather than hinder it (considering their relatively high thermal requirement in their flight muscles for active flight). Instead, there is growing evidence that temperature extremes (such as the horrible heatwave that hit Europe that summer, where some parts of Europe nearly reached 50C) will be bigger drivers of responses in butterflies than changes in mean temperatures (e.g., https://doi.org/10.1007/s10144-013-0409-y). Therefore, when discussing butterfly vulnerability to climate change, the importance of thermoregulation or thermal tolerance, I tend to focus more on extreme conditions than gradual changes to means, particularly in these relatively cool regions.

- In relation to your comment about weather, firstly it is of note that my interest in this manuscript is understanding whether species essentially customise their thermal traits to suit local conditions (air temperature) across climates (elevation) (e.g., it would be expected that in a hot climate, having better heat avoidance strategies should be selected for, or higher upper thermal limits, or functional traits that correspond to those thermal traits). Something of note, throughout the surveying period I continuously recorded percentage cloud cover and wind speed, and I did a robustness check to compare my thermoregulatory model with and without weather variables (e.g. does local weather impact these results, does controlling for the effect of weather impact the results) and found that they did not improve the model nor did they impact thermoregulatory capacity. I personally feel that this is probably the result of air temperature already accounting for all the variation in weather, as this was recorded for every individual in the location they were found, rather than taking daily means for example. If you would like, I have left this robustness check in my analysis code which I will make available with the manuscript should it be accepted, but I could include this in the Methods if you feel this would be valuable. I am conscious of the length of the Methods now that I have added in all the other robustness checks. Perhaps I could move all of these to the supplementary where I could go into more detail there without adding excessive length to the manuscript. I am open to your suggestions.

2. Thermoregulatory capacity is misleadingly called “heating rate” in some places (Abstract, Data analysis part of Methods, Results section). This trait is quantified in linear mixed effects models as the intraspecific variation in butterfly body temperature as a function of air temperature where each butterfly was captured. It is not a rate. The linear slope of this regression has previously been called “temperature buffering” (e.g., Bladon et al. 2020), with shallow slopes indicating relatively high temperature buffering capacity and steeper slopes indicating the opposite. I suggest the authors go back to “temperature buffering” or consistently use “thermoregulatory capacity,” which is also sometimes used in the present manuscript. But many statements are misleading, such as “Dark species had slower heating rates” (Abstract), and these should be removed.

- Thermoregulatory capacity in this sense is the slope between body temperature and air temperature, so this is a numerical value that corresponds to how much one unit of body temperature changes with a unit increase in air temperature, which I would define as a rate. However, I agree that terminology in this case can be very confusing! I have personally published work on this metric using terms such as ‘buffering ability’ and the like, and find that it is not intuitive and requires quite a lot of explanation, particularly now that I work in a country where English is not the native language. Therefore I do not agree with the use of ‘buffering ability’ anymore and have been moving towards a more intuitive term and have more commonly been using ‘thermoregulatory capacity’ (for example see here where I use both: https://doi.org/10.1002/ece3.10623), as though this is also not perfectly intuitive, it is at least clear that this is related to how good something is at thermoregulating. In my experience of sharing my research with other people both inside and out of my field, this is easier to explain. However, in this case I was curious whether it was clearer to use heating rate rather than thermoregulatory capacity when referring only to the slope of the relationship (as previously it is unclear whether this also includes baseline body temperature). As this is obviously not clear, I have changed the text to ‘thermoregulatory capacity’ throughout when discussing the

slope and removed mention of heating rates in all cases except where I am explicitly talking about rates of heat gain and thermodynamics.

3. The thermal tolerance trait is of questionable ecological relevance without further validation. If a butterfly approaches its thermal tolerance in the wild, perhaps it will probably find a shady place to rest and cool down. Many desert butterflies, for example, restrict their flight activity to early morning hours during the summer and spend the heat of the day resting in shady places. The desert butterflies are already adapted to extreme climate and extreme weather. Without further study of trait plasticity and how it relates to other functional traits (e.g., behavior), one cannot know how the thermal tolerance measures of butterfly species in the present study would predict their responses to a heat wave (weather) or climate change. It is reasonable to hypothesize that higher thermal tolerance by this measure would benefit butterflies subjected to either hot weather or a warming climate. But heat avoidance behaviors of the butterflies might predominate, rendering thermal tolerance less relevant. In sum, I don't think there is a strong enough basis for the prediction "that butterflies will not be able to compensate for increased body temperature by raising their thermal tolerance."

- Thank you for raising this. I acknowledge that of course behavioural thermoregulation alters the experienced body temperatures of butterflies (I include thermoregulation capacity as a focus point of the paper) and therefore alters the ecological relevance of upper thermal limits. Similarly, thermoregulatory capacity lacks context without upper thermal limits, which is why I felt both metrics interesting and complementary to include. However, physiological thermal limits are still widely considered an important component of species responses to warming (particularly to extreme heat rather than to gradual warming) as they define the upper boundary condition beyond which behavioural thermoregulation is no longer sufficient (for a recent Nature publication that used upper thermal limits to investigate insect vulnerability to climate change see https://doi.org/10.1038/s41586-026-10155-w). During extreme heat events, behavioural thermoregulation in small insects may not be able to buffer individuals from experiencing extreme body temperatures that approach their physiological limits (e.g., https://doi.org/10.1073/pnas.1316145111, also note this study that recorded butterfly body temperatures during an extreme heatwave in the UK and note that very few datapoints are lower than ambient, and those that are are very close to 1:1 showing thermoconformance rather than an ability to lower body temperature below ambient: https://doi.org/10.1007/s10841-024-00556-5). Previous work of mine has demonstrated a trade-off between thermoregulatory capacity and upper thermal limits in butterflies (see https://doi.org/10.1111/1365-2656.13970), highlighting that in some cases these traits can compensate for each other, which is where the choice of language comes from. I have altered the text however to remove this statement, as it feels too strong given the evidence.
- To be more specific on this given system, this study took place in open grasslands with little shade or shelter, therefore limiting heat avoidance. Activity would innately expose these insects to heat, so in this case, microhabitats may not be able to buffer species from extreme heat during heatwaves. In addition, landscapes around the world are being homogenised, and therefore also thermally homogenised, risking disruption to processes such as behavioural thermoregulation in small insects, therefore this lack of response in upper thermal limits to elevation may be of wider interest. Though behavioural thermoregulation can buffer heat exposure, physiological tolerance remains

a relevant trait to investigate and compare small ectotherm vulnerability under extreme conditions. Again, to date there is no evidence that butterflies can lower their body temperature below ambient conditions, so should ambient conditions reach physiological limits, behavioural thermoregulation may not be able to buffer this (for example the heatwave that hit Europe in 2025 had air temperatures nearing 50C, above the upper thermal limits of every species tested, unfortunately these heatwave temperatures are increasingly common and extreme). I have added text and references to the Discussion to better highlight this and highlight the role and limitations of upper thermal limits when considering species vulnerability to climate change.

4. The limited sampling of many butterfly species along the elevational gradient (1000 meters) offers only a weak test of the possibility that the butterflies are responding to climate change via trait adaptation. As such, lack of evidence does not equate to evidence of absence. In Table S2, the sample size of each species per site is not shown. However, with one exception (Erebia aethiops, N = 169 total), the sample size per species per site must be quite small. Aside from this outlier, the larger sample sizes range from 30-61 across 9 sites. It is not reasonable to expect to detect a signature of local adaptation based on 3-7 individuals per species per site. While the analyses are based on traits, which are shared among multiple species and thus may allow the authors to circumvent the sample size issue to some degree, there was no attempt to account for non-independence of traits shared by closely related species (i.e. no phylogenetic comparative approach). Again, it would be valuable to know how phenotypically plastic these thermal traits are. Otherwise, it is difficult to understand their function in relation to environmental variation as well as in relation to other co-functional traits. I see no strong basis for the conclusion "some sort of constraint or limit has been reached" in the evolution of thermoregulatory capacity. Likewise, I disagree with the assertion that this study "demonstrates that butterflies are responding to climate change through redistribution of species rather than adaptation in place." While this study and many before it show evidence for redistribution of species, the evidence here on adaptation in place is inconclusive.

- Thank you for raising this valid concern. Firstly, I want to address your point about the sample size. This study took place within a national park in Germany, where all butterflies are protected by law (and especially in such areas). This national park is also the only part of the Alps in Germany, so other research sites were not accessible for this project. To conduct this research project, permits had to be acquired that imposed limits on numbers of butterflies that could be caught within a strict time limit that was set by the local governing authority. As such, unfortunately it is not possible to increase the sample size further, though I acknowledge that smaller sample sizes reduce statistical power to detect responses. Unfortunately, sample size limitations are very common in field studies of wild populations, particularly on protected species in protected areas.
- I want to explain how the analysis should have helped to control for this. Species was always included as a random effect, rare species should therefore contribute to the overall relationships, but not get much weight in estimating their species-specific intercept. However, I feel this is important to empirically test. I have added a series of checks in the analysis to test for the impact of species-specific sample size on the results (i.e. were rare species obscuring patterns visible in the more abundant species). First, I remove all rare species (sample size < 10) and refit the models. This showed no

difference in coefficient estimates strength or direction. For a stronger check, I then did a k-fold cross-validation, comparing how the model predictions were impacted by sample size (again comparing the full dataset to only species with >10 observations), here again coefficients were consistent in strength and direction, and the full model predicted slightly better. I checked whether the differences between the models were because of just sample size, or whether the rare species themselves were contributing something meaningful, so I ran another k-fold CV comparing a model trained on only the most abundant species against a random subsample of the abundant species but at the same sample size as the full dataset. These two performed very similarly, and both outperformed the subset model, indicating that sample size marginally improved the model predictive power, and the inclusion of rare species did not alter the performance. I did something similar for the Cox models, where I used the concordance index instead, and again found very little difference between the models (difference in C-statistic of 0.002). I have added this to the Methods section for both analyses as a robustness check, and it can be found in the associated code. These tests can only tell me whether or not the sample size per species is impacting the models, I cannot explicitly test for whether more data (that I don't have) might change the results. However, one thing I can do is as you mention, *E. aethiops* has a high sample size across the gradient. I have tested this species in isolation for both analyses, and the results are consistent with the full models (no change in thermoregulatory capacity across the gradient; no change in upper thermal limits across the gradient). If it would be valuable, I could include this in the manuscript, at present it is only present in the code (which will be published alongside the manuscript). I am open to your suggestion on this.

- I have also included phylogenetic sensitivity checks for each analysis (comparing non-phylogenetic models with phylogenetic models, and testing for phylogenetic signals in the response variables), which have been included in the Methods. Something of note is that the results found in this project align with the existing literature, where no difference in thermoregulatory capacity has been found across much wider geographic comparisons (e.g. comparing same species in UK and Spain: https://doi.org/10.1111/1365-2656.14039 and UK and Panama (https://doi.org/10.1111/gcb.16797). However I feel it is important to be upfront with the reader about the sample sizes, so I have added a caveat to the Discussion to highlight that larger sample sizes may allow for greater possibility of detection. Please note that the vast majority of species were not found across the entire gradient (with a few notable exceptions such as *E. aethiops* and interestingly *L. coridon*, and a few others).
- In this case I found no evidence for consistent shifts in thermal traits across the gradient within this study system, which goes against my hypothesis of species optimising these traits in suit their local climate. Instead it appears that these are intrinsic properties of species with limited capacity to change, at least within the given system. However, we did detect strong interspecific differences according to size and colour, which aligns with the later results of species redistributions (tracking the climate rather than adapting in place), as well as historic trends from paleoecology of insects generally redistributing rather than adapting in place. However, I want to make it clear to the reader that in this case, we cannot exclude the possibility that there is something occurring here, but that we just have insufficient evidence to suggest that species do not adjust their thermoregulatory capacity (similar to all other studies on butterflies), but that a more detailed analysis with a higher sampling intensity may detect different patterns. I have

added text to the Discussion to highlight this important caveat, and thank you for raising this concern.

- **I would like to thank all reviewers for taking the time to provide further feedback on my manuscript, I am very grateful for the opportunity to improve under their guidance. Please note that any references to line numbers refer to the version with tracked changes (so that you can see exactly what has changed). I have also bolded all new comments as well as my responses to them (which are bullet points underneath).**

Reviewers' comments:

Reviewer #1 (Remarks to the Author):

I appreciate the extent of revisions made by the author. I am content with the revisions made and am happy to recommend the manuscript be published.

- **Thank you for your valuable comments on the earlier drafts of this manuscript, I appreciate the opportunity to improve it under your suggestions.**

Reviewer #2 (Remarks to the Author):

The author has done an incredibly job addressing the initial round of reviews. Not only have extensive changes been made to the introduction to clarify terms and phrasing, but there are also new analyses to control for phylogeny and updated figures to show individual data points. New data on weather differences across sites reassures the reader that variation in weather on sampling days is not confounding the findings. I am very impressed by the breadth and depth of the revisions. This addresses all of my concerns in terms of alternate explanations and interpretations. This research will make a big impact on the field.

- **Thank you for your insightful comments, I am grateful for the opportunity to be able to improve my manuscript from the suggestions I have received.**

Reviewer #3 (Remarks to the Author):

To further the conversation, I have pasted my previous comments along with the author's rebuttal comments before my new round of responses (see each "New reviewer response"). I also added line-specific comments to make my critical comments more detailed and constructive.

- **Thank you for making this so clear, I have added my responses to your new comments underneath and bolded them to make it clear which response are new. I also appreciate the detailed line-specific comments.**

This manuscript reports a space-for-time-substitution study of butterfly thermal and other functional traits along an elevational gradient in the Austrian Alps to determine how butterflies are responding to climate change. The two general types of butterfly responses considered are trait adaptation and trait-dependent changes in geographic distribution. That intraspecific variation in thermal traits was not associated with the elevational gradient was interpreted as evidence against trait adaptation. By contrast, combinations of thermal and other functional traits showed complex patterns of variation over the elevational gradient. Most importantly, one thermal trait (thermoregulatory capacity) and one other functional trait (wing length) were associated with previously reported elevational changes in butterfly species from the same region. The authors conclude that such traits are driving geographic changes in individual species as the main responses to climate change. This study complements other literature, especially Bladon et al. (2020), which shows that in UK butterflies "the effectiveness of behavioural thermoregulation compared to microclimate selection predicted species' long-term population trends, with those relying more on microclimate selection experiencing more negative population trends over the last 40 years." In the present manuscript, the authors show that the same thermoregulation trait predicts "elevational range shift, whereby species with poorer ability to thermoregulate (low thermoregulatory capacity) showed larger upwards elevational range shifts." Taken together, the evidence suggests that European butterfly species possessing high thermoregulatory capacity are more resistant to climate warming than species with low thermoregulatory capacity. This and other recent studies are thus identifying specific traits that determine species responses to climate change. To me, this is the strongest takeaway from this study. I see this as an important contribution to our understanding of how species in natural ecological communities are responding to climate change.
• Thank you for your kind comments on the manuscript. I hope I have addressed your concerns below.

However, I also have critiques about the methodology and interpretation of the results, leaving me unconvinced by some of the other conclusions drawn. I think the authors should address the following. 1. The space-for-time-substitution (SFTS) approach is questionable for the forecasting interpretations. As the authors point out in the Discussion section, a recent commentary piece (Evans et al. 2025, Nature Climate Change) critiques the SFTS approach in research that forecasts ecological consequences of climate change and suggests alternative approaches. The two main problems with SFTS are: 1) misattributed causality of climatic variables that are spatially correlated with ecological response variables, and 2) failure to distinguish transient from equilibrial ecological responses to climate change. The trait of wing length is questionable because there is no strong evidence for a causal link between climate and the trait. Perhaps there is such a link, but it also might be spatially correlated with ecological response variables to climate change. If I am perhaps overlooking the causal evidence, then I suggest the authors put greater emphasis on this point. Other traits (thermoregulation, thermal tolerance, wing coloration) that are more clearly relevant may also be correlated with other traits (e.g., behavior, temporal/spatial activity) that causally respond to climate change. A more nuanced issue with these traits is how they are causally related to weather versus climate. This distinction is not explicitly considered in this manuscript. For example, what is meant exactly by "extreme heat" in the conclusion: "This implies that small and dark species will be particularly vulnerable to extreme heat" (Abstract). If the thermal traits are most relevant to weather, they would function to help individual butterflies deal with the thermal variation they experience (i.e. acclimation). A critical question is how phenotypically plastic these traits are. If the traits are relevant to climate, variation in such traits would be

associated with variation in climate over decades. The analysis addressing question 3 in the manuscript is the most convincing one because it shows such an association between thermoregulatory capacity and decadal elevation shifts.

• Thank you for raising this important point. Firstly, in butterflies there are several papers that look at wing length and thermoregulatory capacity and thermal tolerance, see: https://doi.org/10.1111/1365-2656.13319, https://doi.org/10.1111/1365-2656.13970, https://doi.org/10.1111/1365-2656.14039), showing that this trait is influential with the ways that butterflies interact with environmental temperature. The expected causal impact of wing length is how it correlates strongly with body size in butterflies, and therefore will impact the thermodynamics of the organism. In butterflies, wing length also acts as a determinant of flight capacity/dispersal ability. In that case, it will impact how fast and efficiently a butterfly can search an area for a suitable microclimate. It may also impact which sorts of microclimates a butterfly is inclined to use, though that so far as never been tested. Both size and colouration were chosen because of their expected impacts on the thermodynamics of the insects, and the previous evidence collected in butterflies that demonstrates that these particular traits are important.

New reviewer comment:
I am satisfied with the author's response on this point.

- **Thank you.**

• To address your comment of what is meant by 'extreme heat', in this case 'extreme heat' is extreme weather events, such as heatwaves which are increasingly common in the region (in fact, there was a heatwave in the alpine study region sadly before I started sampling (but was setting up my study sites, this was in June-July), where I recorded air temperatures up to 30C in my highest site (approx. 1800 m). Minor changes in mean air temperature are unlikely to elicit strong responses from my study species, particularly as they are likely to benefit butterfly activity rather than hinder it (considering their relatively high thermal requirement in their flight muscles for active flight). Instead, there is growing evidence that temperature extremes (such as the horrible heatwave that hit Europe that summer, where some parts of Europe nearly reached 50C) will be bigger drivers of responses in butterflies than changes in mean temperatures (e.g., https://doi.org/10.1007/s10144-013-0409-y). Therefore, when discussing butterfly vulnerability to climate change, the importance of thermoregulation or thermal tolerance, I tend to focus more on extreme conditions than gradual changes to means, particularly in these relatively cool regions.

New reviewer comment:
The author's response makes sense to me. As long as this is clear in the revised manuscript (and I believe it is clear now), I am satisfied.

- **Thank you.**

• In relation to your comment about weather, firstly it is of note that my interest in this

manuscript is understanding whether species essentially customise their thermal traits to suit local conditions (air temperature) across climates (elevation) (e.g., it would be expected that in a hot climate, having better heat avoidance strategies should be selected for, or higher upper thermal limits, or functional traits that correspond to those thermal traits). Something of note, throughout the surveying period I continuously recorded percentage cloud cover and wind speed, and I did a robustness check to compare my thermoregulatory model with and without weather variables (e.g. does local weather impact these results, does controlling for the effect of weather impact the results) and found that they did not improve the model nor did they impact thermoregulatory capacity. I personally feel that this is probably the result of air temperature already accounting for all the variation in weather, as this was recorded for every individual in the location they were found, rather than taking daily means for example. If you would like, I have left this robustness check in my analysis code which I will make available with the manuscript should it be accepted, but I could include this in the Methods if you feel this would be valuable. I am conscious of the length of the Methods now that I have added in all the other robustness checks. Perhaps I could move all of these to the supplementary where I could go into more detail there without adding excessive length to the manuscript. I am open to your suggestions.

New reviewer comment:
Thanks for the clarification. I'm less concerned about the effects of weather conditions during the study than I am about interpretation of the thermal traits and their relationship to air temperature and elevation. In general, I find the thermoregulation trait most convincing because the relationship between body temperature and air temperature is clearly meaningful. However, without knowledge of the plasticity of this relationship (i.e. plasticity of thermoregulation), it is difficult to interpret the functional significance of this thermoregulation trait. For example, how much can this relationship vary within a species? Would temperatures or other cues earlier in development change it? I don't think this is known for any butterfly species, but if it is known, this information would be important to discuss. Another challenge is the causal relationship between the observed elevational range shift and this thermoregulation trait across butterfly species. The author hypothesizes "that butterflies with a poorer ability to thermoregulate and with low flexibility in this trait will have shown stronger responses to recent climate change, so greater upwards elevational range shifts, as they should be less able to cope with changes to their local climatic conditions." While I agree that this is a good hypothesis, it is also plausible that butterfly species with greater upwards elevational range shifts have been able to maintain their climate envelopes, thus shielding them from recent selection on thermoregulation traits. This alternative hypothesis proposes that the observed variation in thermoregulation capacity is a consequence of the strength of selection on this trait, which could be genetically fixed or close to fixed within each species. If this is true, it would result in the patterns observed in this study.

- **Thank you for this valuable comment. I very much agree that understanding the plasticity of this trait would really help in interpretation! I will answer your questions one at a time. Firstly, in regards to whether it varies within species, that is the purpose of this paper, to investigate how much this trait can vary intraspecifically across the gradient. To date there is no studies have presented evidence that this trait changes within species, though it has only ever been formally tested between discrete regions, never along a gradient (see here for UK**

and Spain comparison of same species: https://doi.org/10.1111/1365-2656.14039, only very minor differences in a minority of species which may also be the consequence of the slightly different methodologies between regions (i.e., different amounts of time waited between capture and body temperature recordings). Otherwise, if you look at other studies that present their data at the species level (for example: https://doi.org/10.1111/1365-2656.13319 Figure 1, https://doi.org/10.1111/1365-2656.13970 Figures S1-6), you can see how much variation there is in this body temperature – air temperature relationship within species, notably not much! Note that this is also available in Figure S2 for the data collected in this study.

- **In relation to your question about the impact of developmental conditions, this is a very interesting question, this could be tested for if we had data across multiple years, or knew exactly where the butterflies developed as larvae. In this case, note that all data was collected in a single month, so there is no expected impact of inter-year differences within species, and as wild-caught butterflies it is impossible to know exactly where they developed, these are highly mobile animals. However to date, the only papers I know of that have used data across multiple years include the Bladon et al 2020 paper, which did not test for differences between years (to my knowledge). So at present, this is just not known.**
- **As for your final point about the causal link between thermoregulatory capacity and the elevational range shifts, thank you for raising this. Yes you are absolutely right that the exact causal link is unknown, I have presented only a single theory, but there may be other reasons why we observe these patterns. To highlight these alternative explanations, I have added text to the Discussion (L829-835). Thank you for raising this.**

2. Thermoregulatory capacity is misleadingly called "heating rate" in some places (Abstract, Data analysis part of Methods, Results section). This trait is quantified in linear mixed effects models as the intraspecific variation in butterfly body temperature as a function of air temperature where each butterfly was captured. It is not a rate. The linear slope of this regression has previously been called "temperature buffering" (e.g., Bladon et al. 2020), with shallow slopes indicating relatively high temperature buffering capacity and steeper slopes indicating the opposite. I suggest the authors go back to "temperature buffering" or consistently use "thermoregulatory capacity," which is also sometimes used in the present manuscript. But many statements are misleading, such as "Dark species had slower heating rates" (Abstract), and these should be removed.

• Thermoregulatory capacity in this sense is the slope between body temperature and air temperature, so this is a numerical value that corresponds to how much one unit of body temperature changes with a unit increase in air temperature, which I would define as a rate. However, I agree that terminology in this case can be very confusing! I have personally published work on this metric using terms such as 'buffering ability' and the like, and find that it is not intuitive and requires quite a lot of explanation, particularly now that I work in a country where English is not the native language. Therefore I do not agree with the use of 'buffering ability' anymore and have been moving towards a more intuitive term and have more commonly been using 'thermoregulatory capacity' (for example see here where I use both:

https://doi.org/10.1002/ece3.10623), as though this is also not perfectly intuitive, it is at least clear that this is related to how good something is at thermoregulating. In my experience of sharing my research with other people both inside and out of my field, this is easier to explain. However, in this case I was curious whether it was clearer to use heating rate rather than thermoregulatory capacity when referring only to the slope of the relationship (as previously it is unclear whether this also includes baseline body temperature). As this is obviously not clear, I have changed the text to 'thermoregulatory capacity' throughout when discussing the slope and removed mention of heating rates in all cases except where I am explicitly talking about rates of heat gain and thermodynamics.

New reviewer comment:
This metric of thermoregulatory capacity cannot tell you the rate of heating up in individual butterflies. It is a species-level trait that might indicate that individual butterflies heat up more or less rapidly as the environmental temperature increases. But it might alternatively reflect behavioral thermoregulation traits that enable individual butterflies to maintain a particular body temperature by heating or cooling despite cooler or warmer environmental temperature. Such behavioral traits include basking in sun, resting in shade, spending time in specific microclimates (vegetation, on the ground, etc.). Therefore, I continue to insist that "heating up rate" is not justified as a shorthand description of this metric, and I recommend removing this language throughout the manuscript.

- **I think this might be a language issue, I think we have different interpretations of what 'heating rate' means. No individual was ever re-recorded (not has ever been re-recorded in any study to my knowledge on butterfly body temperatures in wild populations), so of course this is not about heating rates of individuals, but rather the overall pattern of how the rate of body temperature change differs with changing in air temperature (i.e., how fast a species heats up in hot weather). This language is consistent with other literature on the subject that recorded butterfly body temperatures against air temperature (e.g., https://doi.org/10.1016/j.jtherbio.2014.02.002, https://doi.org/10.1111/gcb.16797). However to improve clarity, 'heating rate' has been removed from the manuscript. I will not use 'buffering ability' as it is too unclear, therefore I insist on using thermoregulatory capacity, as explained in the previous round of reviewer comments.**

3. The thermal tolerance trait is of questionable ecological relevance without further validation. If a butterfly approaches its thermal tolerance in the wild, perhaps it will probably find a shady place to rest and cool down. Many desert butterflies, for example, restrict their flight activity to early morning hours during the summer and spend the heat of the day resting in shady places. The desert butterflies are already adapted to extreme climate and extreme weather. Without further study of trait plasticity and how it relates to other functional traits (e.g., behavior), one cannot know how the thermal tolerance measures of butterfly species in the present study would predict their responses to a heat wave (weather) or climate change. It is reasonable to hypothesize that higher thermal tolerance by this measure would benefit butterflies subjected to either hot weather or a warming climate. But heat avoidance behaviors of the butterflies might predominate, rendering thermal tolerance less relevant. In sum, I don't think there is a strong enough basis for the prediction "that butterflies will not be able to compensate for

increased body temperature by raising their thermal tolerance."

• Thank you for raising this. I acknowledge that of course behavioural thermoregulation alters the experienced body temperatures of butterflies (I include thermoregulation capacity as a focus point of the paper) and therefore alters the ecological relevance of upper thermal limits. Similarly, thermoregulatory capacity lacks context without upper thermal limits, which is why I felt both metrics interesting and complementary to include. However, physiological thermal limits are still widely considered an important component of species responses to warming (particularly to extreme heat rather than to gradual warming) as they define the upper boundary condition beyond which behavioural thermoregulation is no longer sufficient (for a recent Nature publication that used upper thermal limits to investigate insect vulnerability to climate change see https://doi.org/10.1038/s41586-026-10155-w). During extreme heat events, behavioural thermoregulation in small insects may not be able to buffer individuals from experiencing extreme body temperatures that approach their physiological limits (e.g., https://doi.org/10.1073/pnas.1316145111, also note this study that recorded butterfly body temperatures during an extreme heatwave in the UK and note that very few datapoints are lower than ambient, and those that are are very close to 1:1 showing thermoconformance rather than an ability to lower body temperature below ambient: https://doi.org/10.1007/s10841-024-00556-5). Previous work of mine has demonstrated a trade-off between thermoregulatory capacity and upper thermal limits in butterflies (see https://doi.org/10.1111/1365-2656.13970), highlighting that in some cases these traits can compensate for each other, which is where the choice of language comes from. I have altered the text however to remove this statement, as it feels too strong given the evidence.

New reviewer comment:
I agree that thermal tolerance is relevant during heatwaves, and the rebuttal comments are mostly reasonable. However, I still wonder about the way in which thermal tolerance was measured. As with the thermoregulation trait, the plasticity of thermal tolerance is not addressed and thus remains an open question. Does individual experience change thermal tolerance? Perhaps more pointedly, does the experience of being trapped and warmed at the rate used in this study reflect the thermal tolerance of each species? Perhaps so, but perhaps not. Species with high thermoregulatory capacity might be able to limit the rate at which a butterfly actually warms up during a real heat wave, perhaps giving the insect enough time to acclimate (increase its physiological tolerance to extreme high temperature). Such a scenario is consistent with "a trade-off between thermoregulatory capacity and upper thermal limits." With respect to evolutionary adaptation, can thermal tolerance change under selection? And more pointedly, does the method used to measure it here reveal any heritable phenotypic variation on which selection can act within each species? Lastly, I appreciate that this thermal tolerance metric has been used previously in other published work, and I don't doubt that it might truly reflect the thermal tolerance of each butterfly species. Even so, I don't see a strong validation of the method, which in turn warrants a more cautious conclusion.

- **As the focus of the project was to compare across and, importantly, within species, all individuals were heated at the same rate and experienced the same protocol, meaning that their measures of thermal limits are directly comparable to each other. Our statements about thermal tolerance in this case are purely comparative. The question of whether this reflects their actual 'thermal tolerance' is an**

interesting question that raises interesting reflections about what exactly 'thermal tolerance' is (and would make for an interesting opinion paper). It is widely known that the method used to quantify thermal limits impacts the result (e.g., https://doi.org/10.1371/journal.pone.0265361), so in this case, all choices made regarding the methods were led by ecological relevance rather than being arbitrarily chosen; using the average air temperature as the starting temperature (and so all individuals were acclimatised to the same common baseline that also reflected real ambient temperatures), using 0.5C per minute as the ramping rate (slow enough to be relevant to real-world temperature changes (sudden large-scale changes are unlikely to reflect real-world conditions), but fast enough so that poor conditions, stress, starvation, etc didn't impact the results, and also fast enough to achieve a reasonable sample size for the given duration of the study. 0.5C is a very commonly used ramping rate in this field, e.g., https://doi.org/10.1038/s41586-026-10155-w), and so reflects other measures of thermal tolerance in this field. Note that there is no perfect measure of thermal limits, however I feel that the methods used allow for a suitable comparison across species to provide ecologically relevant measures of thermal limits.

- **I apologise if I am misunderstanding your comment, but I think you are also asking whether plasticity may differ between species in the sense that some species may have acclimatised to the ramping rate more than others, I apologise if I am misunderstanding. This is an interesting theory, and unfortunately with the data that I have I cannot answer this question. Furthermore, plasticity to heat shock is outside the scope of this manuscript and would require dedicated study (and has already been addressed in other studies, see https://doi.org/10.1038/s41586-026-10155-w plasticity across an elevational gradient, https://doi.org/10.1038/s41467-022-32953-2 showed plasticity effects to be weak and may be generally overestimated). However, regardless of whether individuals acclimatised before or during the experiments, the knockdown temperature is still a knockdown temperature, it is an empirical observation of the temperature at which that individual was no longer able to function. What we report are functional thermal limits under standardised conditions rather than theoretical absolute maximum tolerances.**
- **In insects, it is known that thermal tolerance limits are variable and heritable, so theoretically should be able to be acted upon by evolution (e.g., https://doi.org/10.1371/journal.pone.0103279, https://doi.org/10.1186/s12862-015-0573-0). However you raise a valuable point that this should have been made clearer in the text. I have added a new section to the Introduction to better cover this topic, to explain the phylogenetic conservatism of thermal tolerance in insects, and cite key literature (L102-105). I thank you for raising this oversight.**
- **I welcome the reviewer's specific suggestions regarding what validation would be sufficient, so that I can address this concern as directly as possible, if my response is not satisfactory.**

• To be more specific on this given system, this study took place in open grasslands with little shade or shelter, therefore limiting heat avoidance. Activity would innately expose these insects to heat, so in this case, microhabitats may not be able to buffer species from extreme heat

during heatwaves. In addition, landscapes around the world are being homogenised, and therefore also thermally homogenised, risking disruption to processes such as behavioural thermoregulation in small insects, therefore this lack of response in upper thermal limits to elevation may be of wider interest. Though behavioural thermoregulation can buffer heat exposure, physiological tolerance remains a relevant trait to investigate and compare small ectotherm vulnerability under extreme conditions. Again, to date there is no evidence that butterflies can lower their body temperature below ambient conditions, so should ambient conditions reach physiological limits, behavioural thermoregulation may not be able to buffer this (for example the heatwave that hit Europe in 2025 had air temperatures nearing 50C, above the upper thermal limits of every species tested, unfortunately these heatwave temperatures are increasingly common and extreme). I have added text and references to the Discussion to better highlight this and highlight the role and limitations of upper thermal limits when considering species vulnerability to climate change.

New reviewer comment:
Substrate surface temperatures can differ dramatically even with full sun exposure. Consider the design of green roofs in built environments. Some studies show that a green roof (surface area mostly covered with plants) can have a surface temperature as much as 10 degrees C below that of a non-green roof. The same principle applies to a grassland. A butterfly can position itself in a much cooler microenvironment simply by landing on vegetation, especially if resting on the underside of a leaf or another part of vegetation shaded by leaves. So, while I agree that thermal limits might still be reached during a heatwave, butterflies and other animals have multiple behavioral thermoregulatory options to reduce heat exposure even in non-forested environments. I also grant that it is possible that the method used here to assess thermal limits might reflect such thermal limits of free-living butterflies. However, without further validation of the method, I am not convinced that it does. This is why I don't agree that this method can currently give highly conclusive results with respect to butterfly responses to temperature.

- **Thank you for raising this. Yes, microhabitats (and the microclimates they create) can of course alter the temperatures that insects experience. However, I want to highlight that substrate surface temperatures do not necessarily directly correspond to body temperatures; butterflies are not holding their bodies in contact with surfaces, body temperatures are likely to be more affected by the air temperatures directly above the substrates and solar radiation rather than through direct contact with the substrate (for example see https://doi.org/10.1002/ece3.10623 Figures 5 and 6, which show that adult butterflies show some level of independence in body temperature from the microclimates they occupy (in this case, perches they were found on)). The strong correlations between body temperature and air temperature recorded in this study directly demonstrates this, and is expected within an open sunny grassland with limited opportunities to behaviourally avoid the heat. In this system, while being active, butterflies are flying through the air in open sunny grasslands, competing for resources, looking for mates and egg laying sites etc, continuously interacting with air temperature and absorbing solar radiation, and as you say, their thermal limits may be reached under extreme conditions.**
- **Given that the reviewer has acknowledged that the method may reflect real thermal limits, and that these limits may be reached during heatwaves, I welcome specific**

suggestions regarding what additional validation would be sufficient, as I am committed to addressing this concern as directly as possible.

4. The limited sampling of many butterfly species along the elevational gradient (1000 meters) offers only a weak test of the possibility that the butterflies are responding to climate change via trait adaptation. As such, lack of evidence does not equate to evidence of absence. In Table S2, the sample size of each species per site is not shown. However, with one exception (Erebia aethiops, N = 169 total), the sample size per species per site must be quite small. Aside from this outlier, the larger sample sizes range from 30-61 across 9 sites. It is not reasonable to expect to detect a signature of local adaptation based on 3-7 individuals per species per site. While the analyses are based on traits, which are shared among multiple species and thus may allow the authors to circumvent the sample size issue to some degree, there was no attempt to account for non-independence of traits shared by closely related species (i.e. no phylogenetic comparative approach). Again, it would be valuable to know how phenotypically plastic these thermal traits are. Otherwise, it is difficult to understand their function in relation to environmental variation as well as in relation to other co-functional traits. I see no strong basis for the conclusion "some sort of constraint or limit has been reached" in the evolution of thermoregulatory capacity. Likewise, I disagree with the assertion that this study "demonstrates that butterflies are responding to climate change through redistribution of species rather than adaptation in place." While this study and many before it show evidence for redistribution of species, the evidence here on adaptation in place is inconclusive.

• Thank you for raising this valid concern. Firstly, I want to address your point about the sample size. This study took place within a national park in Germany, where all butterflies are protected by law (and especially in such areas). This national park is also the only part of the Alps in Germany, so other research sites were not accessible for this project. To conduct this research project, permits had to be acquired that imposed limits on numbers of butterflies that could be caught within a strict time limit that was set by the local governing authority. As such, unfortunately it is not possible to increase the sample size further, though I acknowledge that smaller sample sizes reduce statistical power to detect responses. Unfortunately, sample size limitations are very common in field studies of wild populations, particularly on protected species in protected areas.

New reviewer comment:
I appreciate that it was not possible to obtain large sample sizes of each species in this study. I want to be clear that I am not asking the author to increase the sample sizes. My point is that most of the sample sizes per species per site are small, thus there is limited power to detect a signature of trait adaptation. I am asking the author to interpret the results accordingly.

- **Thank you for raising this point. The caveat that larger sample sizes may show different results and caution should be used for interpretation is on L598 and L605-609. Throughout the manuscript, the language has been changed to better reflect the evidence and caution against strong conclusions.**

• I want to explain how the analysis should have helped to control for this. Species was always included as a random effect, rare species should therefore contribute to the overall relationships, but not get much weight in estimating their species-specific intercept. However, I feel this is important to empirically test. I have added a series of checks in the analysis to test for the impact of species-specific sample size on the results (i.e. were rare species obscuring patterns visible in the more abundant species). First, I remove all rare species (sample size < 10) and refit the models. This showed no difference in coefficient estimates strength or direction. For a stronger check, I then did a k-fold cross-validation, comparing how the model predictions were impacted by sample size (again comparing the full dataset to only species with >10 observations), here again coefficients were consistent in strength and direction, and the full model predicted slightly better. I checked whether the differences between the models were because of just sample size, or whether the rare species themselves were contributing something meaningful, so I ran another k-fold CV comparing a model trained on only the most abundant species against a random subsample of the abundant species but at the same sample size as the full dataset. These two performed very similarly, and both outperformed the subset model, indicating that sample size marginally improved the model predictive power, and the inclusion of rare species did not alter the performance. I did something similar for the Cox models, where I used the concordance index instead, and again found very little difference between the models (difference in C-statistic of 0.002). I have added this to the Methods section for both analyses as a robustness check, and it can be found in the associated code. These tests can only tell me whether or not the sample size per species is impacting the models, I cannot explicitly test for whether more data (that I don't have) might change the results. However, one thing I can do is as you mention, E. aethiops has a high sample size across the gradient. I have tested this species in isolation for both analyses, and the results are consistent with the full models (no change in thermoregulatory capacity across the gradient; no change in upper thermal limits across the gradient). If it would be valuable, I could include this in the manuscript, at present it is only present in the code (which will be published alongside the manuscript). I am open to your suggestion on this.

New reviewer comment:
Thank you for adding the new analyses. They convinced me that phylogenetic non-independence is not influencing the results or conclusions here. Adding the analysis of E. aethiops would be the most powerful way to demonstrate no evidence of local adaptation in thermoregulatory capacity or thermal tolerance across the elevational gradient. However, I still believe additional methodological (experimental) approaches would be needed to provide a strong test of the local adaptation hypothesis.

- **I have added a plot of Erebia aethiops as an example species to highlight the findings within species by adding it as a panel to Figure 2. Note that this is the same figure as is in the large panel plot in the SoM. If it would be of interest, I could also add the analysis of Erebia aethiops alongside the rest of the analysis, either to the main text (if this would not add to much length) or to the SoM. I am open to the reviewer's suggestions on this.**

• _I have also included phylogenetic sensitivity checks for each analysis (comparing non-phylogenetic models with phylogenetic models, and testing for phylogenetic signals in the response variables), which have been included in the Methods. Something of note is that the

results found in this project align with the existing literature, where no difference in thermoregulatory capacity has been found across much wider geographic comparisons (e.g. comparing same species in UK and Spain: https://doi.org/10.1111/1365-2656.14039 and UK and Panama (https://doi.org/10.1111/gcb.16797). However I feel it is important to be upfront with the reader about the sample sizes, so I have added a caveat to the Discussion to highlight that larger sample sizes may allow for greater possibility of detection. Please note that the vast majority of species were not found across the entire gradient (with a few notable exceptions such as E. aethiops and interestingly L. coridon, and a few others).

New reviewer comment:
The phylogenetic sensitivity checks are helpful. Perhaps it would be worth arguing that the similarity in thermoregulatory capacity found across much wider geographic comparisons suggests that this trait is not likely to show local adaptation at a smaller geographic scale. Even so, this doesn't mean that other relevant traits are not adapting to climate change. Furthermore, if there is adaptive plasticity of thermoregulatory capacity, the observed comparisons might be obscuring it.

- **I agree that the evidence gathered to date does suggest that thermoregulatory capacities seem to lack variation across geographical regions. To date there have been two comparisons of butterfly thermoregulation capacity across regions (as previously linked, UK and Spain (sharing many species), and UK and Panama (sharing no species). To date, there have been no consistent differences (but in the UK and Panama comparison, there were some minor differences, in that Panamanian butterflies showed a better ability to avoid high body temperatures than British butterflies (shallower body temperature ~ air temperature slope), though it is worth noting that this was being driven by morphology and taxonomy rather than inherent differences, again suggesting that thermoregulatory capacity is being driven by species identity and traits rather than adaptation to local climatic conditions). However, these comparisons are all across very large geographic areas, non-mixing populations, and there are many other differences between these regions than just temperature. The purpose of this study was to investigate this more closely by controlling for impacts of weather, land-use, landscape/habitat structure etc by using a continuous climate gradient within a small area (all sites are within 20 km of each other) of similar comparable habitats. As such, it is the first test of continuous change in thermoregulation in butterflies, looking both within and across species. The results here support what has been previously found, that thermoregulation patterns seem to be relatively consistent in butterfly species. Of course there may be other traits that are adapting in response to climate change, however I cannot comment on those, as they were not the focus of this study.**

• _In this case I found no evidence for consistent shifts in thermal traits across the gradient within this study system, which goes against my hypothesis of species optimising these traits in suit their local climate. Instead it appears that these are intrinsic properties of species with limited capacity to change, at least within the given system. However, we did detect strong interspecific differences according to size and colour, which aligns with the later results of species redistributions (tracking the climate rather than adapting in place), as well as historic

trends from paleoecology of insects generally redistributing rather than adapting in place. However, I want to make it clear to the reader that in this case, we cannot exclude the possibility that there is something occurring here, but that we just have insufficient evidence to suggest that species do not adjust their thermoregulatory capacity (similar to all other studies on butterflies), but that a more detailed analysis with a higher sampling intensity may detect different patterns. I have added text to the Discussion to highlight this important caveat, and thank you for raising this concern.

New reviewer comment:
I am convinced by the evidence in this study and many other studies that a major butterfly response to climate change is species redistribution. Indeed, it is this pattern and the high dispersal ability of most butterfly species that leads me to wonder to what extent these species are able to evade selection from climate change on other phenotypic traits (such as thermoregulatory capacity and thermal tolerance).

- **This is an interesting idea, thank you for raising this, I have added text to the Discussion to highlight this (L829-835).**

Line-specific comments:

- **Thank you for providing detailed comments and recommendations, I am grateful for the level of detail provided in helping to improve the manuscript.**

Line 23. Add "of butterflies" before "across an elevational gradient…"

- **Added, thank you.**

Line 25. Change "are fixed" to "might be fixed."

- **Added, thank you.**

Lines 25-26. Better wording would be: "At low elevations, large species were better than small species at avoiding high body temperatures."

- **This has been changed as you suggested, thank you.**

Lines 28-29. The meaning of this sentence is not clear.

- **This has been reworded to improve clarity, this result is hard to concisely describe but I have attempted to remain consistent in the use of language (i.e., thermoregulatory capacity for body temperature ~ air temperature relationship). Please let me know if this sentence is still unclear.**

Line 32. Remove "rather than adaptation in place" because there is not enough evidence to draw this conclusion.

- **I am hesitant to remove this part of the sentence as all of the evidence presented in this manuscript points to a lack of local response in the specific traits we tested across the elevational gradient, and supports reliance on redistribution over adaptation in place (framed in the commonly used 'responses to climate change call in to these two categories' narrative). To maintain this narrative structure, I have not removed this text. However the abstract has been reworded to reduce the overly strong conclusions being pushed, I hope this satisfies the reviewer.**

Line 33. Add "ecological" before "responses to climate change."

- **This has been added, thank you.**

Lines 48-49. Because local extinction is not a mechanism species rely on, the juxtaposition of these two sentences is problematic. Perhaps, "Crucially, whether species rely on dispersal or adaptation in place has direct conservation..."

- **Upon re-reading this I see how this line is confusing, thank you for raising this. I have changed the line as you suggested.**

Line 59. Change "upscale" to "scale up."

- **This has been changed, thank you.**

Line 62. The comma after "equally" should be a semicolon.

- **Thank you for raising this, this has been corrected.**

Line 66. Consider the following for the citation here: Warren et al. 2018, Science 360: 791-795.

- **Thank you for sharing this citation, it is very useful here and has been added.**

Lines 93-95. I agree with this final sentence of the paragraph, but I didn't see much follow-up to this expectation in the Discussion.

- **Thank you for raising this. Yes I expected to see stronger behavioural responses (thermoregulation) compared to thermal tolerance in insects, however the data we have collected does not support that theory.**

Lines 137-138. The constraint interpretation would be true if the thermal traits don't change and the organisms suffer for it. There's no evidence here that the butterflies are constrained in adaptation because there is no measure of phenotypic selection.

- Here I was using the term constraint in a broader ecological sense to describe a limit to the range, direction, or rate of possible responses, however I agree that this statement is too narrow in scope and does not highlight the other possible reasons for seeing no change. I have changed this sentence to better reflect the possible reasons why no change may be observed.

Line 194. Flexibility in thermoregulatory capacity was not measured or analyzed, as far as I can see.

- I am concerned that this is not clear at this point, this is one of the key focuses of the manuscript, to test whether the thermal traits of butterfly species show responses to local environmental conditions (both thermoregulation patterns, and thermal tolerance, research question 1). This was explicitly tested throughout the manuscript. Please let me know what could be changed to make this clearer.

Lines 196-197. This is where it would be worth mentioning the alternative hypothesis that species with greater upslope shifts may have avoided selection on thermal traits. This hypothesis can also explain the negative correlation between thermoregulatory capacity and observed elevational range shift.

- Thank you for raising this, this is a really interesting point about the direction of causality. I initially added text here but found that it was better placed in the Discussion where I could go into more detail, so I have added text to the Discussion (L830-835) to highlight this more clearly, and kept the Introduction more focused on just the overall topic of investigating change (and tried not to speculate as to reasons why in the Intro and kept this to the Discussion to reduce length and repetition).

Line 197. I believe "rapid heating" refers to thermoregulatory capacity. Please correct this language.

- This has been corrected, thank you.

Lines 214-215. Describe the systematic process used to sample butterflies.

- Further information has been added to the Methods (L223-235).

Lines 261-263. If there is justification, provide it to validate the claim that a heating rate of 0.5 degrees C per minute over 90 minutes simulates a heat wave.

- Thank you for raising this, this line is definitely unclear. The intention was to highlight that rapid ramping rates have limited ecological relevance, whereas slower ramping rates may reflect real-world temperature changes these organisms experience in their habitats, and so thermal tolerances derived from these rates may have more ecological relevance. This line has been changed to remove this claim and be more clear as to the intention behind this decision, and citations have been added (note that citations for some reason are not highlighted as tracked

changes when added using my citation software, I apologise for that inconvenience).

Lines 348, 395, 485. Replace "fall" with "knockdown" for clarity.

- **Thank you for raising this, 'fall' has been replaced with 'knockdown' throughout the full manuscript.**

Line 356. This AIC difference looks quite large. Is the meaningful difference here not 2? Why not?

- **Thank you for noticing this, this was a typo. I am grateful this was raised, the actual AIC difference is 1.9 not 11.9.**

Line 414. Remove "heated up faster" and replace with accurate language.

- **This has been corrected, here and throughout, thank you.**

Lines 422, 426, 431. Remove "heated up more slowly" and replace with accurate language. Line 425. Remove "heated up more quickly" and replace with accurate language.

- **Thank you for raising this, these have all been corrected.**

Line 539. Leading the Discussion section with this statement is giving it more credence than is warranted. I strongly suggest restructuring the Discussion section so it 1) leads with the strongest evidence (associations between elevational range shifts and traits measured in this study), and 2) explicitly addresses the questions and hypotheses given in the Introduction section. There is a lot of speculative content in the Discussion that goes too far beyond the evidence.

- **The first paragraph of the Discussion (the summary of results) has been edited to better reflect the evidence, and is structured according to the order of the research questions laid out in the Introduction.**

Line 542. This is the first of several cases where the author is equating elevation and temperature without acknowledging the many other factors that are probably (or known to be) associated with change in elevation. Many such factors might be abiotic, such as precipitation and soil type, which in turn affect many biotic factors, most obviously the plant community that butterflies depend on.

- **While I acknowledge that the plant community composition can influence butterfly species presence through the availability of larval foodplants, it is unlikely to affect the thermoregulatory performance of adult butterflies. Adult butterflies are highly generalist nectar feeders and their thermal physiology is determined by factors such as morphology, behaviour, and physiology rather than the specific plant species composition of their habitat. Additionally, due to the highly mobile nature of adult butterflies, it is unlikely that adults remain in the exact locations at which**

they developed, and so larval foodplant quality for example is unlikely to bias the results. What is likely most relevant to adult thermal performance is the structural composition of the vegetation, whether it provides basking sites or shade/shelter, and in this regard all sites were functionally equivalent, being open extensively cattle-grazed grasslands with similar vegetation structure. All sites were selected based on the similarities of their vegetation community structure. Furthermore, being so close together (less than 20km apart), weather conditions are unlikely to substantially differ between the sites. Finally, the inclusion of site as a random effect in all models should control for the impacts of other factors that vary between sites not explained by their elevation. It is possible however that other abiotic conditions that are known to vary across elevational gradients (such as wind speed, see https://doi.org/10.1029/2009GL042255) may impact thermoregulatory performance. However, climate change is known to be reducing wind speeds, and so the lower wind speed found at lower elevations further supports low elevations making suitable future climate proxies compared to current conditions (note climatic conditions, not just temperature). However, it is important to note that in this region of the European Alps it is dry, and so the temperature gradient is quite strong compared to more humid or tropical regions (temperatures in the Alps have been shown to differ as much as nearly 10C across 1000m (see https://doi.org/10.1175/1520-0442(2003)016%3C1032:SASVOA%3E2.0.CO;2), the elevation gradient used in this study, quite a lot for a small insect that relies on temperature for fitness related activities). Therefore, it is unlikely that changes in wind speed are going to have as strong impacts on the insect communities than the differences in air temperature. This is further supported by the lack of effect of weather conditions on thermoregulatory performances.

Lines 545-547. The effect of wing condition here is likely to be due to insect age. This point is discussed much later and at greater length than needed. It would be most efficient and informative to mention here.

- This is the first reported finding (to my knowledge) of butterfly thermal tolerance changing with age within the adult life stage. This has been reported rarely in other holometabolous insects, and warrants further study. I don't feel that addressing this in the summary paragraph is a suitable place for this, however to make this more concise I have cut text on this topic in the Discussion (L672-675).

Lines 552-555. As mentioned previously, the association interpreted here doesn't tell the direction or nature of causality. Perhaps the species that didn't move upslope as much have evolved higher thermoregulatory capacity. Is there evidence in this study or elsewhere that rules out this alternative interpretation? If so, this is the place to state it.

- I apologise I'm not quite sure which lines you are referring to. The Lines you quote include: 'There was evidence of thermal and functional traits predicting responses to recent climate change, whereby species with poor thermoregulatory performance or large wing lengths showed greater upwards elevational range shifts.' This is from the summary paragraph at the start of the Discussion, and is just

re-summarising the results. Interpretation for this comes later (the Discussion follows the structure of the research questions laid out in the introduction, as this is the final research question this can be found in L832-855).
- **For your theory, absolutely there is the question of directionality. But this cannot be answered without, for example, a time-series of data on thermoregulatory capacity of butterflies in regions such as this and investigating change over time. As far as I'm aware, this data doesn't exist for any butterfly system. However I want to be clear that I did not mean to imply causality, I found an association and I am here summarising it and later on, discussing possible explanations. Later on in the Discussion I have added in text to better highlight possible directions and more explanations than I had previously (as you suggested earlier), L841-847).**

Lines 557, 562. Remove "heated up at similar rates" and "rate of heat gain" and replace with accurate language.

- **This has been corrected, thank you.**

Lines 564-565. I suggest not invoking a forecast of average global cloud cover. It's not very convincing. Climate change forecasts of precipitation and cloud cover patterns for any given part of the Earth at any given time of year are questionable. Weather forecasts can do this with reasonable confidence, but not climate forecasts.

- **I agree that precise forecasts of cloud cover changes can be uncertain, I have changed the text here to reflect this and just highlight the importance in considering changes in solar radiation and its impacts on butterfly thermoregulation as a topic valuable for further study.**

Line 570. Replace "heating rate" with accurate language.

- **This has been corrected, thank you.**

Lines 571-573. I disagree with this conclusion for reasons discussed above.

- **See next comment for my response.**

Lines 573-574. This conclusion is also unwarranted without ruling out the alternative discussed above.

- **I respectfully disagree that this conclusion is unwarranted. The observed association between thermoregulatory capacity and elevational range shifts, combined with the lack of intraspecific differentiation across the gradient together supports the inference that this trait shows limited flexibility and may therefore confer vulnerability. The language used, 'may not', 'may be' already explicitly acknowledges uncertainty. I would note that the alternative hypothesis raised is not inconsistent with this conclusion, and I discuss the limitations of causal inference in more detail later in the Discussion.**

Lines 586-588. I agree that the evidence is insufficient. I suggest adding that this means the evidence is inconclusive on this point, so readers do not jump to conclusions.

- **I respectfully note that the existing text already states explicitly that the evidence is insufficient and that we cannot exclude the possibility of intraspecific adaptation in other species or systems. I believe this already clearly conveys the inconclusiveness the reviewer is requesting without implying readers should disregard the findings of this study entirely. I am satisfied that the text strikes an appropriate balance between reporting a genuine finding, that there was no evidence of intraspecific adaptation in this system, and acknowledging the limitations of what that finding can and cannot tell us.**

Line 608. Change "copes" to "cope" for grammatical agreement.

- **Thank you for raising this, this has been corrected.**

Lines 651-652. Replace "performed similarly" with a more specific statement.

- **Thank you for raising this, this has been reworded to be more clear.**

Lines 671-672. It would be clearer to say that dark species showed higher thermoregulatory capacity at low elevations.

- **Thank you for raising this, this has been corrected.**

Lines 674-676. I don't follow the reasoning here. This speculation seems to go too far beyond the evidence.

- **The results show that species themselves are not responding, but at low elevations, communities tend to contain dark species that have improved thermoregulatory capacities. The change detected (Figure 2) shows that across the elevational gradient, instead of species changing their thermoregulator capacities, they are instead replaced by other species with different thermoregulatory capacities, specifically the dark and large species. The lack of intraspecific response but interspecific response within the functional groups is what drives this sentence. Thank you for raising this as an unclear point, the Discussion has been reordered and a new section written to better illustrate this point (L680-687).**

Lines 679-680. This interpretation is too strongly worded. There was no measure of performance other than the thermal tolerance assay, which is not well validated as discussed above.

- **Upon re-reading this sentence I think again this is a language issue, here the word 'better performing' is referring to thermoregulatory performance, but you have another interpretation of that word. I am using the word 'performance' in the**

broader ecological sense of functional effectiveness. To improve clarity, this sentence has been reworded to avoid the use of ambiguous language.

Lines 682-683. This conclusion is weak because the association between wing darkness and elevation is confounded by other factors.

- The thermoregulatory capacity data provides a functional link that most studies showing elevation-colouration gradients don't have, I respectfully disagree that the conclusion is weak. However, this statement is overly strong, and so this has been rewritten to better reflect the associations identified in this study (L728-732).

Lines 691-692. Can a cline be interspecific? This language seems to dismiss the many other traits and properties that differ among species.

- Thank you for raising this, I agree that cline is not appropriate terminology here and has been replaced.

Line 709. Replace "hotter climatic conditions (lower elevations)" with "lower elevations." It's important not to risk misattribution. This is one of the key points in Evans et al. 2025, Nature Climate Change.

- Here and throughout the Discussion, I have replaced statements like this with just the elevation. I hope this reduces the risk of misattribution.

Line 714. I'm skeptical that small butterflies would not be able to disperse elevationally, especially on the order of 1000 meters.

- The question is not whether it is possible, but whether we have any evidence that this occurring. In this system, we have evidence that larger winged species are showing greater elevational range shifts than smaller winged species. In this particular region, dense dark forest surrounds these small patches of open grasslands, potentially acting as non-permeable barriers to dispersal for forest-avoidant small species.

Lines 718-720. This statement goes too far beyond the evidence.

- This sentence has been reworded to better reflect the evidence (L772-775).

Lines 723-725. This statement goes too far beyond the evidence.

- New text has been added to the Discussion to highlight and discuss this (L680-687), and this statement has also been changed to better reflect the evidence (L780-784).

Lines 725-726. I'm confused. In previous passages, the argument was that small species are most vulnerable to extreme heat. This seems to be saying the opposite.

- The previous passage is focused on the vulnerability of small species because of their lack of response, this section is now focusing on vulnerability through different mechanisms that are driving responses and species sorting. Essentially that small species are vulnerable because they may not be able to respond, but sensitive large species have the capacity to respond. I can see how there is confusion moving between these sections, as the switch is not made clear in text. I have added text to better highlight these two concepts (L777-788).

Lines 704-791. I suggest cutting out a lot of the speculative content here. The Discussion section should be shortened considerably by editing out the most speculative content here and elsewhere.

- This section has been reworked and much text has been reduced, but I am hesitant to cut out too much content, particularly as much of this has been added to satisfy other reviewer comments in the previous round of revisions. The remaining text is more tightly focused on the key results. However if the reviewer could be more specific about text that could be cut I would be open to further suggestions, so long as it does not impact my ability to satisfy other reviewer comments.

Line 797. Be more specific and precise instead of "thermal and functional traits..."

- This has been changed to be more specific, thank you.

Lines 799-800. The capacity of species to adapt was not tested.

- Here, as stated in the introduction (L42), 'adaptation' is used in the broad sense of any type of response in traits that would allow for persistence in place, rather than redistribution to track their niche. In this manuscript, adaptation was specifically tested and no response was found across the elevational gradient.

Lines 815-821. Conservation planning generally accounts for species redistribution. Focus on how this study specifically informs conservation planning in the area of this study and leave out the speculative parts. Alternatively, if speculative points are retained, it is important to state the level of confidence based on the science. Which conclusions can be extrapolated beyond the study area and why?

- Thank you for raising this, this section could definitely have been more specific to the study system in question. Further text and qualifiers has been added with site-specific recommendations to avoid overgeneralisations (L885-899).

Line 841. I do not agree that this is a mechanistic study. While traits are identified, the processes by which these traits affect patterns are unclear.

- Thank you for raising this, this is a valid point about the language used. 'Mechanistic' has been removed to better reflect the associations identified in this study.

Lines 845-846. This conclusion (now repeated multiple times) is not founded by the evidence here.

- **I respectfully disagree, I have addressed this comment in more detail in previous comments.**

Lines 847-849. This expectation has a weak basis. It is not clear if the result underlying this conclusion is context-dependent. My skepticism stems from the fact that there are many small, dark-colored butterfly species in desert environments, which have had extreme climate for long periods of time.

- **I apologise for my confusion but I am unsure which part of the sentence exactly this is referring to. Is this about the sentence 'The diversity of thermoregulatory abilities identified may go towards explaining the complex patterns of 'winners' and 'losers' to climate change, and could help identify vulnerable species and functional groups.'? I don't believe this statement goes against the fact that small dark butterflies can exist in hot climates (although please keep in mind that darkness in the visual spectrum does not necessarily mean high near-infrared absorption which is the metric of colouration used in this study, as seen in L. coridon). In relation to this specific statement, the purpose of this sentence is to highlight that butterflies are diverse in their thermal traits (as seen in Figure S2-3 in the SoM), perhaps going towards explaining the diversity in their responses to temperature change. Particularly as thermoregulation capacity seems to be particularly linked with redistribution potential, I personally feel this sentence stands. I am happy to take guidance from the editor on this. Text has been added to the Discussion to highlight that the results presented in this study may not hold for other systems, however.**

Reviewer #3 (Remarks to the Author):

I appreciate the author's rebuttal comments and revisions, which have greatly improved the manuscript. During this read I became more convinced than ever that this study makes an important and impactful contribution to climate change ecology. However, I remain unconvinced that this study provides conclusive evidence for or against "adaptation in place." As the author states, we simply respectfully disagree on this point, which is fine with me. I recognize that my opinion on this point is in the minority among the reviewers. Nevertheless, some of my comments below relate to this point of disagreement. However, most of my comments are aimed at helping the author improve the writing.

- Thank you very much for your thoughtful and detailed comments. I am grateful for the time and effort these reviews take, and I'm thankful for the opportunity to improve this manuscript. I have responded to all your comments below and made all suggested changes. I hope you feel this has further improved the manuscript.

Specific comments
1. Title. If it were up to me, I would choose a title that best captures the most conclusive and impactful result of this study, probably something like: "Butterfly species with low thermoregulation capacity show strongest redistribution patterns in the Bavarian Alps." As I have argued previously, I do not agree that there is strong evidence for the "not adaptation" part of the title because I think the methods of evaluating this point are not strong enough to yield conclusive results.

- Thank you for this suggestion, I agree that the title could be rewritten to better reflect the key findings. I have changed the title in a way that I hope better reflects the manuscript while not leaning too heavily on adaptation.

2. Line 32. Along the same lines as my previous comment, I suggest replacing "demonstrates" with "argues" because I do not agree that the evidence demonstrates the "rather than adaptation" part of the sentence.

- Thank you for raising this, I agree that it is important to not overstep the results and this wording is too strong. I have changed this to 'argues' as you suggested.

3. I think the Introduction is quite strong now. Nice work!

- Thank you very much for your kind words!

4. Line 70. Replace "their" with "its" for grammatical agreement.

- Thank you for raising this, this has been corrected.

5. Line 332. Remove "that" for grammatical accuracy.

- Thank you for raising this, this has been corrected.

6. Figures 3, 4, and 6. I suggest replacing the heading above each graph with a more descriptive title. For example, the three graphs in Fig. 3A would be clearer if the headings

were: Low elevation, Intermediate elevation, High elevation. The color coding and the legend already provide the information in terms of scaled values, making the current headings redundant and less informative than they could be. In Fig. 6, I suggest adding the scaling information in the caption, as in Figs. 3 and 4.

- Thank you for this suggestion. Just to add that there is no scaling in Figure 6 (the three panels of survival curves), so the caption has not been changed in this case. In all the faceted plots however, the headings have been changed to improve clarity, as well I have changed the colours of the boxes and text to make it easier to read.

7. Line 534. Replace “their” with “its” for grammatical agreement.

- Thank you for this correction, this has been changed.

8. Figure 8 caption. Because the color version of the figure does not have a grey ribbon, I suggest saying “shaded ribbon” instead.

- I apologise for this oversight, this should definitely read ‘coloured’, this has been corrected.

9. Line 567. I suggest replacing “getting” with “being” for accuracy.

- Thank you for this suggestion, this has been changed to ‘being’.

10. Line 577. I suggest replacing “they” with “butterflies” for clarity.

- Thank you for this suggestion, this has been changed.

11. Line 581. Remove the erroneous space and period.

- Thank you for noticing this, this has been removed.

12. Line 597. In keeping with my stance on how well this study discounts adaptation, I suggest replacing “support the hypothesis that” with “determine if.”

- Thank you for raising this. Respectfully, I feel that the text here already conveys what you suggest, and the line stands as true in this case, there is insufficient evidence to support my initial hypothesis that butterflies would alter their thermoregulatory performance in response to local climatic conditions (it was my intention with this line to bring it back to the original hypotheses as they were stated in the Introduction). But I agree that the wording is overly strong considering the evidence, the sentence has been rewritten, in particular to avoid the term ‘adaptation’ at all, as this can convey different meanings to different people.

13. Discussion section. Some parts are written in first person singular (“I also found” in line 606), while others are written in first person plural (“we have insufficient evidence in line 596).

Whichever voice is accurate should be used consistently throughout.

- Thank you for raising this. This was from a previous round of comments that suggested I use ‘I’ instead of ‘we’ due to being a single author, however I have to admit, upon rereading, I find ‘I’ to sound a bit strange and felt more comfortable using a less active voice where possible. I have used ‘we’ only when referring to the broader scientific community and not specifically about myself as the author (e.g., ‘we may expect to see stronger changes in [behavioural traits than physiological traits]…’, this wouldn’t sound correct to say ‘I may expect to see’ outside of the hypotheses (this specific example in on lines L95-96)). I have made corrections throughout to change ‘we’ to ‘I’ where appropriate, and to further use a passive voice throughout where possible (including the line you mentioned).

14. Line 618. I suggest replacing “eroded” with a more literal verb for the sake of clarity.

- Thank you for raising this, this has been changed to ‘reverted’ for clarity.

15. Line 767. Replace “level” with “levels” for grammatical agreement.

- Thank you, this has been corrected.

16. Line 779. Delete “compared” at the end of the sentence. I think this must be a typo.

- Thank you for noticing this, yes this was a typo. This has been removed.

17. Lines 789-791. This interpretation emphasizes the increased wing length at low elevation.

Alternatively, one could emphasize the decreased wing length at high elevation. It’s not obvious from Fig. 7B which of these perspectives might be supported by the largest deviation from the mean. I would guess that higher elevation individuals tend to have reduced size because of the short growing season for larval development (which selects for smaller final size) and lower quality larval host plants (especially leaf water content), but this is merely speculation. I leave it to the author to decide if this point is actionable.

- Thank you for raising this. This is a valid point, this has been discussed in greater length in lines L766-781. The evidence for changes in food plant quality are variable and complex (patterns depend on plant guild and region), so I have left this specific part out, but otherwise your ideas are described in the previous section.

18. Line 800. The author’s addition of “extreme weather” to the slow-growth-high-mortality hypothesis is novel, as far as I know. If other authors have included this potential source of mortality, those sources should be cited here. The Benrey and Denno 1997 paper does not include consideration of any mortality factors other than natural enemies of the herbivore, which is also the intent of the original hypothesis in Clancy and Price 1987.

- I included this as another expectation of how climate change may disproportionately impact slow-growing insects, I did not intend for it to be implied as a part of the existing slow growth high mortality hypothesis, the poor placement of the reference here is confusing. I haven’t been able to find sources to support this expectation, so I have moved the reference to earlier in the statement and changed the wording to better reflect that this is a theory I am proposing rather than a part of the hypothesis.

19. Line 802. Remove “for” because it is grammatically awkward and unnecessary.

- Thank you for raising this, this has been corrected.

20. Line 858. I suggest ending the sentence after “…(Evans et al. 2025)” and starting a new sentence with “Here I argue…” Alternatively, a semicolon would work if this is kept as one (very long) sentence, but a comma doesn’t work grammatically.

- Thank you for noticing this overly long sentence, this has been split in to two sentences as you suggested.

21. Line 860. Replace “their” with “its” for grammatical agreement.

- Thank you for noticing this, this has been corrected.

22. Line 861. Remove “spatial” because it is redundant.

- Thank you for noticing this redundancy, this has been removed.

23. Line 868. Consistent with my stance expressed throughout, I suggest replacing “adaptive potential” with “variation.” It's not clear if this is adaptive potential because there may be underlying genetic or plastic capacity that was not observed here. For example, if the association between range shift and thermoregulation capacity is due to butterflies shifting their geographic ranges and thus avoiding selection on thermal traits, the adaptive potential of many species has not been tested (by the researchers or by recent natural selection from climate change).

- Thank you for raising this. It is important that the text is in line with the findings, and so ‘adaptative potential’ and ‘adaptation’ has been changed to ‘variation’ or ‘response’ throughout the text in all places except where adaptation is specifically being referred to (such as in the Introduction where the concept is first raised).

24. Lines 870-871. Replace “butterfly communities will contain assemblages that differ” with “butterfly assemblages will differ.”

- Thank you for raising this, this has been corrected as you suggested.

25. Line 872. The “disproportionate losses of small and dark species compared to large and pale species” is an expectation from the region studied here but not necessarily elsewhere. I suggest adding “in the Alps” at the end of this sentence.

- Thank you for highlighting this, this has been added to make it clear that these expectations are limited to this study system.
